# A Family of Symmetrical and Unsymmetrical Aza‐Dipyrromethenes and Aza‐BODIPYs with Through‐Bond and Face‐To‐Face π‐Interaction between Termini

**DOI:** 10.1002/chem.202501862

**Published:** 2025-07-14

**Authors:** Budur N. Alanazi, Ahad O. Alsahli, Sonia Remiro‐Buenamañana, Alejandro Díaz‐Moscoso, Faeza H. Alkorbi, Norah A. Alsaiari, Conor Marrett‐Munro, Isabelle Chambrier, David L. Hughes, Simon J. Coles, Graham J. Tizzard, Andrew N. Cammidge

**Affiliations:** ^1^ School of Chemistry Pharmacy and Pharmacology University of East Anglia Norwich Research Park Norwich NR4 7TJ UK; ^2^ UK National Crystallography Service School of Chemistry University of Southampton Southampton SO17 1BJ UK

**Keywords:** BODIPY, chromophores, helicene, switching, synthesis

## Abstract

The dipyrromethenes (DPMs) and their borylated adducts (BODIPYs) constitute a class of versatile chromophores that has become one of the most widely studied over recent decades. They combine excellent photochemistry properties with opportunity for synthetic manipulation and tuning. We report here a related class of aza‐dibenzodipyrromethenes and show that they present an interesting architecture where the core adopts a helical arrangement that places terminal aryl functional groups directly on top of each other in close, π‐stacked arrangement. Complexation by reaction with boron trifluoride, demonstrated to be significantly improved by addition of trimethylsilyl chloride, induces a stereochemical inversion that destroys the helix, flattens the system, and switches on fluorescence. A wide range of terminal aromatic fragments can be easily introduced. Unsymmetrical derivatives can be made conveniently by simple mixed condensations, but a controlled, rational approach is also described whereby the ability of one component to homocondense (the more reactive partner) is removed by conversion to its corresponding tosylate or triflate. The two approaches have been investigated through successful introduction of complementary electron rich, electron poor, and π‐extended functional termini.

## Graphical abstract

The reported aza‐dibenzodiprromethenes present a rare helical architecture, placing terminal aryl groups in a close, π‐stacked arrangement. Differentially terminated derivatives are prepared by statistical condensation or controlled synthesis via conversion of one partner to its tosylate or triflate. Borylation (BF_3_) is significantly improved by addition of TMS‐Cl; an important protocol change that could improve general BODIPY syntheses. The alkene stereochemistry switches in the formed aza‐BODIPYs, separating the termini.

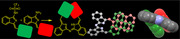

## 1 Introduction

**Figure 1:**
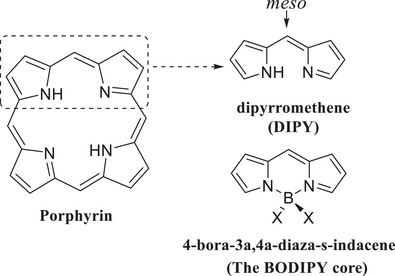
Core structures of porphyrin, DIPY, and BODIPY.

Porphyrins are an extensively studied class of aromatic macrocycle that comprise four methine‐bridged pyrrolic units. They are widespread in nature and perform diverse and crucial biological functions. Synthetic analogues are useful and versatile functional materials, and their chemistry has been comprehensively reviewed.^[^ ^1^ ^]^ Porphyrinoid fragments, where two pryrrolic units are joined by a conjugating single atom bridge, form a separate and distinct class of materials. When the bridging atom is carbon, the molecule is a dipyrromethene (DIPY), and when further bonded to boron via the pyrrolic nitrogens the BODIPYs are produced (Figure 1).

The first BODIPY (boron dipyrromethene, or formally 4‐bora‐3a, 4a‐diaza‐s‐indacene) was reported by Treibs and Kreuzer in 1968,^[^ ^2^ ^]^ but this general class of material has seen accelerated attention in the last 30 years. The interest stems from their favorable characteristics (particularly optical) and their synthetic versatility that allows tuning of properties. They are generally robust, can be soluble in common solvents, and have high absorption in the visible range.^[^ ^3^ ^]^ Many are emissive with high quantum efficiencies, including enhancements in the condensed phase through aggregation‐induced emission.^[^ ^4^ ^]^ These properties have naturally seen applications developed across many areas, including solar energy conversion,^[^ ^5^ ^]^ biological applications in both imaging^[^ ^5^, ^6^, ^7^ ^]^ and therapy,^[^ ^4^, ^6^ ^]^ and (photo)catalysis.^[^ ^8^ ^]^

**Figure 2:**
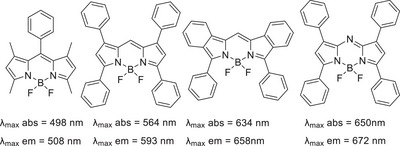
Examples of structural modifications in BODIPYs leading to tuning of absorption and emission wavelengths.^[^ ^9^, ^10^, ^11^, ^12^ ^]^

Tuning of properties and optimization can be achieved through structural variation on the parent (BO)DIPY via a number of complementary approaches. Substituents can be introduced on one or more of the free pyrrolic carbons, and conjugating substituents significantly shift the absorption to longer wavelengths.^[^ ^10^ ^]^ π‐Extension through (benzo)fusion is similarly effective.^[^ ^11^ ^]^ Substitution at boron offers additional versatility, although the effects on optical properties are, as expected, more modest. The final point of modification is the bridging *meso* carbon. Substituents on this position also have only a moderate impact on the optical properties because they only weakly interact with the π‐system. However, replacing the *meso*‐carbon itself with nitrogen, giving azaDIPY/BODIPY, has a pronounced effect (Figure 2).^[^ ^12^ ^]^ In fact, these aza‐analogues, which can be viewed as porphyrazine^[^ ^13^ ^]^ fragments, predate BODIPY itself, having been discovered in 1943 by Rogers.^[^ ^14^ ^]^ The synthesis of aza‐DIPY and aza‐BODIPYs was only refined and reported much later by O‐Shea,^[^ ^12^, ^15^ ^]^ initiating sustained renewed interest in these *meso*‐nitrogen BODIPY derivatives. The syntheses and applications of azaDIPYs and aza‐BODIPYs have been the subject of recent reviews.^[^ ^8^, ^16^ ^]^

**Scheme 1:**
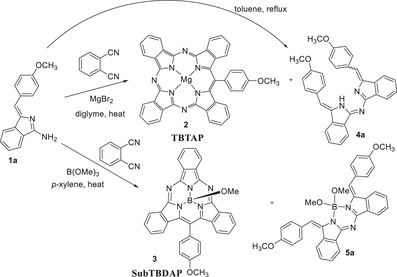
Synthesis of TBTAPs **2** and subTBDAPs **3** from aminoisoindoline precursors **1**, producing (aza)dibenzo‐DIPY **4** and BODIPY **5** derivatives as side products, and the direct self‐condensation route.

In our efforts to expand the chemistry of the porphyrin‐phthalocyanine hybrids (phthalocyanine analogues where one or more *meso*‐nitrogens are replaced by methine bridges) we have developed and refined a versatile synthesis of *meso*‐aryl‐triazatetrabenzoporphyrins (TBTAPs, e.g. **2**) employing aminoisoindoline precursors **1**.^[^ ^17^ ^]^ In reactions with phthalonitriles, templated by magnesium ions, TBTAP is formed alongside the self‐condensation products **4** from aminoisoindoline **1**. Related reactions, employing a boron template, lead to subdiazatribenzoporphyrins (e.g. **3**) alongside borylated self‐condensation dimers like **5**.^[^ ^18^ ^]^ They are, respectively, related to DIPY and BODIPY cores but with the subtle difference in their electronic structure that preserves the benzenoid aromaticity of the fused rings. An initial investigation demonstrated that direct self‐condensation smoothly yielded the DIPY analogues.^[^ ^19^ ^]^ (Scheme 1)

## 2 Results and Discussion

**Figure 3:**
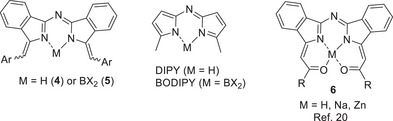
Comparison of the electronic structure of (aza)dibenzoDIPY and BODIPY with the parent systems **4** and **5**, plus related structures **6** reported by Zatsikha and Nemykin.

As mentioned previously, derivatives like **4** share a structural framework that closely resembles traditional DIPY and BODIPY materials, but the electronic arrangement is distinctly different. Zatsikha and Nemykin have also reported a related core structure **6**, constructed through enolate attack on phthalonitrile precursors followed by dimerization (Figure 3).^[^ ^20^ ^]^ We aimed to investigate the chemistry of the new motif **4**, exploring the scope for modification of the parent systems and the potential for introduction of differential substitution (e.g. push‐pull systems).

### 2.1 Synthesis of Aminoisoindoline Precursors

Aryl‐substitued aminoisoindoline precursors are readily prepared following the route originally reported by Hellal and Cuny,^[^ ^21^ ^]^ with modification of the arylacetylene reactant. The selection of arylacetylene was governed by chemistry that we wished to interrogate in the resulting DIPY analogues. Simple *p*‐methoxyphenyl substituent (**1a**) provided an electron rich aromatic terminus and the corresponding *p*‐pentyloxyphenyl and *p*‐hexyloxyphenyl derivatives (**1b**, **1c**) have the same electronic character but the longer chains allow for simple configuration information and an easy mass spectrometry marker. In contrast, *p*‐nitro‐ and/or *p*‐cyanophenyl substituents would provide an electron withdrawing terminus. Thiophene provides an alternative electron rich component, and 3‐methoxyphenyl is essentially electronically neutral, but the motivation for selecting them was more driven by the potential for further functionalization, specifically the potential to oxidatively cyclize and form porphyrin‐like macrocycles (see later).

**Scheme 2:**
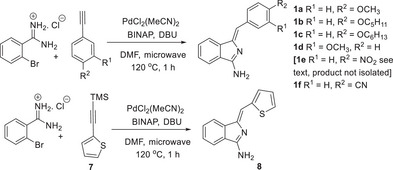
Synthesis of aminoisoindoline precursors.

**Scheme 3:**
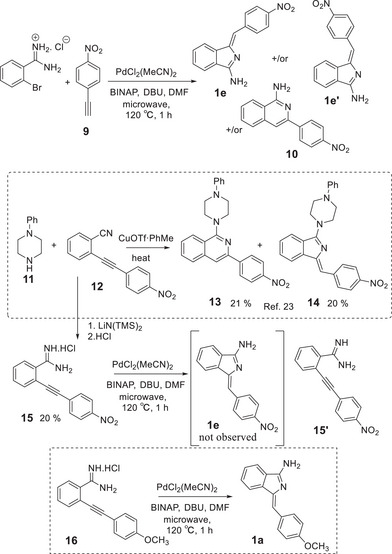
Attempted synthesis of aminoisoindoline derivative **1e**.

The synthesis of aminoisoindoline derivatives bearing alkoxyphenyl (**1a–d**) and thiophene (**8**) proceeded smoothly (Scheme 2). In the case of thiophene, reaction of 2‐ethynylthiophene itself with 2‐bromobenzamidine^[^ ^22^ ^]^ was inefficient due to dominant homocoupling of the acetylene under the reaction conditions. The reaction was much more efficient when the TMS‐protected acetylene **7** was employed directly. The synthesis of the *p*‐cyanophenyl aminoisoindoline **1f** was similarly straightforward, but the related *p*‐nitrophenyl derivative **1e** could not be isolated pure and unambiguously characterized. Here, direct reaction between *p*‐nitrophenylacetylene **9** and 2‐bromobenzamidine gave a complicated mixture from which a fraction could be isolated that displayed the correct mass in MALDI‐MS. Nevertheless, this fraction clearly contained two isomeric compounds, possibly in equilibrium, and attempts to crystallize and derivatize (tosylation) were unsuccessful. It is likely the mixture contains either E/Z isomers of the aminoisoindoline or a mixture with the aminoisoquinoline. Formation of the latter mixture has been observed in corresponding reactions of 2‐(*p*‐nitrophenylethynyl)benzonitrile with amines under catalysis.^[^ ^23^ ^]^ We attempted stepwise synthesis from this same intermediate but again a complicated mixture was produced with no evidence for the required aminoisoindoline (Scheme 3). The corresponding reaction with the 4‐methoxyphenyl acetylene **16** proceeded smoothly to give aminoisoindoline **1a**, proving that it is the electron withdrawing nitro substituent that severely affects reactivity.

**Figure 4:**
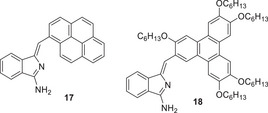
Aminoisoindoline derivatives bearing π‐extended pyrene **17** and substituted triphenylene **18**.

Two examples of π‐extended systems were also selected, appending respectively, a pyrene (**17**) and a substituted triphenylene (**18**) (Figure 4). The former represents a fluorophoric aromatic component and the latter component promotes liquid crystal self‐assembly. In both cases synthesis was straightforward from the corresponding aryl acetylenes.^[^ ^24^, ^25^ ^]^

**Scheme 4:**
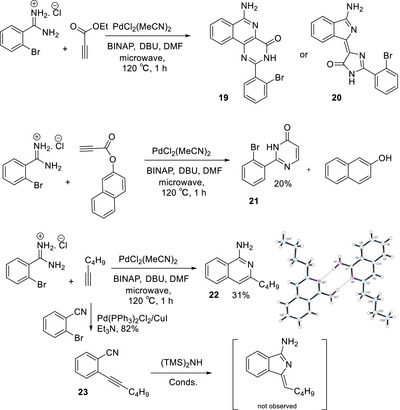
Attempted synthesis of aminoisoindolines from propargyl esters and alkyl acetylenes.

Derivatives bearing alternative terminal functionality were also briefly investigated. It is noteworthy that conjugated ester termini (related to the ketone derivatives, shown in figure 3, studied by Nemykin^[^ ^20^ ^]^) were not formed by reaction of corresponding propargylic esters with bromobenzamidine. The reactions with ethyl propiolate were dominated by acetylene homocoupling and produced a complex mixture of other products in trace quantities. One side‐product was isolated and shown to derive from reaction of two equivalents of bromobenzamide with one acetylene. Its structure is tentatively assigned as **19** but the alternative, **20**, cannot be ruled out. The naphthyl ester, selected to allow easier monitoring of reactions, similarly failed to produce the corresponding aminoisindoline product. In this case pyrimidone **21**, formed by direct reaction of the amidine with propargyl ester,^[^ ^26^ ^]^ was isolated (20%) alongside naphthol (46%). Simple alkyl acetylenes also failed to produce the corresponding aminoisoindolines. Direct reaction between 2‐bromobenzamide and 1‐hexyne under the previously employed palladium catalysis conditions produced the isomeric isoquinoline derivative **22** as the only identifiable new aromatic product. In this case the structure of the product was unequivocally assigned as the isoquinoline by crystallography. A number of reports describe conditions to favor formation of isoindolines over isoquinolines, typically employing secondary amines and alkynyl benzonitriles.^[^ ^23^, ^27^ ^]^ Attempts to apply these conditions, plus the palladium catalysis conditions employed from the preformed amidine, to the reaction between hexynyl benzonitrile **23** and hexamethyldisilazane failed to give any reaction (Scheme 4).

### 2.2 Symmetrical Diphenyl Aza‐Dibenzodipyrromethenes (Aza‐DBDPMs) and Aza‐DibenzoBODIPYs (Aza‐DBBODIPYs)

**Scheme 5:**
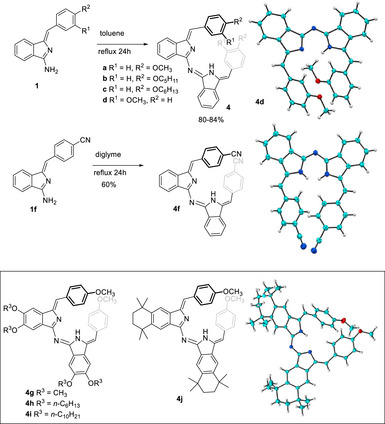
Synthesis of simple Aza‐DBDPMs bearing electron rich, neutral, and poor phenyl termini, the preferred Z, Z‐stereochemistry with helical configuration, and examples of benzo‐substituted derivatives.

In general, the condensation of aminoisoindolines **1a–d** could be easily achieved in high yield following our previously reported protocol, simply by heating in refluxing toluene for 24 hours. The electron‐poor cyanophenyl aminoisoindoline **1f** dimerized much more slowly, and efficient self‐condensation was only achieved by changing to refluxing diglyme. Benzo‐substituted derivatives reacted equally smoothly. In all cases, the Aza‐DBDPMs were isolated as red solids and NMR spectroscopy revealed all derivatives to be almost exclusively single isomers. Like **4a**, Z, Z‐stereochemistry was confirmed for **4b** and **4d** by X‐ray crystallography, revealing a helical structure no doubt stabilized by π−π interactions between the terminal aryl fragments (Scheme 5).

**Scheme 6:**
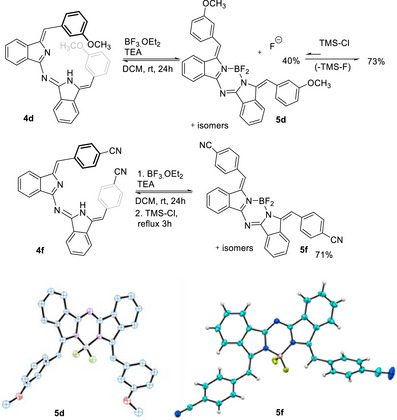
Improved synthesis of boron complexes (aza‐DBBODIPYs) by addition of TMS‐Cl to sequester fluoride ions.

Complexation with boron can be achieved using conditions analogous to those employed widely in traditional BODIPY synthesis, namely by simply treating the parent aza‐DBDPMs with boron trifluoride. The reactions, however, produce only moderate yields and were consequently investigated in more detail, initially employing aza‐DBDPM **1d** as starting material. Aza‐DBDPM **1d** was stirred in DCM and either DBU or TEA added as base. BF_3_.OEt_2_ was added, and the reaction monitored. After 24 hours the reaction contained a mixture of starting material and borylated product. Addition of excess BF_3_.OEt_2_, longer reaction times, and elevated temperature gave no improvement to the product distribution, and isolated yields of only 40% were achieved. The experimental observations indicated that the reaction was in equilibrium, and this was proved in a separate investigation. The isolated aza‐DBBODIPY **5d** was redissolved in DCM and monitored. When isolated by crystallization **5d** is obtained as a single stereoisomer (E, E) which slowly equilibrates in solution giving isomers. The isomerizing components are stable and retain boron. However, addition of TBAF hydrate induces full deboronation in minutes, confirming equilibration in the original reaction. With this information in hand, we reasoned that efficient formation of BF_2_ complexes (BODIPYs) would be possible if the generated fluoride ions were removed as formed, and this strategy proved successful. Aza‐DBDPM **4d** and BF_3_.OEt_2_ were allowed to react again for 24 hours until no further change was observed. TMS‐Cl was added and, after a further 8 hours, aza‐DBDPM **4d** appeared to be fully consumed. Aza‐DBBODIPY **5d** was isolated in an improved 73% yield. Subsequently it was found that the reaction could be completed in 3 hours at gentle reflux, and these conditions were adopted as standard for all subsequent syntheses of aza‐DBDPM‐BF_2_ (aza‐DBBODIPY) complexes (Scheme 6).

**Scheme 7:**
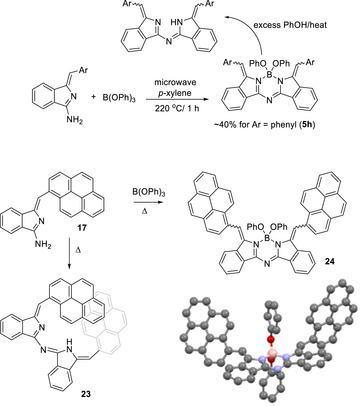
Alternative direct synthesis of boron complexes using triphenyl borate.

Alternative boron sources, such as boronate esters, can also be employed in complex formation, and indeed the synthesis can be achieved in a single step directly from the aminoisoindoline monomers. Scheme 7 shows an example using triphenyl borate. While a moderate yield is achieved for simple phenyl substituents, the yield is somewhat lower for the large pyrenyl derivative. Here again the complexation reaction is reversible under the reaction conditions, and addition of excess phenol displaces boron from the aza‐DBDPM ligand. Scheme 7 also shows the crystal structure of the dipyrenyl aza‐DBBODIPY analogue. The molecule crystallizes as the E,E‐isomer but re‐establishes an equilibrium mixture of stereoisomers (and likely atropisomers) in solution (NMR, leading to complex spectra). It can be seen, however, that such complexes offer an attractive fixed spatial arrangement of pairwise chromphores/lumophores with combination of weak (between terminal aryl fragments), and no (B─O─Aryl to termini and B─O─Aryl to B─O─Aryl) through‐bond electronic communication pathways. As expected, helical, π‐stacked aza‐DBDPM **23** shows no appreciable fluorescence, presumably due to efficient quenching, but the complexation‐induced configuration change (**24**) switches on core‐based emission, likely by a combination of planarizing the core, through the complexation to boron, and the alkene isomerization that separates the terminal perylenes (Supplementary Information).

### 2.3 Attempts to Prepare Porphyrinoid Macrocycles from Aza‐DBDPMs

**Scheme 8:**
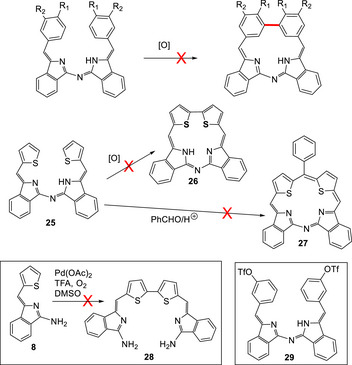
Failed attempts to produce macrocyclic products from aza‐DBDPM intermediates.

The general aza‐DBDPM structure appears, at least in theory, to be an excellent candidate for macrocyclization to prepare porphyrinoid structures. This is particularly true for the thiophenyl analogue **25**, itself easily prepared from aminoisoindoline **8**. Here, direct oxidative coupling between the terminal thiophenes would yield (dithia‐dibenzo)‐aza‐corrole **26**, while coupling via a methylene bridge would lead to (dithia‐dibenzo)‐aza‐porphyrins **27**. In practice, however, none of the investigated strategies for macrocyclization were successful. Oxidative coupling reactions on 3‐ and 4‐methoxyphenyl aza‐DBDPMs (**4d** and **4a**) and thiophenyl aza‐DBDPM **8** failed to show any evidence for macrocyclization. In metal‐based processes, such as the palladium acetate mediated reaction,^[^ ^28^ ^]^ formation of new products was clearly observed (tlc) but more careful analysis after workup revealed them to be stereoisomers that reequilibrated back to the preferred Z, Z‐isomer when separated from palladium catalyst; like boron, metal complexation leads to preference for E‐stereochemistry. The corresponding 3,3’‐ditriflate **29** was separately prepared for direct (Pd or Ni^[^ ^29^ ^]^) homocoupling (see Supporting Information) but, unsurprisingly given that the metal catalysts lead to isomers that cannot cyclize, the reaction showed no evidence for macrocyclization. Qualitatively, in all reactions, the starting material remained unconsumed, so oligomerization does not appear to occur significantly. This indicates that the catalyst is rendered inert through complexation to the aza‐DBDPM ligand (Scheme 8).

Cyclization under typical porphyrin synthesis conditions (benzaldehyde + acid) also failed, returning unreacted starting material **25**. This observation also indicated that the terminal aryl fragments are electron deficient and likely unreactive toward oxidative coupling and electrophiles. This was supported by the failure of dithiophene derivative **25** to react under standard bromination conditions, and the failure of thiophenyl aminoisoindoline **8** to form dimer **28** under standard thiophene oxidative coupling conditions (Scheme 8).^[^ ^28^, ^30^ ^]^

### 2.4 Unsymmetrical Aza‐DBDPM Derivatives

**Scheme 9:**
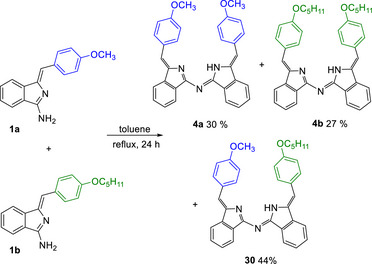
Mixed condensation/dimerization to give unsymmetrical aza‐DBDPM **30**.

This family of aza‐DBDPM materials shows an intriguing and quite rare architecture that aligns terminal aryl fragments in a stacked arrangement on a helical backbone. It offers potential for charge and energy transfer, with further potential for switching through complex formation at the ligand core that leads to stereochemical inversion of the alkenes and overall flattening. The potential of the structures would be significantly enhanced with access to unsymmetrical derivatives — systems bearing different terminal chromophores/lumophores. By far the most straightforward approach to such systems involves reacting a mixture of aminoisoindoline precursors. We started our investigation with simple systems with similar reactivity (aminoisoindolines **4a** and **4b** bearing 4‐methoxy‐ and 4‐pentyloxy‐substituents). In practice, we find that this works well to yield an essentially statistical mixture of two symmetrical products and the mixed aza‐DBDPM that can be separated relatively easily by chromatography (Scheme 9).

**Scheme 10:**
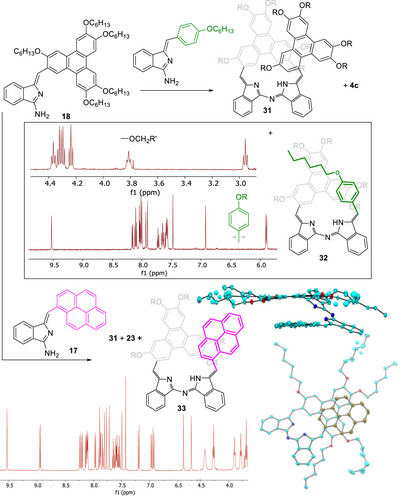
Unsymmetrical aza‐DBDPMs bearing π‐extended chromophore termini;^1^HNMR spectra of **32** and **33** showing significant influence of the stacked systems on aromatic and ─O─CH_2_─ regions, and the X‐ray crystal structure for **32**.

This straightforward approach was used to successfully prepare a range of unsymmetrical derivatives bearing one or two of the π‐extended triphenylene or pyrene systems. We found that the symmetrical self‐condensation product **31** formed from triphenylene aminoisoindoline **18** could be isolated in pure form but NMR spectra could not be recorded, presumably due to strong aggregation even in dilute solution at elevated temperature. Unsymmetrical derivatives, however, displayed well‐resolved NMR spectra and significant shifts are observed for protons affected by the neighboring (stacked) aromatic fragment that results from the helical arrangement. Scheme 10 shows the unsymmetrical products from mixed condensation reactions between triphenylene aminoisoindoline **18** and, respectively, aminoisoindoline **1c** bearing a terminal 4‐hexyloxyphenyl group (giving unsymmetrical aza‐DBDPM **32**), and aminoisoindoline **17** bearing a terminal pyrene (giving unsymmetrical aza‐DBDPM **33**).

The helical/stacked structure in aza‐DBDPM **32** places the hexyloxyphenyl terminus over the triphenylene and each component experiences the ring‐current of its neighbor. This is particularly apparent for selected protons on the aromatic cores, such as the doublet signal for one pair of protons on the hexyloxyphenyl residue which is significantly shifted upfield to ∼5.9 ppm, and the chain methylene protons closest to the cores (O─CH_2_) where one signal appears below 3.0 ppm. The unsymmetrical triphenylene‐pyrene aza‐DBDPM **33** similarly displays ^1^H NMR signals that reflect the stacked arrangement, and here we were also able to obtain crystals of suitable quality for X‐ray crystallography that show how the two terminal aromatic fragments lie in a slipped‐face‐to‐face arrangement with ∼3.5Å separation.

### 2.5 Controlled Synthesis of Unsymmetrical Aza‐DBDPM Derivatives

**Scheme 11:**
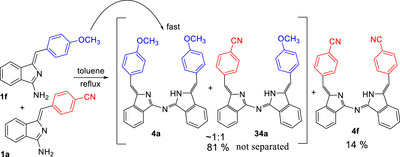
Mixed condensation between aminoisoindolines bearing electron withdrawing and donating groups fails to give good conversion to unsymmetrical dimeric product **34** due to rapid homocondensation of the electron rich partner **1a**.

The simple mixed condensation reaction between different aminoisoindolines is a convenient approach to access unsymmetrically substituted aza‐DBDPMs (as mixtures with homocondensation dimers). Ratios of products are essentially statistical when electronically similar coreactants are employed. In the reaction between two aminoisoindolines, one component presumably acts as nucleophile (at the exocyclic nitrogen) and the second acts as electrophile at the nitrogen‐bonded carbon. We initially expected that unsymmetrical products would be favored (over homocondensation) if complementary partners bearing electron donating and withdrawing aryls were reacted. We reasoned that they would respectively, enhance nucleophilicity and electrophilicity, and lead to aza‐DBDPMs with donor‐acceptor pairs at the termini. This was tested using aminoisoindolines **1a** (bearing an electron‐rich 4‐methoxyphenyl) and **1f** (bearing an electron‐poor 4‐cyanophenyl). In fact, the reaction, shown in Scheme 11, does not favor formation of the unsymmetrical product **34** and the electron rich partner **1a** preferentially reacts as both nucleophile and electrophile. Consequently, in the reaction, the homocondensation of **1a** proceeds rapidly and consumes **1a**. As its concentration becomes depleted, reaction with **1f** becomes competitive and, finally, the remaining low‐reactivity **1f** condenses with itself to form **4f**.

**Scheme 12:**
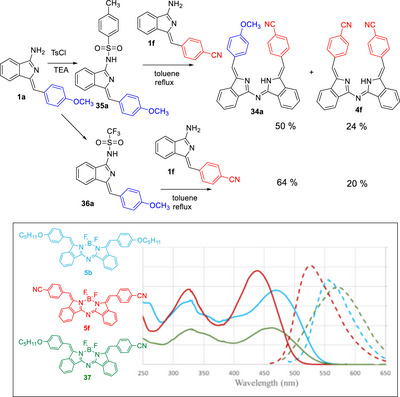
Controlled synthesis of unsymmetrical aza‐DBDPMs through tosylation/triflation of the more reactive partner. Inset: absorption (solid lines) and emission (dotted lines) spectra for a series of borate derivatives.

An alternative strategy was therefore required to control the dimer formation, and this was achieved by functionalizing the exocyclic amine nitrogen as its tosylate, thereby increasing its ability to act as a leaving group and, more importantly, removing its nucleophilicity. Our earlier investigations showed that **1a** was significantly more reactive than **1f**, so it was the obvious choice for functionalization. Tosylation of aminoisoindoline **1a** (and the longer chain homologue **1b**) proceeded smoothly using Tosyl chloride/triethylamine to give **35a** and **35b**. Tosylation on the exocyclic nitrogen was verified by crystallography (Supplementary Information). Pleasingly, tosylate **35a** remained unreacted when subjected to typical aza‐DBDPM formation conditions (reflux in toluene). Reaction with the electron‐poor cyanophenyl aminoisoindoline **1f** then successfully demonstrated the strategy, producing the unsymmetrical aza‐DBDPM **34a** as the dominant product. A small amount of homocondensation product **4f** was also isolated alongside unreacted starting materials, but again there was no homocondensation product originating from the tosylate partner (Scheme 12). Switching from tosylate to triflate improved the selectivity further.

While the approach was designed for cases where simple mixed condensation failed, it is worth noting that it is also effective for improving yields and reducing the product complexity in reactions that involve partners with similar reactivity. Here, tosylation or triflation of one partner again prevents its homocondensation and the unsymmetrical aza‐DBDPM dominates the product mixture. However, the yield of homocondensation product from the nonfunctionalised aminoisoindoline is also significant and this implies that the selectivity is driven by reduction of the nitrogen nucleophilicity rather than enhancing leaving group ability through functionalization with tosylate/triflate (Supplementary Information). Conversion to the corresponding difluoroboron adducts was smoothly achieved following the protocol previously described, allowing preliminary comparison of absorption and emission spectra across the series (Scheme 12, inset). Symmetrical dicyano aza‐DBBODIPY **5f** absorbs at 441 nm (emission at 527 nm). Interchange of one cyanide with alkoxide red‐shifts the absorption of this unsymmetrical derivative **37** to 465 nm and emission to 567 nm (largest Stokes shift in the series at 0.48 eV and significantly larger than typically observed in BODIPYs^[^ ^31^ ^]^). Symmetrical dialkoxide **5b** has similar properties, absorbing at 475 nm and emitting at 561 nm.

## 3 Conclusion

The class of aza‐dibenzodipyrromethenes reported in this study presents an interesting architecture where the core adopts a helical arrangement that places terminal aryl functional groups directly on top of each other in close, π‐stacked arrangement. As with the related synthesis of BODIPY systems, complexation with boron is readily achieved through reaction with borate esters or boron trifluoride. The reactions are shown to be reversible, and conversions are significantly improved through addition of TMS‐Cl to sequester fluoride ions (in the case of BF_3_ reactions). Complexation induces a stereochemical inversion, flattens the system, and switches on fluorescence. Synthesis of the systems is straightforward, and a wide range of terminal aromatic fragments can be easily introduced. Unsymmetrical derivatives can be made conveniently by simple mixed condensations, but a controlled, rational approach is also described whereby the ability of one component to homocondense (the more reactive partner) is removed by conversion to its corresponding tosylate or triflate. The two approaches have been investigated through successful introduction of complementary electron rich, electron poor, and π‐extended functional termini, and the versatility of this unusual molecular scaffold has been demonstrated.

## Supporting Information

The authors have cited additional references within the Supporting Information.^[^ ^32^, ^33^, ^34^ ^]^ Deposition Number(s) <url href = “https://www.ccdc.cam.ac.uk/services/structures?id=https://doi.org/10.1002/chem.202501862”>2442882 (for **4d**), 2442876 (for **4f**), 2442885 (for **4j**), 2442878 (for **5b**), 2442883 (for **5d**), 2442879 (for **5f**), 2442880 (for **8**), 2442874 (for **21**), 2442877 (for **22**), 2442737 (for **24**), 2442881 (for **25**), 2442884 (for **33**), 2442875 (for **36a**), </url> contain(s) the supplementary crystallographic data for this paper. These data are provided free of charge by the joint Cambridge Crystallographic Data Centre and Fachinformationszentrum Karlsruhe <url href = “http://www.ccdc.cam.ac.uk/structures”>Access Structures service</url>.

## Conflict of Interest

The authors declare no conflict of interest.

## Supporting information



Supporting Information

## Data Availability

The data that support the findings of this study are available from the corresponding author upon reasonable request.
