# Supplementary material for: A Family of Symmetrical and Unsymmetrical Aza‐Dipyrromethenes and Aza‐BODIPYs with Through‐Bond and Face‐To‐Face π‐Interaction between Termini
Source: Chemistry. 2025 Jul 14;31(50):e202501862. doi: 10.1002/chem.202501862 (PMC12415328; doi:10.1002/chem.202501862)
Supplement: Supplementary file 1 — Supporting Information [file CHEM-31-e202501862-s001.pdf]

A family of Symmetrical and Unsymmetrical Aza-Dipyrromethenes and Aza-BODIPYs with Through-Bond and Face-to-Face π-Interaction Between Termini

Budur N. Alanazi,† Ahad O. Alsahli,† Sonia Remiro-Buenamañana, † Alejandro Díaz-Moscoso,† Faeza H. Alkorbi,† Norah A. Alsaiari,† Conor Marrett-Munro,† Isabelle Chambrier,† David L. Hughes,† Simon J. Coles,‡ Graham J. Tizzard‡ and Andrew N. Cammidge*†

†School of Chemistry, Pharmacy and Pharmacology, University of East Anglia, Norwich Research Park, Norwich NR4 7TJ, UK

‡UK National Crystallography Service, School of Chemistry, University of Southampton, Southampton SO17 1BJ, UK

Supporting Information

Contents

Syntheses and characterisation details……………………………………..2

-Aminoisoindolines…………………………………………………………………….3

-Symmetrical aza-DBDPMs……………………………………………………..13

-Boron complexes…………………………………………………………...…25

-Unsymmetrical aza-DBDPMs…………………………………………..…33

Crystallographic details……………………………………….………....45

<u>Synthesis and characterisation details</u>

General Methods

Reagents and solvents were obtained from commercial sources and used without further purification unless otherwise stated. Phthalonitrile was recrystallised from hot xylene. THF was freshly distilled from sodium and benzophenone. Reactions and distillation were carried out under an inert atmosphere (argon or nitrogen gas), in most air-sensitive reactions argon was preferred. Brine is a saturated aqueous solution of sodium chloride. Organic layers were dried using anhydrous magnesium sulphate. Evaporating of solvent was performed using a Buchi rotary evaporator at reduced pressure. ^{1}H NMR spectra were recorded either at 400 MHz on Ultrashield Plus™ 400 spectrometer or 500 MHz on a Bruker Ascend™ 500 spectrometer in 5 mm diameter tubes. Signals are quoted in ppm as δ downfield from tetramethylsilane (δ=0.00) and coupling constants *J* given in Hertz. $^{13}C\{^{1}H\}$ spectra were recorded at 100.6 MHz or 125.7 MHz on the same spectrometers. NMR spectra were performed in solution using deuterated chloroform, methanol, dichloromethane or tetrahydrofuran at room temperature unless otherwise stated. Ultraviolet-Visible absorption spectra were recorded on Hitachi U-3310 Spectrophotometer in solvent as stated. MALDI-TOF mass spectra were carried out using a Shimadzu Biotech Axima instrument. Characterization by mass spectrometry was achieved by HRMS, performed by the ESPRC UK National Mass Spectrometry Service Centre at Swansea, and/or comparison of isotopic distribution to theory. IR spectra were recorded using a Perkin-Elmer Spectrum BX FT-IR spectrometer. Thin layer chromatography (TLC) was performed using aluminium sheets coated with Alugram® Sil G/UV254 (Macherey-Nagel), and the compounds were visualised under short-wavelength UV-light at 245 nm or 366 nm. Column chromatography was carried out using silica gel 60Å mesh 70 – 230 (63 – 200 µm) under gravity or moderate pressure at ambient temperature. Solvent ratios are given as v: v. Melting points were taken on a Reichart Thermovar microscope with a thermopar based temperature control. Reactions using microwave irradiation were carried out in Biotage Initiator+ Microwave system.

General synthesis of aminoisoindolines[21]

A mixture of 2-bromobenzamidine hydrochloride (3 mmol), BINAP (102 mg, 0.165 mmol, 0.055eq) and $PdCl_2(MeCN)_2$ (39 mg, 0.15 mmol, 0.05eq) was sealed in a microwave vessel with a magnetic bar and then purged and refilled with N_2 three times. Then, arylethyne (3.6 mmol, 1.2eq) and DBU (1.12 ml, 1.14 g, 7.5 mmol, 2.5eq) in dry DMF (12 ml) were added. The mixture was stirred under N_2 for 5 min and then irradiated in a microwave reactor at 120°C for 1 h. After cooling, ethyl acetate (50 ml) was added and the mixture washed with a saturated solution of $NaHCO_3$ (3x75 ml). The organic layer was dried with $MgSO_4$, filtered and concentrated. The residue was finally purified by column chromatography using PE:AcOEt (1:1) then AcOEt as solvent gradient to afford a yellow semisolid that was recrystallized from DCM:Petroleum ether (1:1) to yield the title compound.

(*Z*)-1-[(4-pentyloxy)benzylidene]-1*H*-isoindol-3-amine (1b)

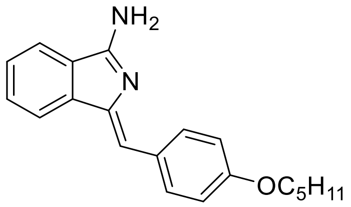

Following the general procedure for synthesis of aminoisoindolines, aminoisoindoline **1b** was isolated as yellow needles (670 mg, 73%). **Mp** 120-122°C. **^{1}H-NMR** (500 MHz, $CDCl_3$) δ 8.07 (d, *J* = 8.6 Hz, 2H), 7.77 (d, *J* = 7.1 Hz, 1H), 7.45 (dd, *J* = 8.3, 7.2 Hz, 2H), 7.35 (td, *J* = 7.2, 1.0 Hz, 1H), 6.93 (d, *J* = 8.8 Hz, 2H), 6.74 (s, 1H), 4.00 (t, *J* = 6.6 Hz, 2H), 1.83 – 1.77 (m, 2H), 1.48 – 1.36 (m, 4H), 0.94 (t, *J* = 7.1 Hz, 3H). **^{13}C NMR** (126 MHz, $CDCl_3$) δ 164.50, 158.87, 143.32, 132.11, 130.75, 129.45, 129.04, 126.86, 119.70, 118.89, 115.94, 114.74, 68.16, 29.12, 28.35, 22.63, 14.18. **MS (MALDI-TOF): *m/z* =** 306 [M^+, 100 %]. **UV-vis:** (DCM): λ_{max} (nm) (ε ($dm^3.mol^{-1}.cm^{-1}$)) = 397 ($5.6x10^4$), 377 ($8.2x10^4$), 288 ($2.4x10^4$), 230 ($6.7x10^4$).

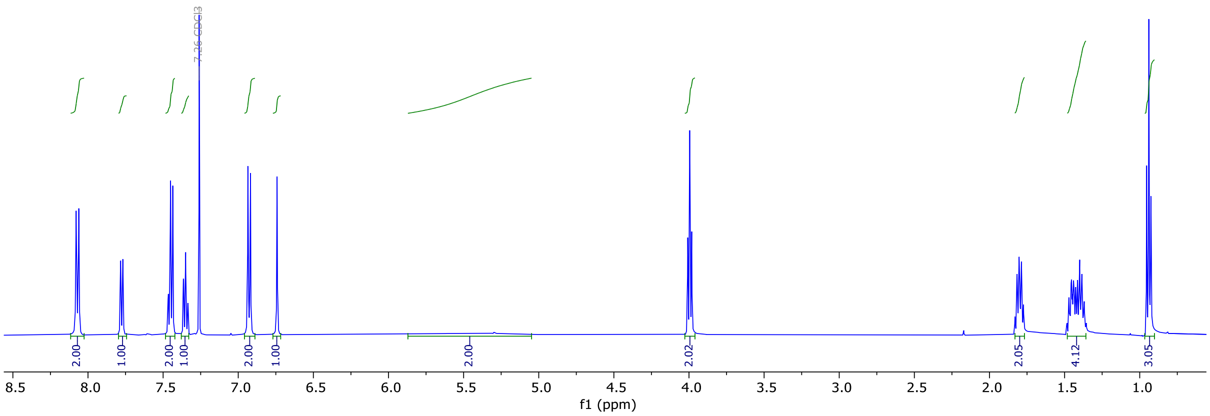

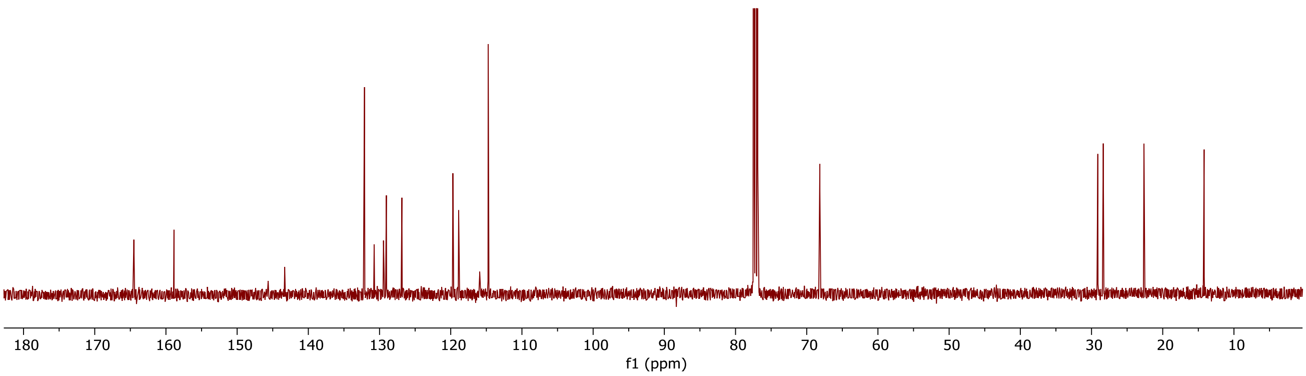

(*Z*)-1-[(4-hexyloxy)benzylidene]-1*H*-isoindol-3-amine (1c)

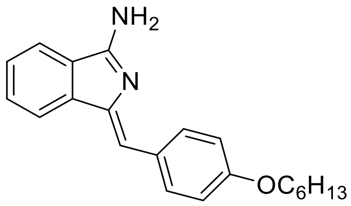

Following the general procedure for synthesis of aminoisoindolines, aminoisoindoline **1c** was isolated as a yellow solid (200 mg, 42%). **Mp** 110-112°C. **^{1}H NMR** (400 MHz, Acetone-*d*$_6$) δ 8.23 (d, J = 8.8, 2H), 7.83 (dt, J = 7.6, 1.0 Hz, 1H), 7.77 (dt, J = 7.5, 1.0 Hz, 1H), 7.43 (td, J = 7.4, 1.1 Hz, 1H), 7.35 (td, J = 7.4, 1.0 Hz, 1H), 6.90 (d, J = 8.8, 2H), 6.78 (br-s, 2H), 6.72 (s, 1H), 4.02 (t, J = 6.5 Hz, 2H), 1.82 – 1.72 (m, 2H), 1.53 – 1.43 (m, 2H), 1.43 – 1.31 (m, 4H), 0.91 (t, J = 6.8, 3H). **^{13}C NMR** (126 MHz, Chloroform-*d*) δ 164.31, 158.98, 144.69, 142.98, 134.15, 132.09, 130.35, 129.34, 129.16, 127.00, 119.73, 119.21, 115.96, 114.80, 68.18, 31.76, 29.38, 25.87, 22.76, 14.19.. **MS (MALDI-TOF): *m/z* =** 320 [M$^+$, 100 %]. **UV-vis:** (DCM): λ_{max} (nm) (ε (dm^3.mol^{-1}.cm^{-1})) = 397 (2.6 × 10^4), 378 (3.4× 10^4), 359 (2.7 × 10^4), 286 (0.8 × 10^4), 233 (0.2 × 10^5).

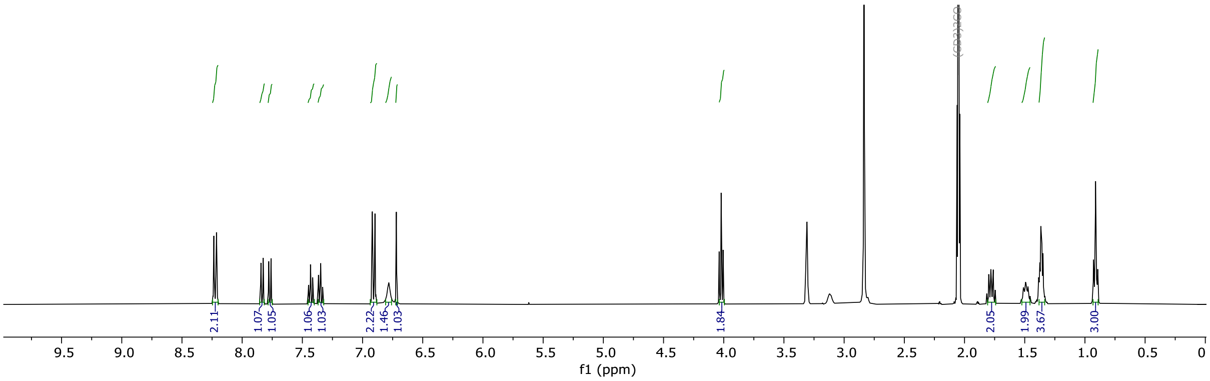

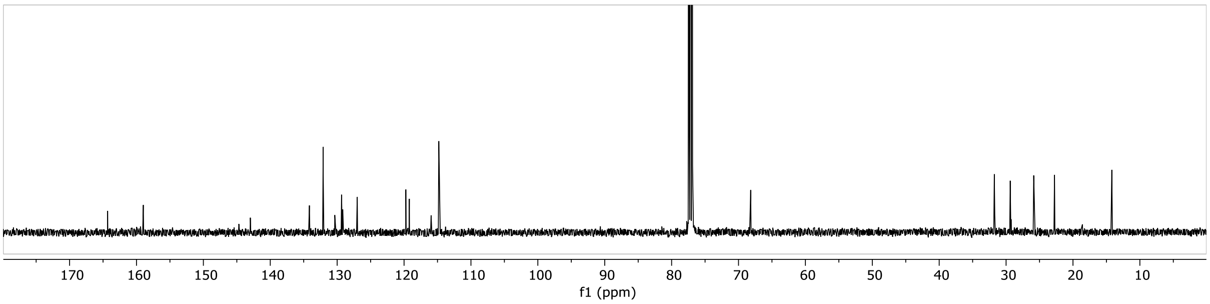

(*Z*)-1-[(3-methoxy)benzylidene]-1*H*-isoindol-3-amine (1d)

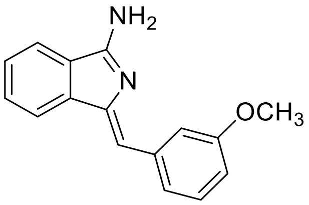

Following the general procedure for synthesis of aminoisoindolines, aminoisoindoline **1d** was isolated as a yellow crystalline solid (540 mg, 72%). **Mp** 183-184°C. **^{1}H NMR** (500 MHz, $CDCl_3$) δ 7.84 (br-t, 1H), 7.79 (dt, *J* = 7.7, 1.0 Hz, 1H), 7.57 (d, *J* = 7.5 Hz, 1H), 7.50 – 7.44 (m, 2H), 7.38 (td, *J* = 7.4, 1.0 Hz, 1H), 7.31 (t, *J* = 8.0 Hz, 1H), 6.83 (ddd, *J* = 8.1, 2.7, 0.9 Hz, 1H), 6.73 (s, 1H), 3.89 (s, 3H). **^{13}C NMR** (126 MHz, Chloroform-*d*) δ 165.21, 159.61, 147.62, 142.95,138.07, 131.11, 129.32, 129.23, 127.27, 123.25, 119.82, 118.89, 115.54, 114.85, 113.31, 55.35. **MS (MALDI-TOF): *m/z* =** 250 [M^+, 100 %]. **UV-vis:** (DCM): λ_{max} (nm) (ε ($dm^3.mol^{-1}.cm^{-1}$)) = 389 (2.6 x 10^4), 360 (5.5x10^4), 315 (2.7x10^4), 230 (6.7x10^4), 277 (2.9x10^4), 229 (6.8x10^4).

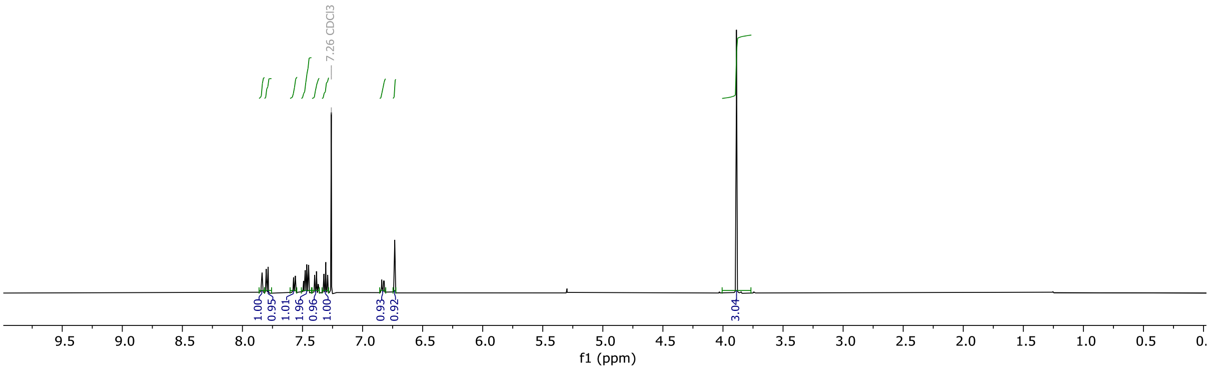

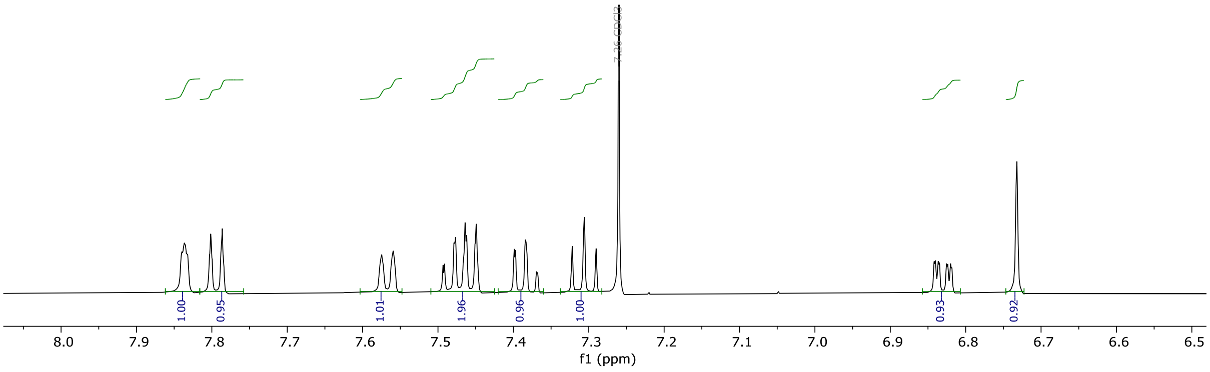

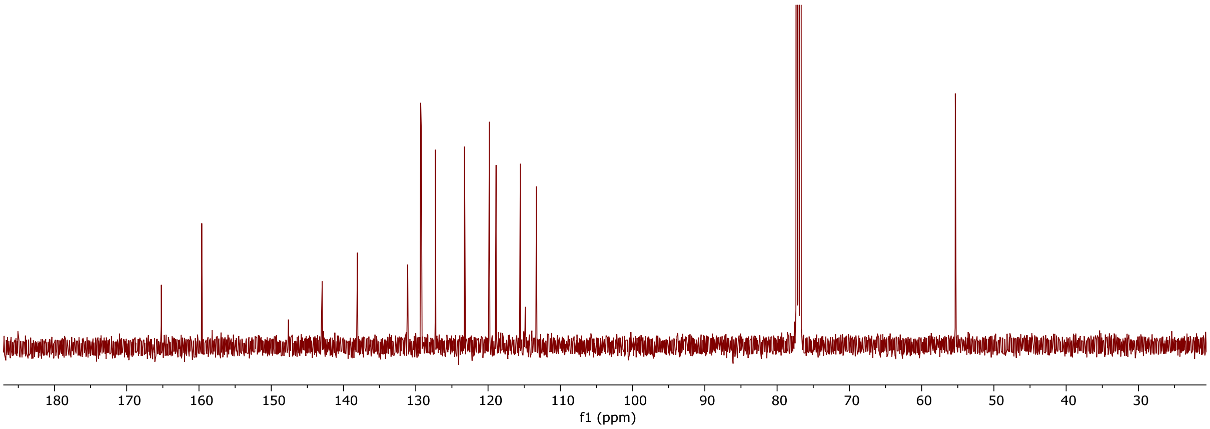

(*Z*)-1-[(4-cyano)benzylidene]-1*H*-isoindol-3-amine (1f)

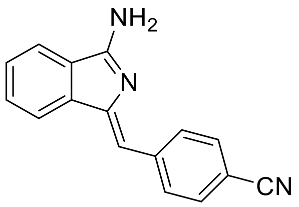

Following the general procedure for synthesis of aminoisoindolines, aminoisoindoline **1f** was isolated as a yellow powder was isolated as a yellow powder (480 mg, 65%). **Mp** 226-228°C. **^{1}H NMR** (500 MHz, Acetone-*d*$_6$) δ 8.50 (d, *J* = 9.0 Hz, 2H), 7.91 (dt, *J* = 7.6, 0.9 Hz, 1H), 7.86 (dt, *J* = 7.5, 0.9 Hz, 1H), 7.76 (d, *J* = 8.0 Hz, 2H), 7.52 (td, *J* = 7.4, 1.1 Hz, 1H), 7.46 (td, *J* = 7.4, 1.0 Hz, 1H), 7.28 (br-s, 2H-NH_2), 6.79 (s, 1H). **^{13}C NMR** (126 MHz, Acetone-*d*$_6$) δ 168.09, 153.51, 144.28, 143.61, 133.41, 132.63, 131.62, 130.26, 128.85, 121.01, 120.81, 120.06, 111.46, 109.76. **IR** (thin film cm $^{-1}$) 2220 (CN). **MS (MALDI-TOF):** ***m/z*** **=** 245 [M$^+$, 100 %]. **UV-vis:** (DCM): λ_{max} (nm) (ε (dm^3.mol^{-1}.cm^{-1})) = 406 (3.8x10^4), 379 (7.6x10^4), 327 (3.6x10^4), 291 (3.4x10^4), 232 (7.6x10^4).

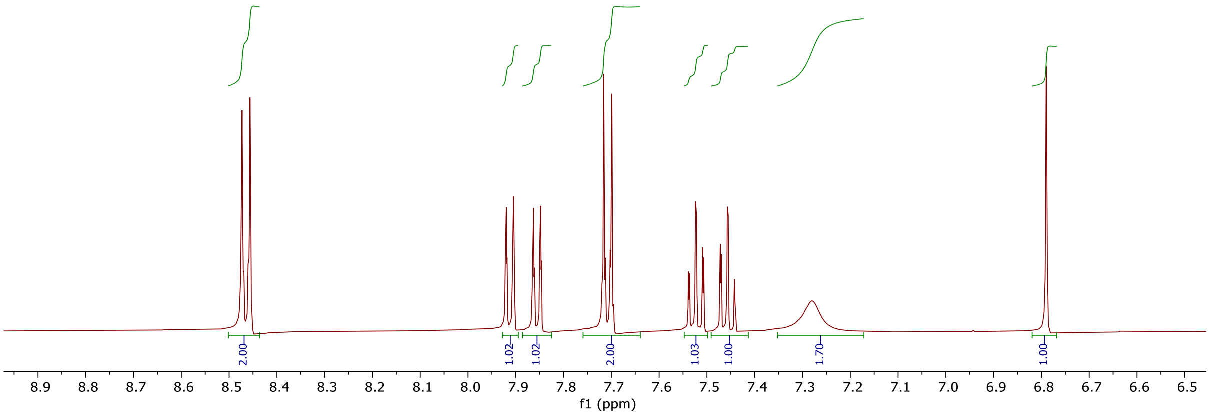

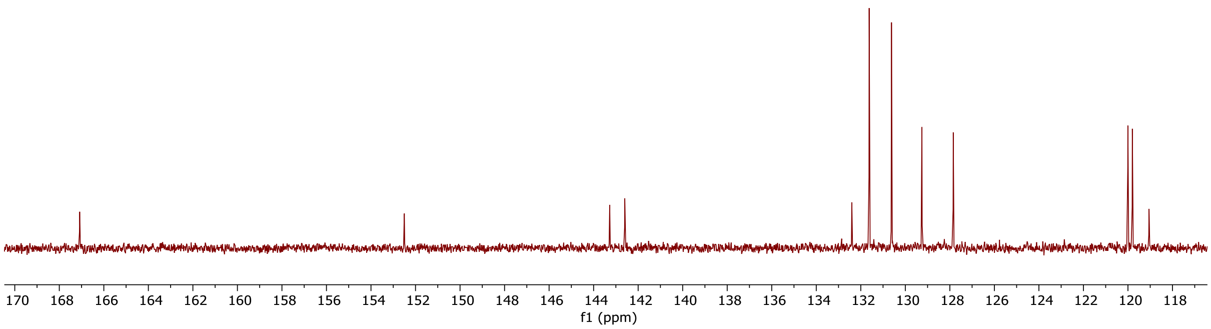

(*Z*)-1-[(3-hydroxy)benzylidene]-1*H*-isoindol-3-amine 1k

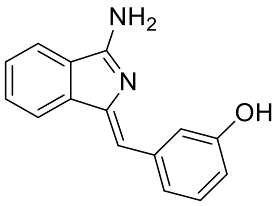

Following the general procedure for synthesis of aminoisoindolines, aminoisoindoline **1k** was isolated as a yellow powder (530 mg, 74%). **Mp** 123-125 °C. **^{1}H NMR** (500 MHz, Acetone-*d*$_6$) δ 7.92 (dd, *J* = 2.6, 0.53 Hz, 1H), 7.86 (dt, *J* = 7.7, 1.0 Hz, 1H), 7.79 (dt, *J* = 7.5, 0.9 Hz, 1H), 7.65 (dt, *J* = 1.6, 0.8 Hz, 1H), 7.46 (dd, *J* = 7.4, 1.1 Hz, 1H), 7.40 – 7.37 (m, 1H), 7.15 (t, *J* = 7.8 Hz, 1H), 6.71 (ddd, *J* = 8.0, 2.5, 1.0 Hz, 1H), 6.69 (s, 1H). **^{13}C NMR** (126 MHz, Acetone-*d*$_6$) δ 165.65, 157.12, 143.72, 138.96, 132.10, 128.73, 128.59, 126.94, 122.28, 119.59, 119.33, 117.19, 113.94, 113.45. **MS (MALDI-TOF): *m/z* =** 236 [M$^+$, 100 %]. **UV-vis:** (DCM): λ $_{max}$ (nm) (ε (dm^3.mol^{-1}.cm^{-1})) 390 (2.9x10^4), 370 (4.8x10^4), 334 (2.8x10^4), 282 (4.4x10^4), 229 (5.0x10^4).

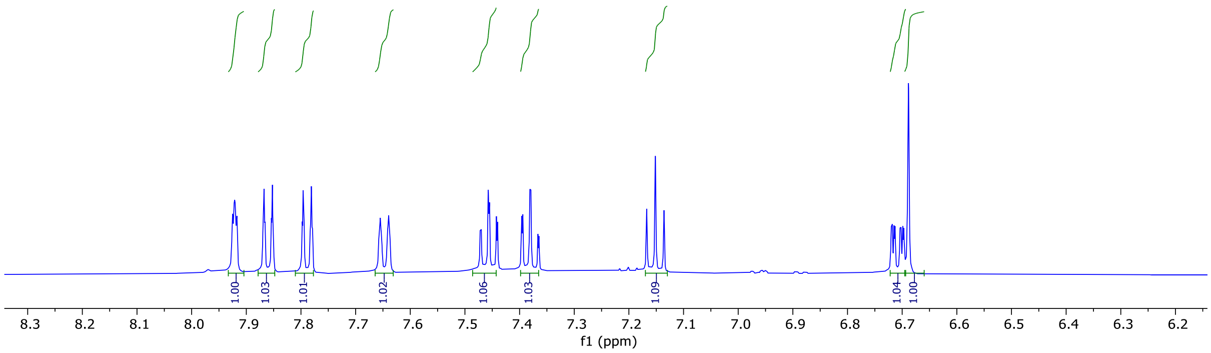

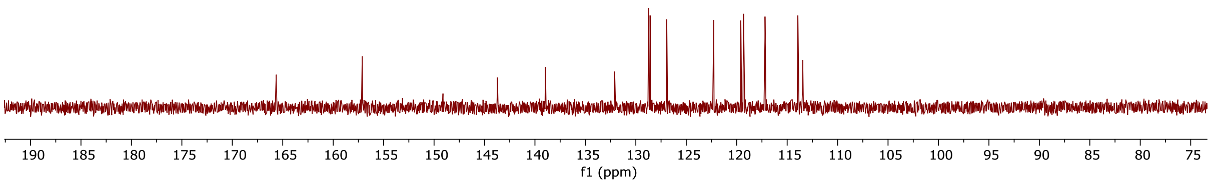

(*Z*)-1-[2-thiophenylidene]-1*H*-isoindol-3-amine (8)

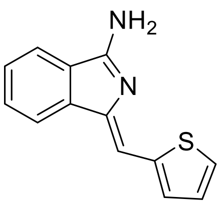

Prepared following the general procedure for synthesis of aminoisoindolines but using TMS-protected 2-ethynylthiophene **7**. Aminoisoindoline **8** was isolated as a yellow powder (510 mg, 49%). **Mp** 133-135°C. **^{1}H NMR** (500 MHz, Acetone-*d_6*) δ 7.82 (dt, *J* = 7.5, 1.0 Hz, 1H), 7.77 (dt, *J* = 7.5, 1.0 Hz, 1H), 7.45 – 7.40 (m, 2H), 7.35 (td, *J* = 7.4, 1.0 Hz, 1H), 7.31 (dt, *J* = 3.7, 0.8 Hz, 1H), 7.05 (s, 1H), 7.00 (dd, *J* = 5.2, 3.6 Hz, 1H) 6.88 (brs, 1H, NH_2). **^{13}C NMR** (126 MHz, Acetone-*d_6*) δ 164.92, 147.50, 142.82, 141.50, 133.21, 128.97, 128.60, 128.42, 127.18, 126.68, 120.21, 119.67, 108.27. **MS (MALDI-TOF):** ***m/z*** **=** 227 [M+H$^+$, 100%]. **UV-vis:** (DCM): λ_{max} (nm) (ε ($dm^3.mol^{-1}.cm^{-1}$)) = 403 (4.5x10^4), 385 (6.3x10^4), 354 (4.2x10^4), 281 (4.8x10^4), 230 (6.5x10^4).

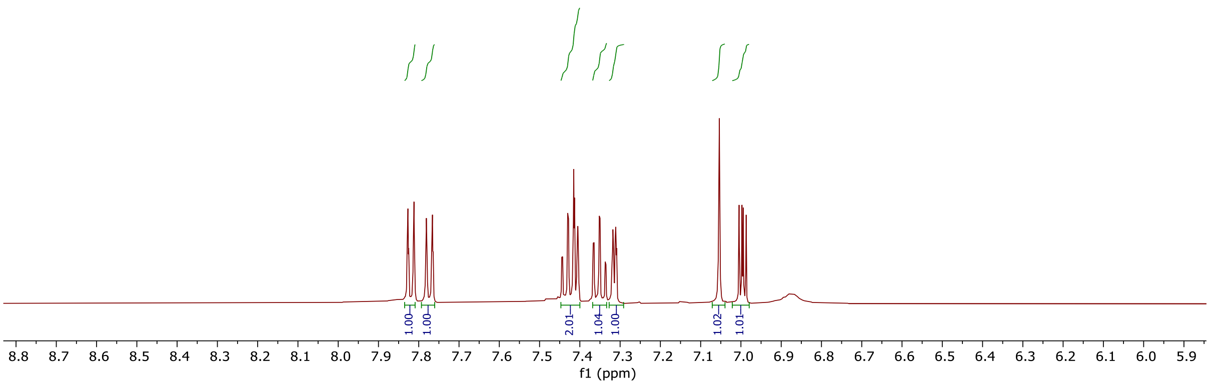

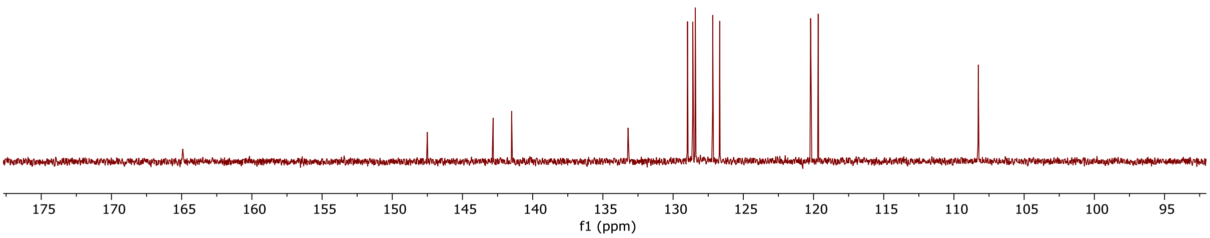

2-[2-(4-methoxyphenyl)ethynyl]-benzamidine hydrochloride 15 and (*Z*)-1-[(4-methoxy)benzylidene]-1*H*-isoindol-3-amine (1a)

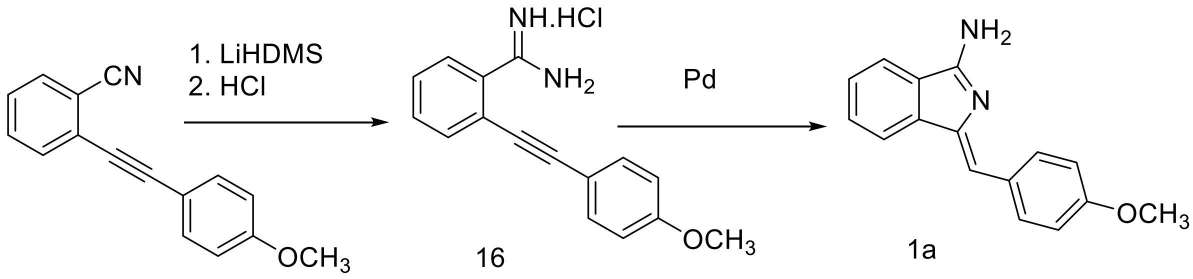

A solution of 2-[2-(4-methoxyphenyl)ethynyl] benzonitrile[27] (0.50 g, 2.14 mmol, 2 eq) in dry THF (3 ml) was stirred at room temperature. Lithium bis(trimethylsilyl) amide (1M in THF), (4.3 ml, 4.3 mmol, 2 eq) was added and stirring continued at room temperature overnight. Then the reaction was cooled down on an ice bath and quenched by adding dropwise of 8 ml of a 1:1 mixture of HCl (5N) and isopropanol, and then it was left to stir overnight. The resulting precipitate was filtered off and washed twice with diethyl ether to give the product as yellow crystals (0.4 g, 80 %). **^{1}H NMR** (500 MHz, Methanol-d_4) δ 7.75 (dd, *J* = 7.8, 1.4 Hz, 1H), 7.72 – 7.65 (m, 2H), 7.58 (td, *J* = 7.6, 1.3 Hz, 1H), 7.51 (d, *J* = 8.8 Hz, 2H), 7.00 (d, *J* = 8.8 Hz, 2H), 3.87 (s, 3H). **^{13}C NMR** (126 MHz, Methanol-d_4) δ 167.12, 160.64, 132.84, 132.53, 131.95, 131.31, 128.16, 127.93, 122.10, 114.01, 113.96, 94.70, 83.38, 54.50.

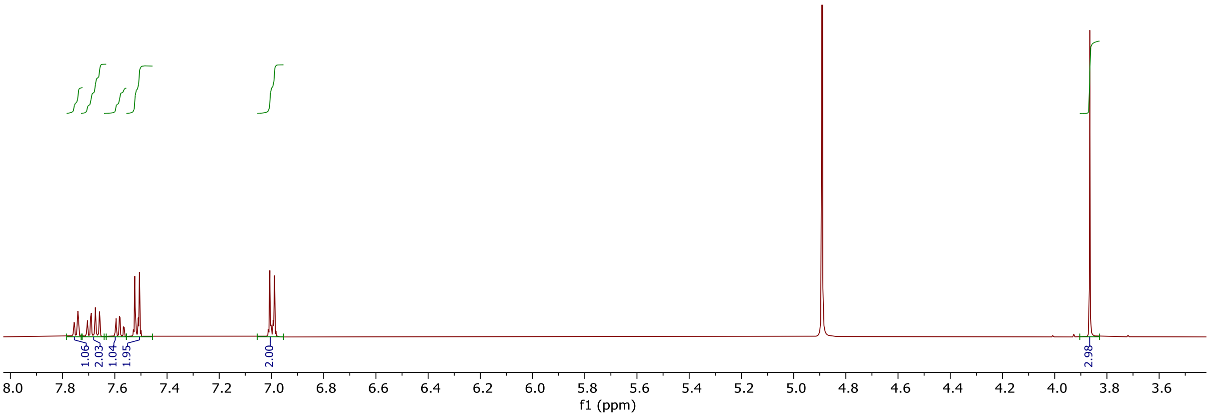

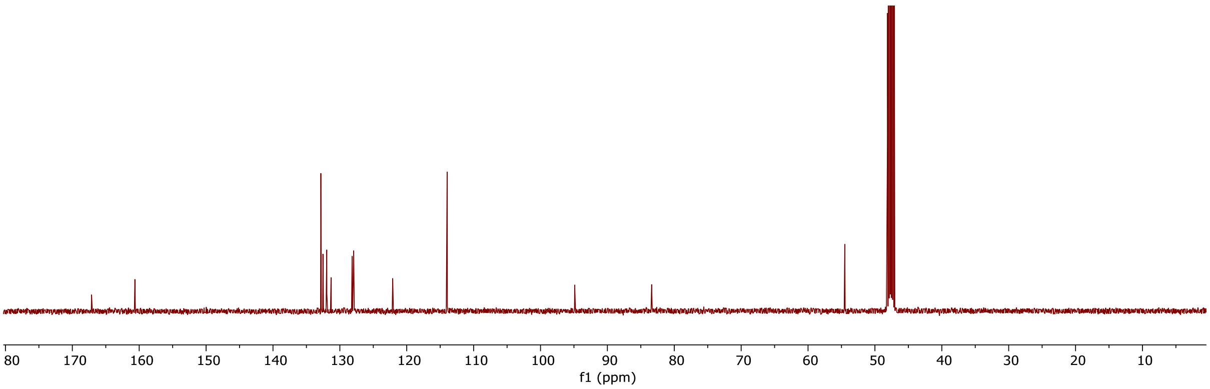

Following the general procedure for synthesis of aminoisoindolines (no added aryl acetylene), aminoisoindoline **1a** was isolated as a yellow solid (230 mg, 65%).

2-[2-(4-nitrophenyl)ethynyl]-benzamidine hydrochloride 15

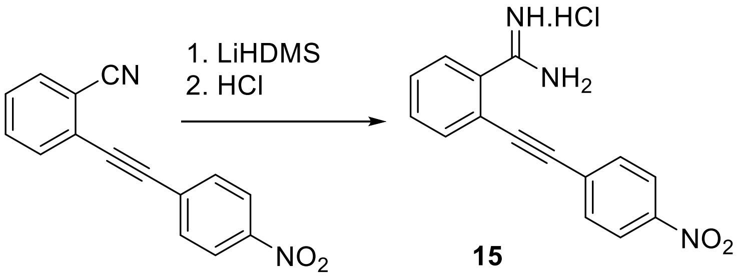

Prepared following the procedure described above for synthesis of **16** from 2-[2-(4-nitrophenyl)ethynyl] benzonitrile.[32] Amidine hydrochloride **15** was isolated as a low-solubility yellow solid (50 mg, 28%) that was used without further purification. **^{1}H NMR** (500 MHz, Methanol-d_4) δ 8.32 (d, *J* = 9.0 Hz, 2H), 7.86 – 7.81 (m, 3H), 7.78 (ddd, *J* = 7.8, 1.4, 0.7 Hz, 1H), 7.74 (dt, td, *J* = 7.8, 1.4 Hz, 1H), 7.52 (td, *J* = 7.8, 1.4 Hz, 1H).

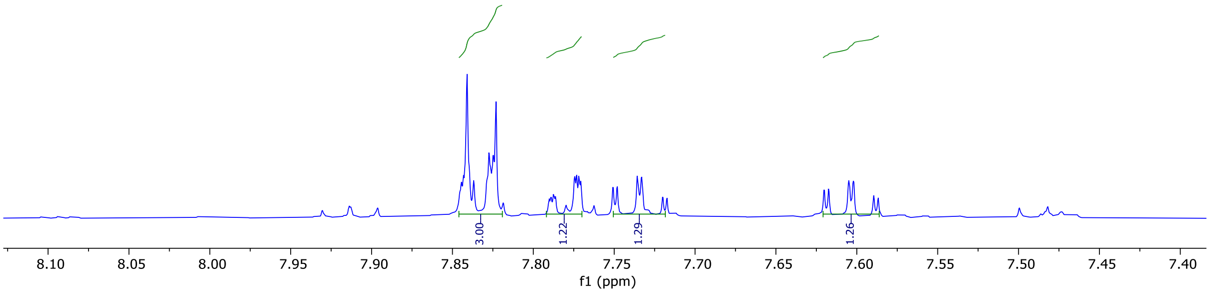

***Z*)-2-[(3, 6, 7, 10, 11-pentakishexyloxy)triphenylenidene]-1*H*-isoindol-3-amine (18)**

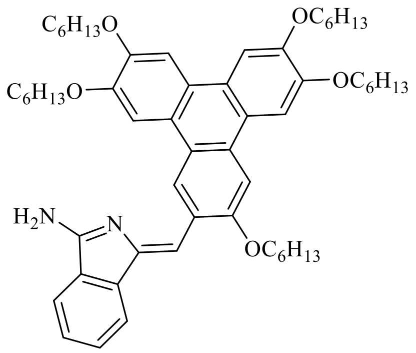

Following the general procedure for synthesis of aminoisoindolines, aminoisoindoline **18** was isolated as a yellow solid (101 mg, 21%). **Mp** 125-126 °C. **^{1}H NMR** (500 MHz, Acetone-d_6) δ 10.40 (s, 1H), 8.21 (s, 1H), 8.10 (s, 1H), 8.03 (s, 1H), 8.02 (s, 1H), 7.99 (s, 1H), 7.88 (d, *J* = 7.7 Hz, 1H), 7.84 (d, *J* = 7.5 Hz, 1H), 7.51 (td, *J* = 7.4, 1.0 Hz, 1H), 7.42 (td, *J* = 7.4, 0.9 Hz, 1H), 7.40 (s, 1H), 4.36- 4.23 (m, 10H), 1.98 – 1.85 (m, 10H), 1.68 – 1.58 (m, 10H,), 1.43 (m, 20H), 0.98 – 0.90 (m, 15H). **MS (MALDI-TOF):** ***m/z*** **=** 870.60 [M$^+$, 100 %]. **UV-vis:** (DCM): λ_{max} (nm) (ε (dm^3.mol^{-1}.cm^{-1})) = 411 (3×10^4), 438 (shoulder) (2×10^4).

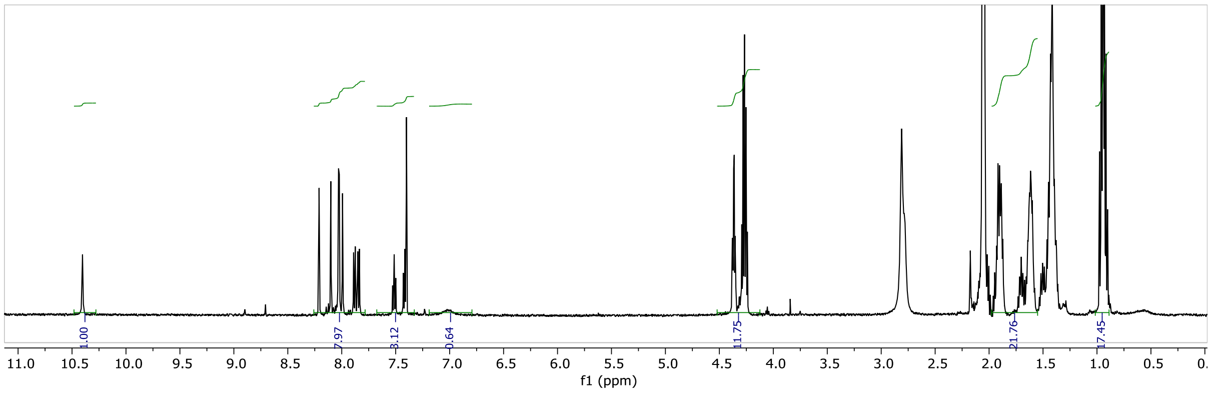

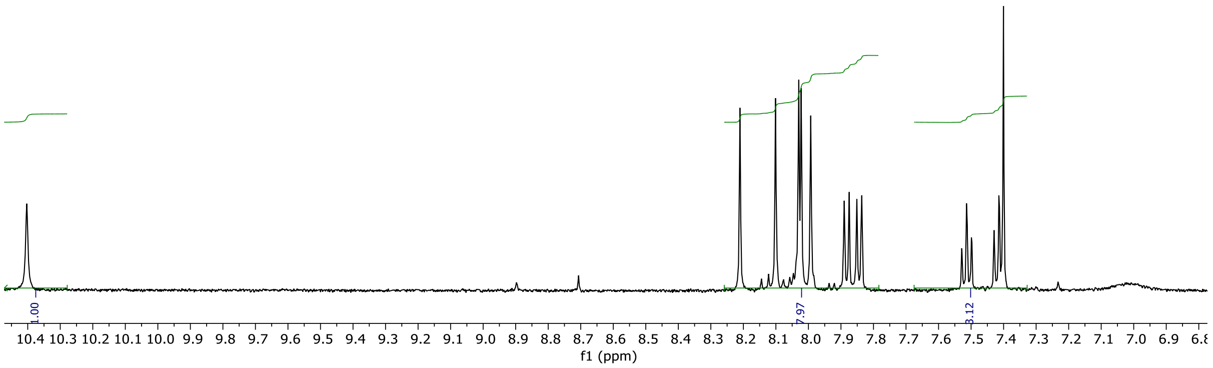

2-(2-bromophenyl)-4(3H)-pyrimidinone 21.

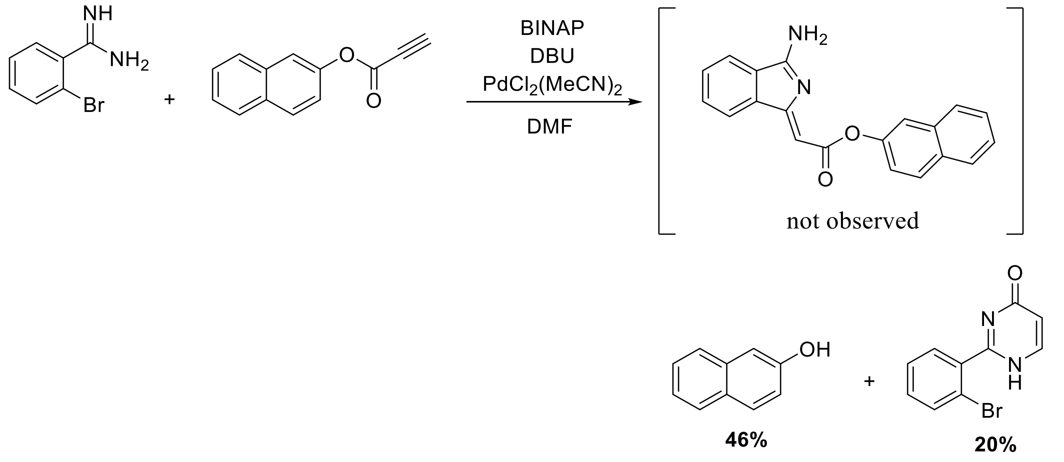

A mixture of 2-bromobenzamidine hydrochloride (0.706 g, 2.997 mmol, 1 eq), BINAP (0.102 g, 0.165 mmol, 0.055 eq) and $PdCl_2(MeCN)_2$ (0.039 g, 0.150 mmol, 0.05 eq) was sealed in a microwave vessel with magnetic bar. It was purged and refilled with nitrogen for 5 min. Then naphthyl propiolate (0.705 g, 3.596 mmol, 1.2 eq), DBU (1.120 ml) in dry DMF (12 ml) was added. The reaction was kept stirring under N_2 for a further 5 min. Then the mixture irradiated by microwave at 120 °C for 1 h. The solution was allowed to cool down, AcOEt (50 ml) added and then washed with a saturated solution of $NaHCO_3$ (3x25 ml). The organic layer was extracted and dried over $MgSO_4$, filtered, and then the solvents removed. Finally, the crude was purified by column chromatography eluting with DCM then 1:3 AcOEt: PE then AcOEt to give 2-naphthol (0.2 g, 46 %), and (2-bromophenyl)-4(3H)-pyrimidinone (0.15 g, 20 %). **Mp** 164-165 °C. **^{1}H NMR** (500 MHz, Chloroform-*d*) δ 8.08 (d, *J* = 7.0 Hz, 1H), 7.69 (ddd, *J* = 16.7, 7.8, 1.5 Hz, 2H), 7.47 (td, *J* = 7.7, 1.1 Hz, 1H), 7.40 (td, *J* = 7.7, 1.8 Hz, 1H), 6.45 (d, *J* = 7.0 Hz, 1H). **^{13}C NMR** (126 MHz, Chloroform-*d*) δ 162.89, 157.68, 154.98, 134.03, 133.93, 132.37, 131.17, 127.94, 120.74, 114.70.

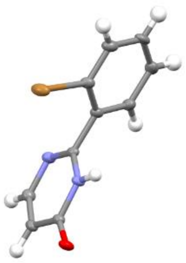

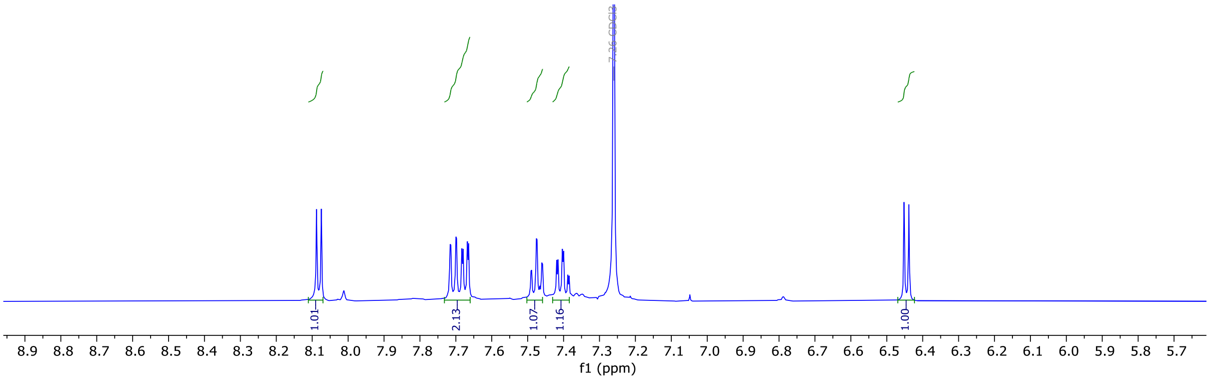

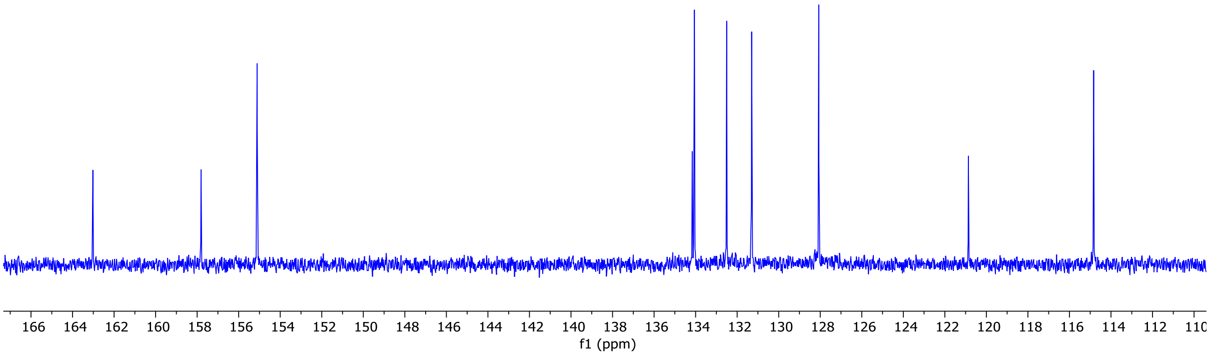

3-Butyl isoquinoline -1-amine 22

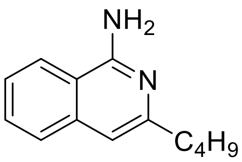

A mixture of 2-bromobenzamidine hydrochloride **79** (0.353 g, 1.498 mmol), BINAP (0.051 g, 0.082 mmol, 0.027 eq) and $PdCl_2(MeCN)_2$ (0.019 g, 0.073 mmol, 0.025 eq) was sealed in a microwave vessel with magnetic bar. It was purged and refilled with nitrogen for 5 min. Then a solution of 1-hexyne (0.492 g, 5.998 mmol, 4 eq), DBU (0.560 ml, 3.740 mmol, 2.5 eq) in dry DMF (6 ml) was added. The reaction was kept stirring under N_2 for a further 5 min. Then the mixture was irradiated by microwave at 120 °C for 1 h. The solution was allowed to cool, AcOEt (25 ml) added, and then it was washed with a saturated solution of $NaHCO_3$ (3x10 ml). The organic layer was dried over $MgSO_4$, filtered and the solvents evaporated. The crude mixture was purified by column chromatography eluting with DCM then AcOEt: PE 1:1. Finally

the product was recrystallised using 1:1 DCM:PE to give isoquinoline **22** (0.1 g, 33 %). **Mp** 90-92 °C. **^{1}H NMR** (500 MHz, Chloroform-*d*) δ 7.84 (d, *J* = 8.5 Hz, 1H), 7.67 – 7.57 (m, 2H), 7.43 (m, 1H), 6.84 (s, 1H), 5.73 (s, 2H-NH_2), 2.72 (t, *J* = 7.8 Hz, 2H), 1.82 – 1.63 (m, 2H), 1.41 (h, *J* = 8.0 Hz, 2H), 0.95 (t, *J* = 8.0 Hz, 3H).

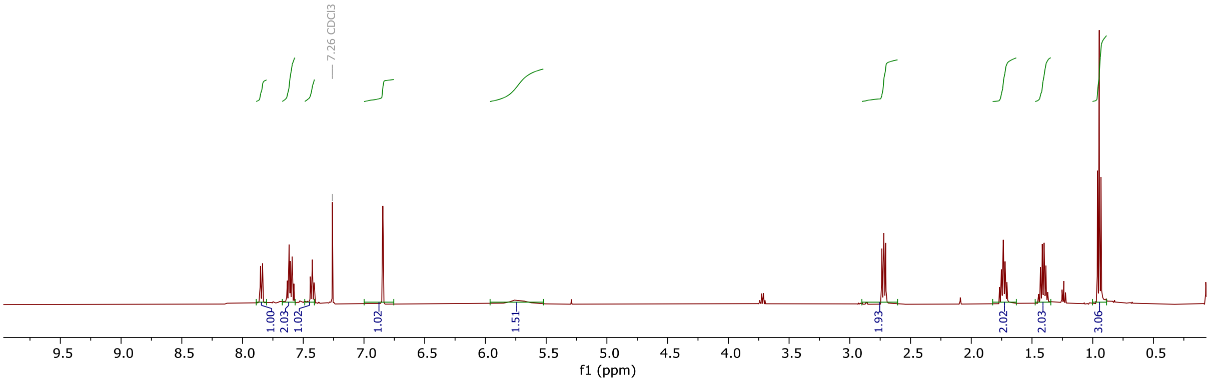

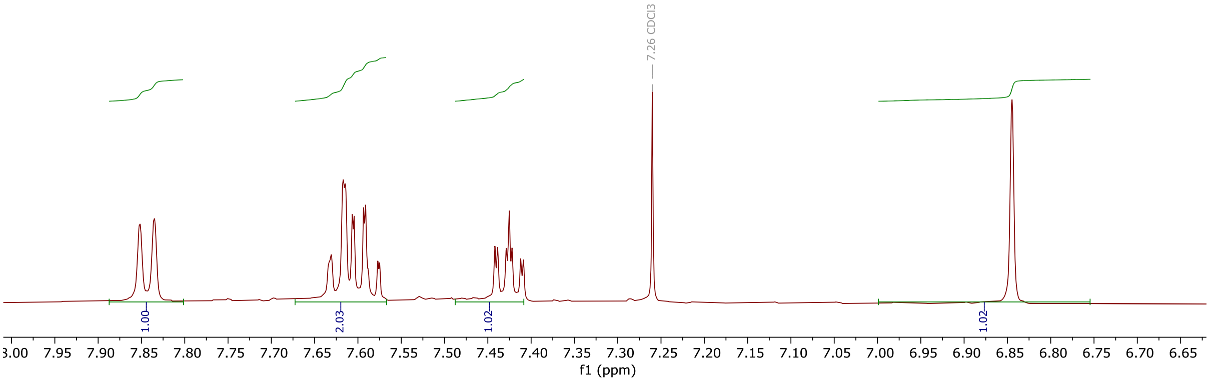

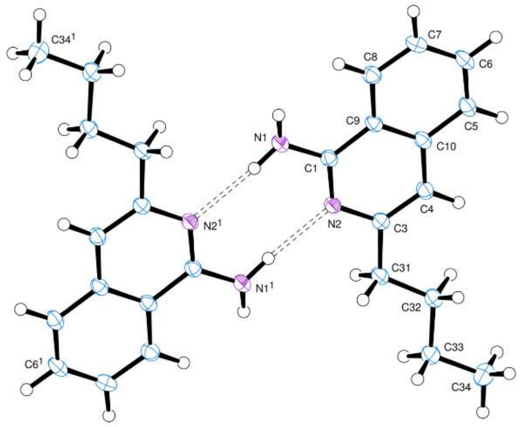

<u>General synthesis of symmetrical aza-dibenzodipyrromethenes</u>

In a typical procedure, aminoisoindoline derivatives (500 mg) were refluxed in toluene (10 ml), diglyme (in the synthesis of compounds **4f-4i**), or p-xylene (in the synthesis of **23** and bis-3-hydroxyphenyl aza-DBDPM **4k** from **1k**), for 24 h. After evaporation of the solvents, the crude mixture was purified by column chromatography eluting with DCM. The resulting red solid was recrystallised from 1:1 DCM and methanol.

Aza-DBDPM 4b

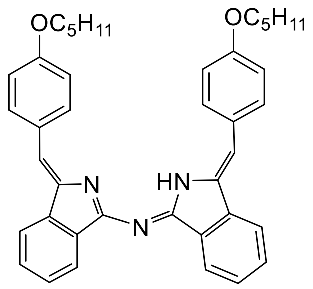

Following the general procedure, aza-DBDPM **4b** was isolated as red crystals (390 mg, 80%). **Mp** 150-152 °C. **^{1}H NMR** (400 MHz, Methylene Chloride-*d*$_2$) δ 8.06 (br-s, 1H), 7.87 – 7.80 (m, 3H), 7.57 (td, *J* = 7.5, 1.1 Hz, 1H), 7.51 (td, *J* = 7.5, 1.0 Hz, 1H), 6.84 (s, 1H), 6.62 (d, *J* = 8.6 Hz, 2H), 3.79 (t, *J* = 6.7 Hz, 2H), 1.81 – 1.69 (m, 2H), 1.48 – 1.37 (m, 4H), 0.95 (t, *J* = 6.9 Hz, 3H). **^{13}C NMR** (126 MHz, Chloroform-*d*) δ 164.37, 158.73, 145.49, 143.18, 131.98, 130.62, 129.31, 128.90, 126.72, 119.56, 118.75, 115.79, 114.60, 68.02, 28.98, 28.22, 22.50, 14.04.

MS (MALDI-TOF): ***m/z*** **=** 596 [M+H]. **UV−Vis** (CH_2Cl_2) λ_{max}/nm (ε/dm^3.mol^{-1}.cm^{-1}): 363 (8.9x10^4), 416 (2.2x10^4), 505 (1.6x10^4).

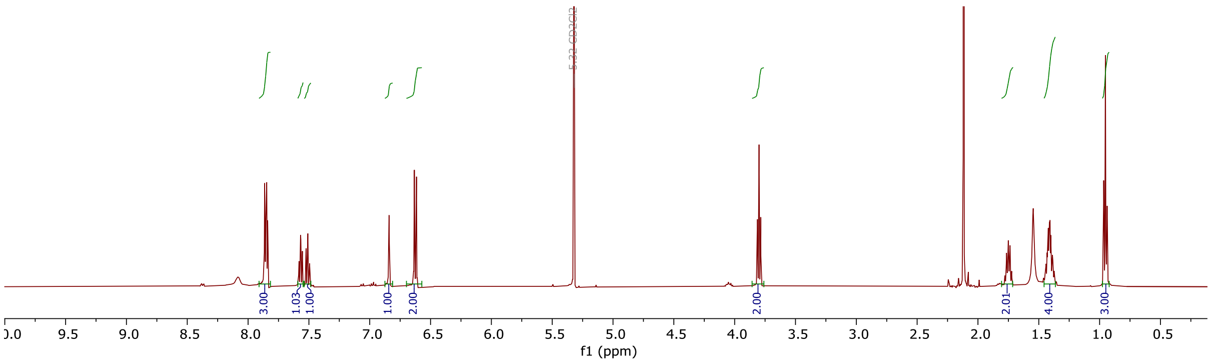

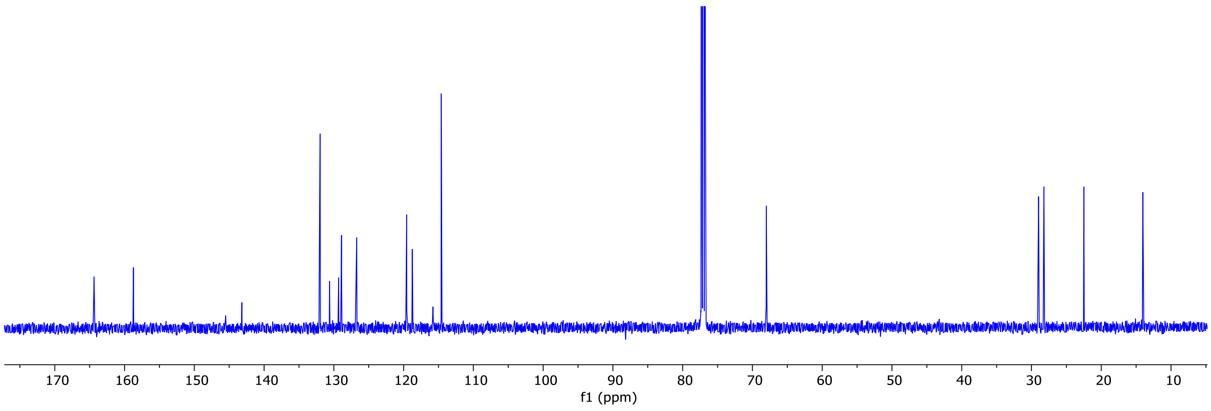

Aza-DBDPM 4c

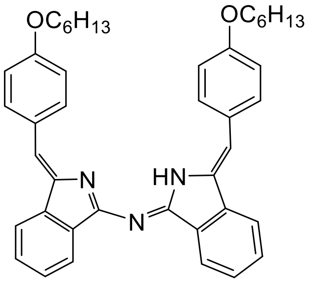

Following the general procedure, aza-DBDPM **4c** was isolated as red crystals (80 mg, 14 %). **Mp** 155-157 °C. **^{1}H NMR** (500 MHz, Acetone-d_6) δ 8.04 (d, *J* = 7.7 Hz, 2H), 8.02 (d, *J* = 7.7 Hz, 2H), (m, 4H), 7.94 (d, *J* = 8.7 Hz, 4H), 7.62 (td, *J* = 7.5, 1.2 Hz, 2H), 7.55 (td, *J* = 7.4, 1.0 Hz, 2H), 7.07 (s, 2H), 6.71 (d, *J* = 8.7 Hz, 4H), 3.89 (t, *J* = 6.6 Hz, 4H), 1.77 (m, 4H), 1.49 (m, 4H), 1.41 – 1.34 (m, 8H), 0.92 (t, *J* = 6.7 Hz, 6H) ppm. ^{13}C NMR (126 MHz, Chloroform-d + THF) δ 164.77, 158.19, 139.31, 139.19, 133.91, 130.42, 129.11, 127.60, 126.98, 121.43, 118.48, 114.33, 113.37, 67.32, 31.03, 28.65, 25.20, 21.98, 13.19. **MS (MALDI-TOF):** ***m/z*** **=** 624 [M+H]. **UV−Vis** (CH_2Cl_2) λ_{max}/nm (ε/dm^3.mol^{-1}.cm^{-1}): 362 (6.8×10^4), 403(shoulder) (1.5×10^4), 498 (1.2×10^4).

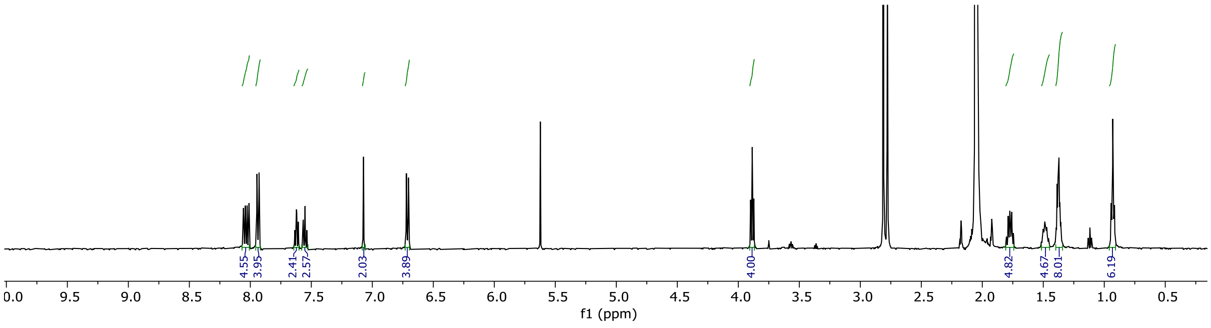

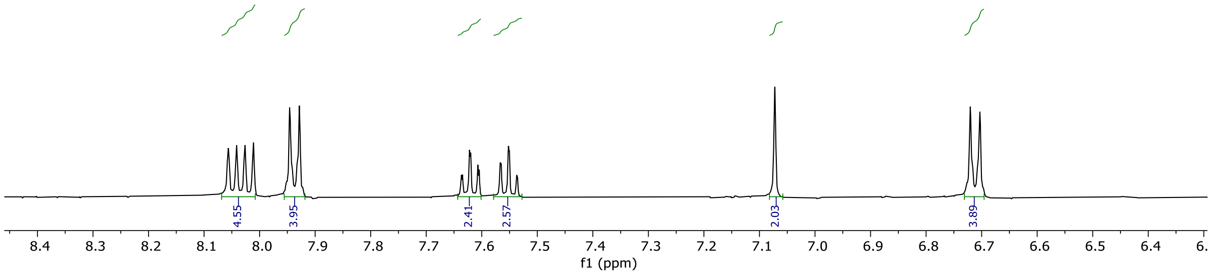

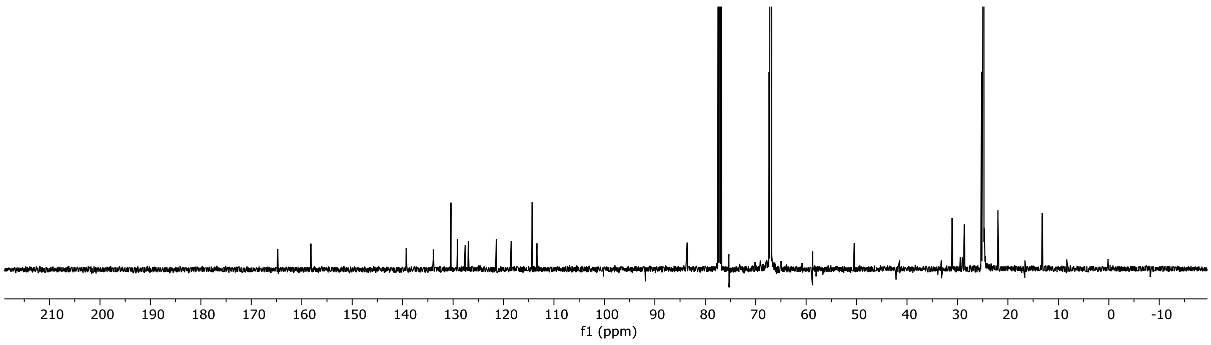

Aza-DBDPM 4d

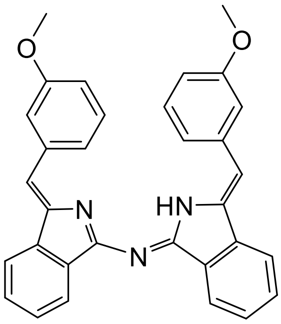

Following the general procedure, aza-DBDPM **4d** was isolated as red crystals (410 mg, 84 %). **Mp** 140-142 °C. **^{1}H NMR** (500 MHz, Chloroform-*d*) δ 13.37 (br-s, 1H), 8.07 (d, *J* = 7.1 Hz, 1H), 7.80 (d, *J* = 7.4 Hz, 1H), 7.54 (td, *J* = 7.4, 1.4 Hz 1H), 7.51-7.48 (m, 2H), 7.44 (d, *J* = 7.4 Hz, 1H), 6.98 (t, *J* = 7.7 Hz, 1H), 6.75 (s, 1H), 6.58 (dd, *J* = 7.9, 2.6 Hz, 1H), 3.65 (s, 3H). **^{13}C NMR** (126 MHz, Chloroform-*d*) δ 164.49, 159.10, 145.81, 143.20, 132.00, 130.71, 129.60, 128.90, 126.76, 119.57, 118.74, 115.63 (br, multiple C), 114.00, 55.31. **MS (MALDI-TOF): *m/z* =** 483 [M, 100 %]. **UV−Vis** (CH_2Cl_2) λ_{max}/nm (ε/dm^3.mol^{-1}.cm^{-1}): 341 (9.5x10^4), 421 (2.0x10^4), 477 (2.3x10^4).

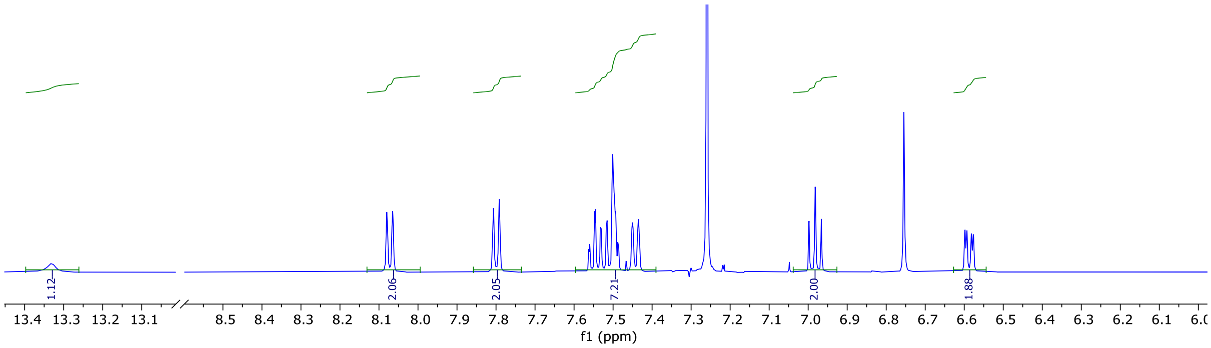

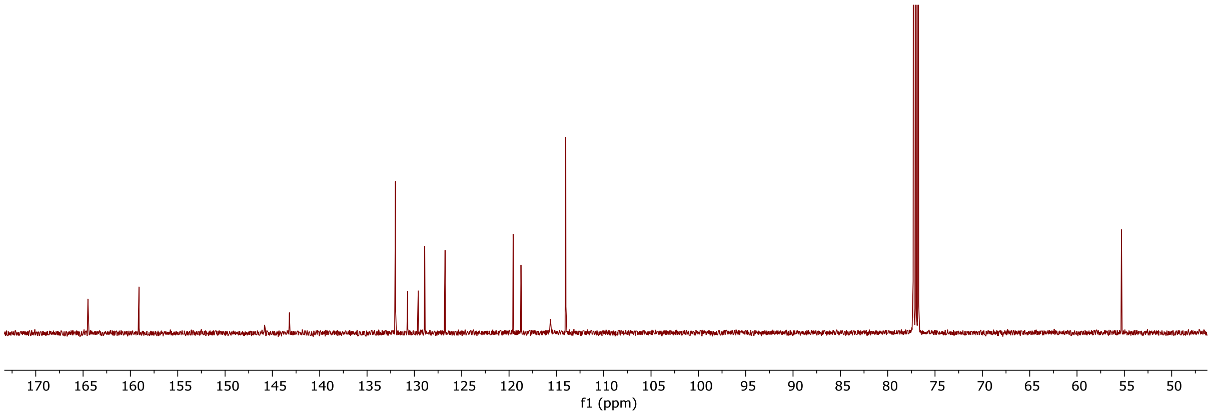

Aza-DBDPM 4f

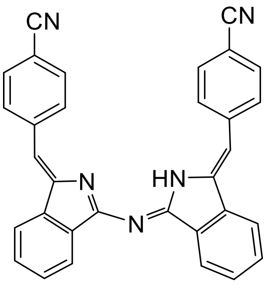

Following the general procedure, aza-DBDPM **4f** was isolated as red crystals (290 mg, 60 %). **Mp** 290-292 °C. **^{1}H NMR** (500 MHz, Acetone-d_6) δ 13.56 (brs, 1H), 8.13 (d, *J*=8.3 Hz, 2H), 8.07 (td, *J* = 7.6, 1.0 Hz, 1H), 8.04 (td, *J* = 7.4, 1.0 Hz, 1H), 7.69 (td, *J* = 7.4, 1.1 Hz, 1H), 7.63 (td, *J* = 7.4, 1.0 Hz, 1H), 7.44 (d, *J*=8.3 Hz, 2H), 7.14 (s, 1H). **^{13}C NMR** (126 MHz, Chloroform-*d*) δ 141.20, 133.29, 131.95, 131.06, 130.20, 130.05, 123.10 121.43, 113.58, 111.76. **MS (MALDI-TOF): *m/z* =** 473 [M, 100 %]. **UV−Vis** (CH_2Cl_2) λ_{max}/nm (ε/dm^3.mol^{-1}.cm^{-1}): 358 (9.3x10^4), 492 (1.2x10^4).

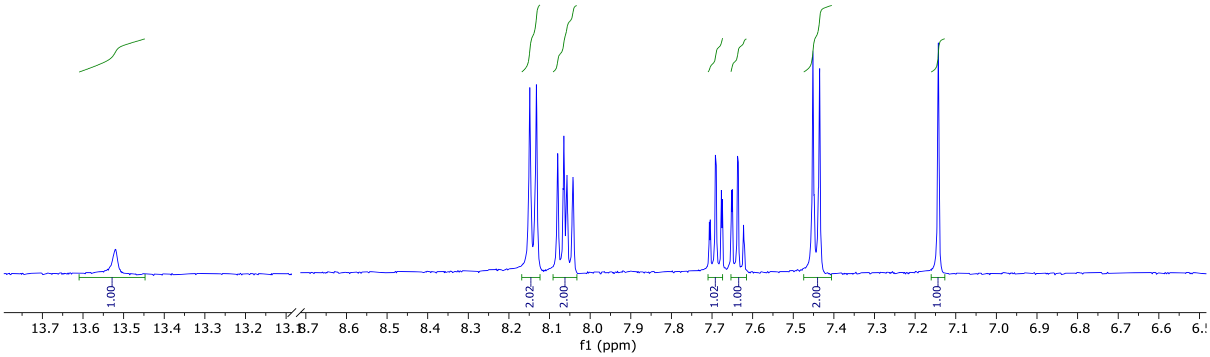

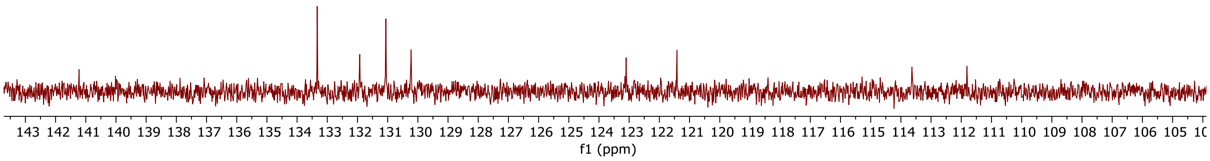

Aza-DBDPM 4k

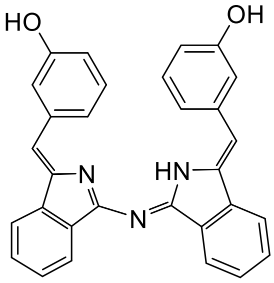

Following the general procedure, aza-DBDPM **4g** was isolated as a red-brown powder (250 mg, 51 %). **Mp** 168-170 °C. **^{1}H NMR** 500 MHz, Acetone-d_6) δ 12.60 (br-s, 1H), 8.33 (br-s, 2H), 8.06-8.02 (m, 4H), 7.63 (td, *J* = 7.2, 1.0 Hz 2H), 7.59 – 7.54 (m, 4H), 7.41 (t, *J* = 2.1 Hz, 2H), 7.03 (s, 2H), 6.90 (t, *J* = 7.9 Hz, 2H), 6.67 (ddd, *J* = 8.1, 2.5, 0.9 Hz, 2H). Solubility too low to achieve useful ^{13}C NMR spectra. **MS (MALDI-TOF): *m/z* =** 455 [M, 100 %]. **UV−Vis** (CH_2Cl_2) λ_{max}/nm (ε/dm^3.mol^{-1}.cm^{-1}): 349 (8.73x10^4), 469 (2.3x10^4).

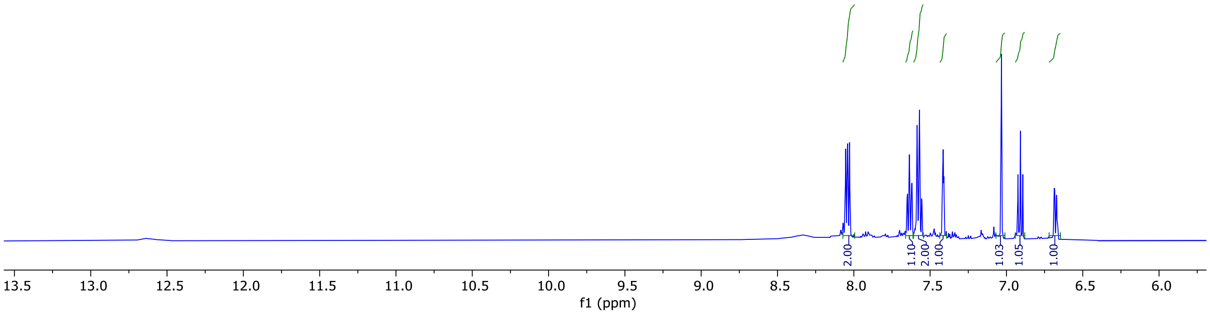

Aza-DBDPM ditriflate 29

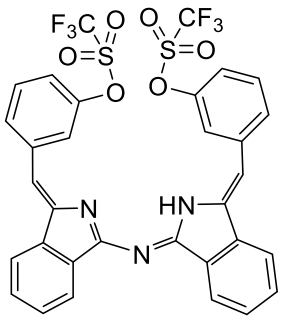

To a stirred solution of aza DBDPM **4g** (0.250 g, 0.549 mmol), and pyridine (0.1 ml, 0.823 mmol, 1.5 eq) in dry DCM (30 ml), trifluoromethanesulfonic acid anhydride (0.24 ml, 0.823 mmol, 1.5 eq) was added dropwise at -20 °C over 30 min under nitrogen. The resulting mixture was left overnight at room temperature. After dilution with H_2O (60 ml), the mixture was extracted with DCM (3x15 ml). The solvent was evaporated in vacuo and the obtained product was purified by column chromatography (eluting with 1:3 DCM:PE) and recrystallized from DCM and MeOH to give ditriflate **29** as a poorly soluble amorphous solid (98 mg, 24 %). **^{1}H NMR** (500 MHz, Acetone-d_6) δ 13.37 (br-s, 1H), 8.20 (d, J = 7.5 Hz, 2H), 8.05-8.01 (m, 4H), 7.95 (s, 2H), 7.67 (t, J = 7.6 Hz 2H), 7.62 (t, J = 7.6 Hz 2H), 7.20 (td, J = 7.6, 1.0 Hz, 2H), 7.13 – 7.10 (m, 4H). **MS (MALDI-TOF): *m/z* =** 719 [M,100 %].

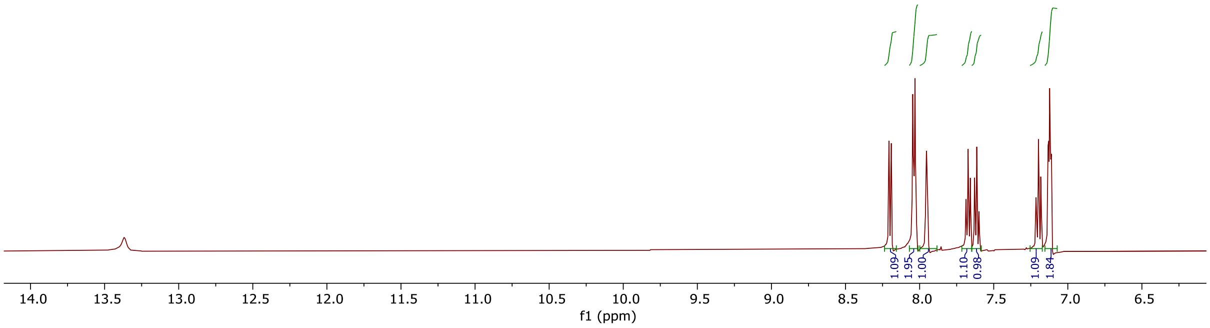

Aza-DBDPM 25

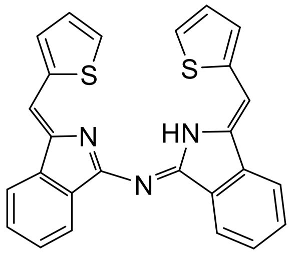

Following the general procedure, aza-DBDPM **25** was isolated as red crystals (370 mg, 77 %). **Mp** 192-195 °C. **^{1}H NMR** (500 MHz, Acetone-d_6) δ 9.15 (s, 1H), 8.06 (dt, J = 7.5, 1.0 Hz, 1H), 8.03 (dt, J = 7.7, 0.9 Hz, 1H), 7.64 (td, J = 7.4, 1.2 Hz, 1H), 7.62 – 7.56 (m, 2H), 7.41 (dd, J = 5.1, 1.1 Hz, 1H), 7.32 (s, 1H), 7.05 (dd, J = 5.1, 3.6 Hz, 1H). **^{13}C NMR** (126 MHz, Acetone-d_6) δ 139.07, 138.71, 136.87, 135.01, 130.42, 129.41, 129.05, 128.30, 127.99, 123.67, 122.12, 119.77, 108.17. **MS (MALDI-TOF): *m/z* =** 435 [M, 100 %]. **UV−Vis** (CH_2Cl_2) λ_{max}/nm (ε/dm^3.mol^{-1}.cm^{-1}): 315 (5.1x10^4), 360 (8.6x10^4), 505 (1.9x10^4).

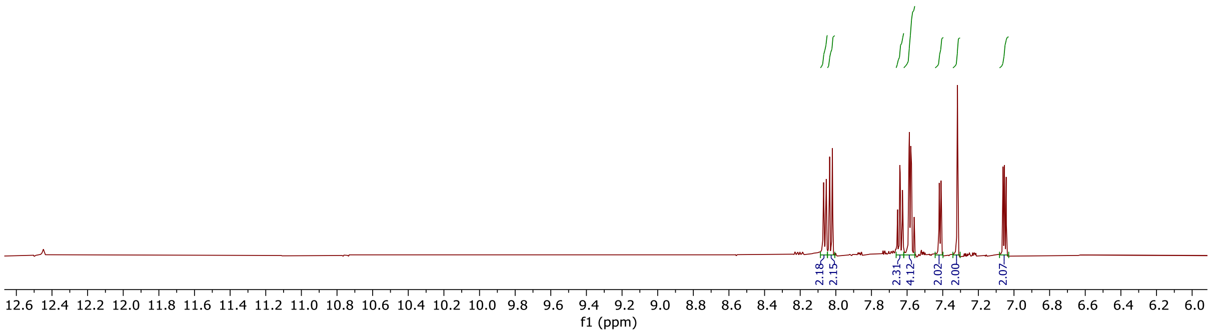

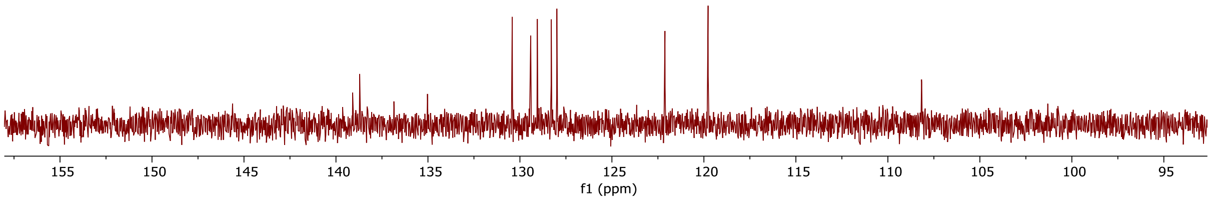

Aza-DBDPM 4g

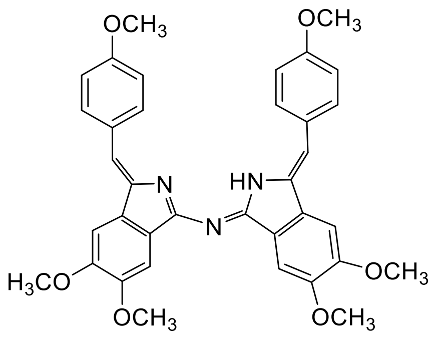

Following the general procedure, aza-DBDPM **4g** was isolated as red crystals (38 mg, 30 %). **Mp** 280-284 °C. **^{1}H-NMR** (500 MHz, methylene chloride-d_2): δ 12.80 ((s, 1H, N-H), 7.83 (d, J = 8.2 Hz, 4H), 7.50 (s, 2H), 7.25 (s, 2H), 6.66 (s, 2H), 6.62 (d, J = 8.2 Hz, 4H), 3.99 (s,12H), 3.69 (s, 6H). **^{13}C-NMR** (126 MHz, CD_2Cl_2) δ 166.14, 159.53, 152.59, 150.93, 140.76, 134.05, 131.49, 129.19, 127.93, 115.14, 113.49, 104.10, 102.15, 56.74, 56.66, 55.51. **MS (MALDI-TOF): *m/z*** 605.89 [M]$^+$ (100%). **UV−Vis** (CH_2Cl_2) λ_{max}/nm (ε/dm^3.mol^{-1}.cm^{-1}): 484 (1.63 x 10^4)

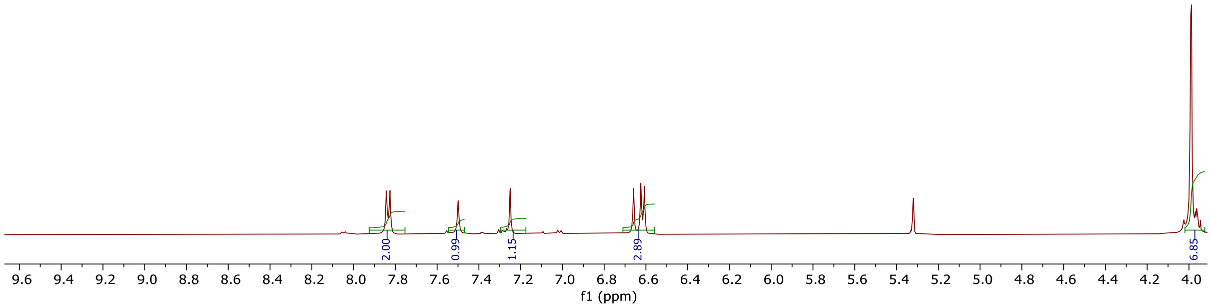

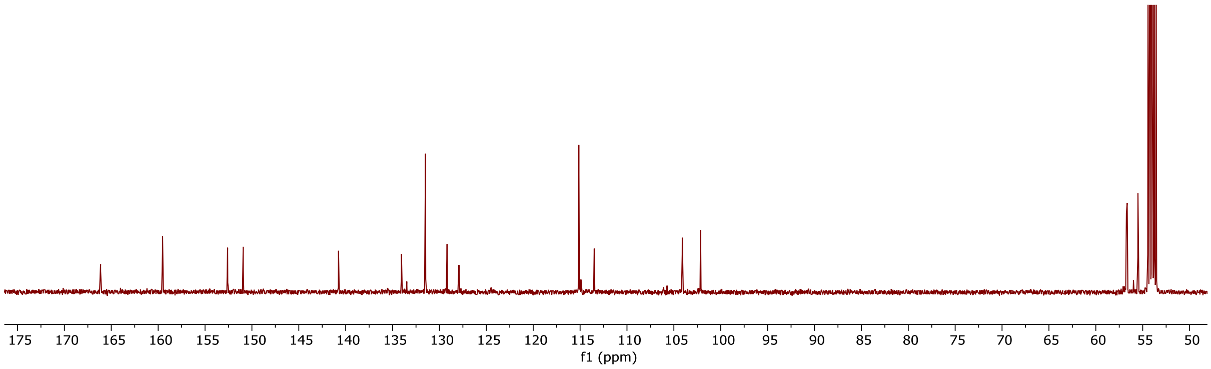

Aza-DBDPM 4h

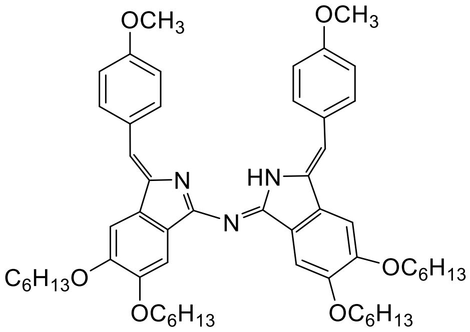

Following the general procedure, aza-DBDPM **4h** was isolated as red crystals (90 mg, 49 %). **Mp** 112-114 °C. **^{1}H-NMR** (500 MHz, Methylene Chloride-d_2) δ 12.78 (s, 1H, N-H), 7.83 (d, J = 8.8 Hz, 4H), 7.50 (s, 2H), 7.27 (s, 2H), 6.65 (s, 2H), 6.61 (d, J = 8.8 Hz, 4H), 4.16-4.12 (m, 8H), 3.69 (s, 6H, OCH_3), 1.91- 1.85 (m, 8H), 1.56- 1.51 (m, 8H), 1.42- 1.36 (m, 16H), 0.95- 0.92 (m, 12H). **^{13}C-NMR** (126 MHz, CD_2Cl_2) δ 166.30, 159.48, 152.49, 150.87, 140.92, 134.00, 131.43, 129.28, 128.05, 115.12, 113.13, 105.73, 103.90, 69.97, 55.52, 32.18, 29.86, 29.81, 26.31, 23.21, 14.39. **MS (MALDI-TOF): *m/z*** 884.39 $[M]^+$ (100%). **UV−Vis** (CH_2Cl_2) λ_{max}/nm (ε/dm^3.mol^{-1}.cm^{-1}): 476 (1.51 x 10^4)

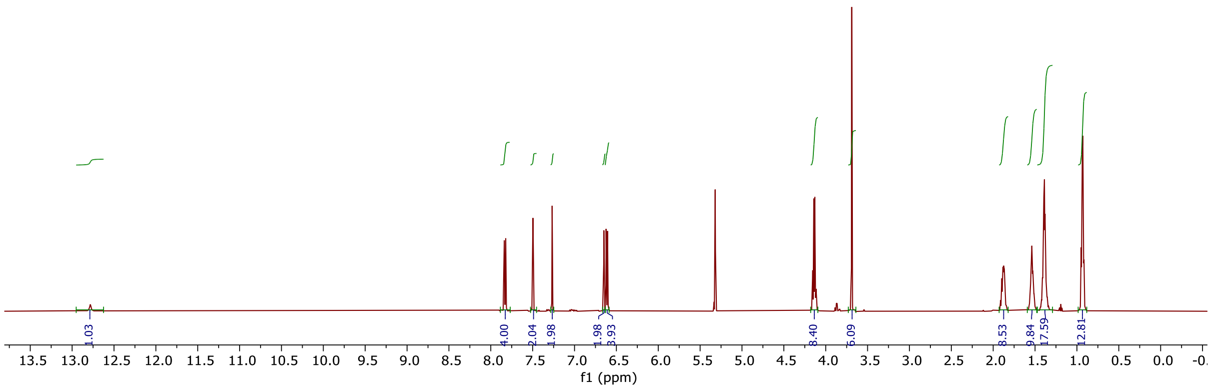

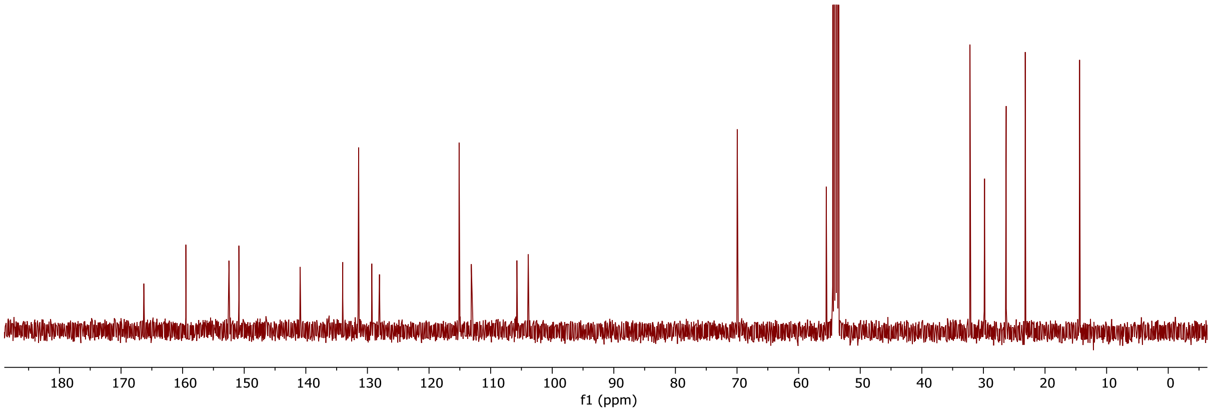

Aza-DBDPM 4i

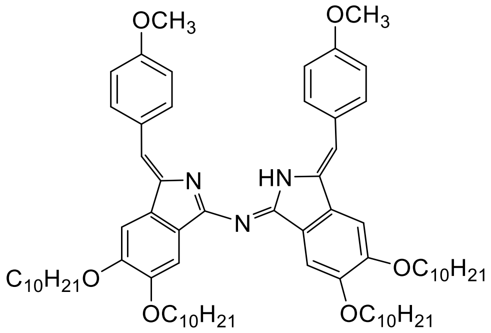

Following the general procedure, aza-DBDPM **4i** was isolated as red crystals (98 mg, 43 %). **Mp** 90-92 °C. **^{1}H-NMR** (500 MHz, Methylene Chloride-*d*$_2$) δ 12.78 (s, 1H, N-H), 7.83 (d, *J* = 8.8 Hz, 4H), 7.52 (s, 2H), 7.26 (s, 2H), 6.64 (s, 2H), 6.61 (d, *J* = 8.8 Hz, 4H), 4.13- 4.10 (m, 8H), 3.69 (s, 6H, OCH_3), 1.91- 1.83 (m, 8H), 1.57- 1.48 (m, 8H), 1.42- 1.29 (m, 48H), 0.90- 0.88 (m, 12H). **^{13}C-NMR** (126 MHz, CD_2Cl_2) δ 166.32, 159.46, 152.47, 150.88, 140.93, 133.99, 131.44, 129.31, 128.07, 115.12, 113.12, 105.77, 103.92, 69.98, 69.94, 55.51, 32.52, 30.25, 30.21, 30.19, 30.04, 30.03, 29.96, 29.93, 29.87, 26.67, 26.66, 23.29, 14.47. **MS (MALDI-TOF):** ***m/z*** 1106.60 $[M]^+$ (100%). **UV−Vis** (CH_2Cl_2) λ_{max}/nm (ε/$dm^3.mol^{-1}.cm^{-1}$): 446 (1.51 x 10^4)

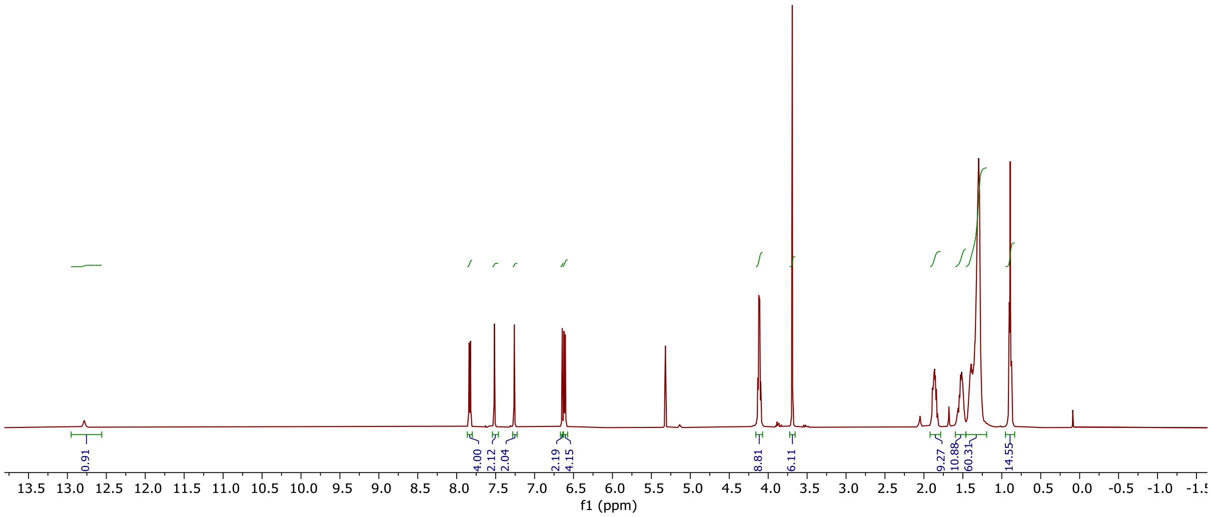

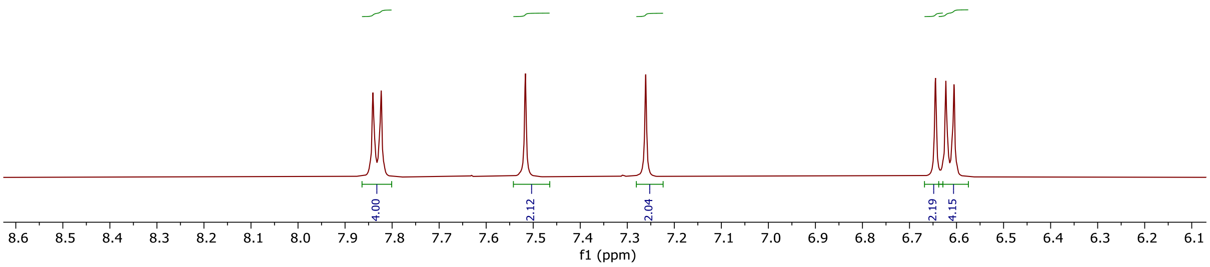

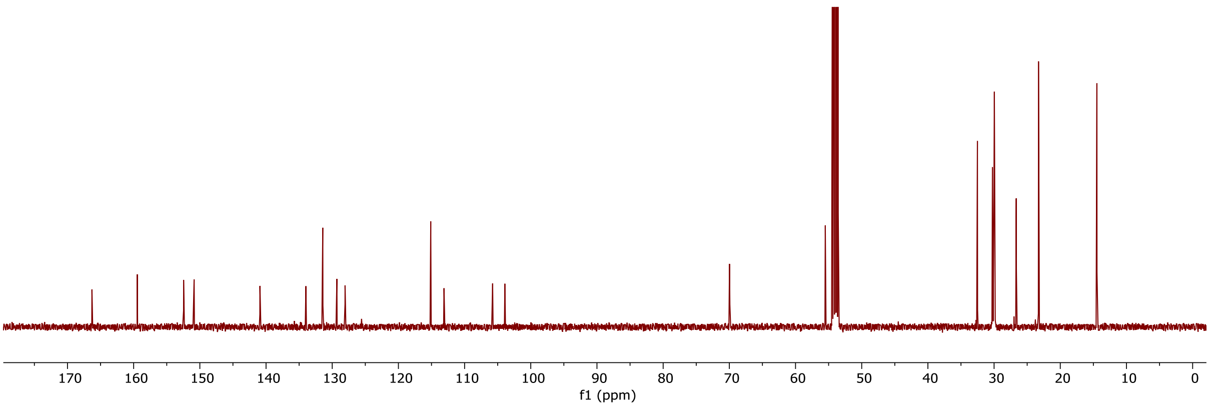

Aza-DBDPM 4j

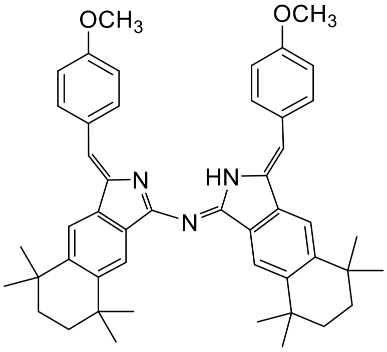

Following the general procedure, aza-DBDPM **4j** was isolated as red crystals (89 mg, 61 %). **Mp** 82-84 °C. **^{1}H-NMR** (500 MHz, Methylene Chloride-d_2) δ 13.02 (s, 1H, N-H), 8.04 (s, 2H), 7.84 (d, J = 8.7 Hz, 4H), 7.79 (s, 2H), 6.77 (s, 2H), 6.61 (d, J = 8.7 Hz, 4H), 3.69 (s, 6H, OCH_3), 1.79 (s, 8H), 1.45 (s, 12H), 1.42 (s, 12H). **^{13}C-NMR** (126 MHz, CD_2Cl_2) δ 166.43, 159.46, 148.57, 146.58, 140.74, 137.71, 132.85, 131.42, 129.24, 120.54, 117.88, 115.10, 113.01, 55.52, 35.67, 35.64, 35.57, 35.55, 32.54, 32.50. **MS (MALDI-TOF): *m/z*** 704.41 $[M]^+$ (100%). **UV−Vis** (CH_2Cl_2) λ_{max}/nm (ε/dm^3.mol^{-1}.cm^{-1}): 476 (1.41 x 10^4).

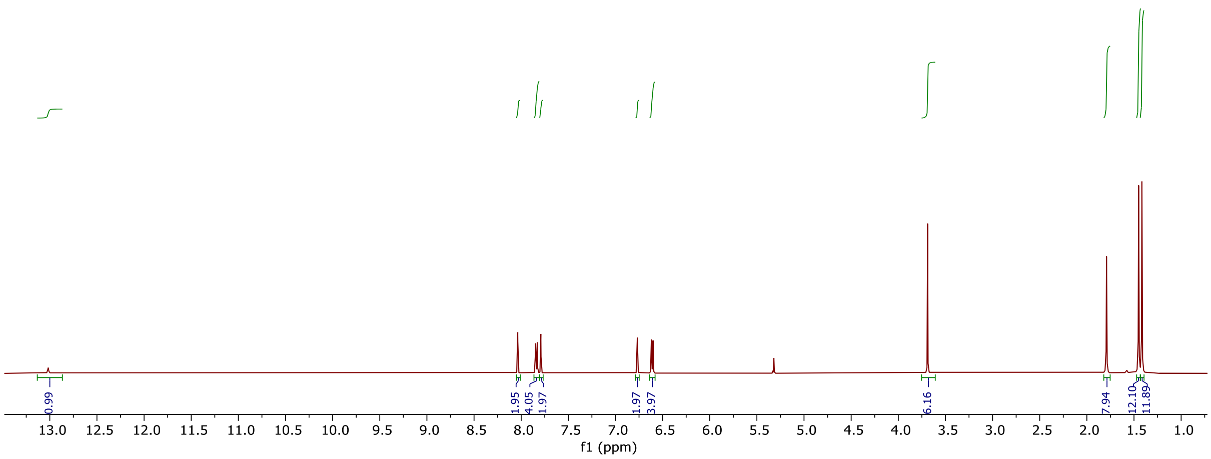
0.99
1.95
4.05
1.97
1.97
3.97
6.16
7.94
12.10
11.89
13.0 12.5 12.0 11.5 11.0 10.5 10.0 9.5 9.0 8.5 8.0 7.5 7.0 6.5 6.0 5.5 5.0 4.5 4.0 3.5 3.0 2.5 2.0 1.5 1.0
f1 (ppm)

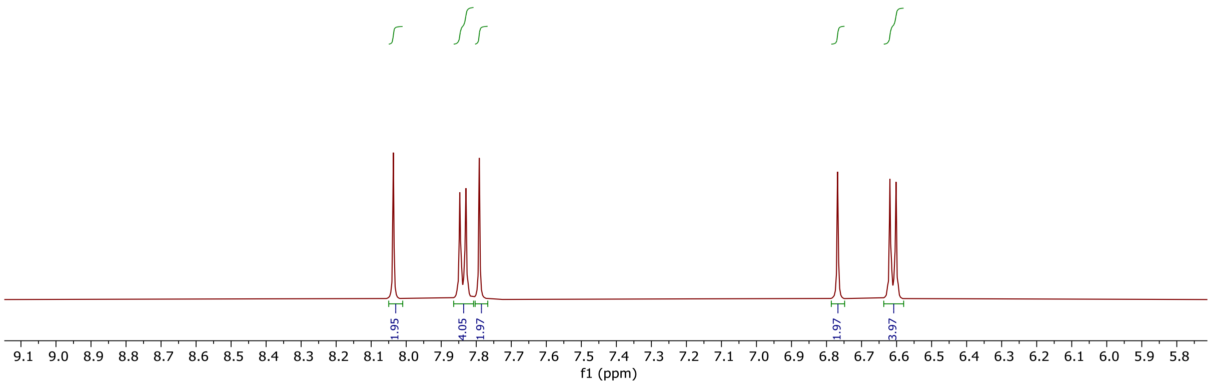
1.95
4.05
1.97
1.97
3.97
9.1 9.0 8.9 8.8 8.7 8.6 8.5 8.4 8.3 8.2 8.1 8.0 7.9 7.8 7.7 7.6 7.5 7.4 7.3 7.2 7.1 7.0 6.9 6.8 6.7 6.6 6.5 6.4 6.3 6.2 6.1 6.0 5.9 5.8
f1 (ppm)

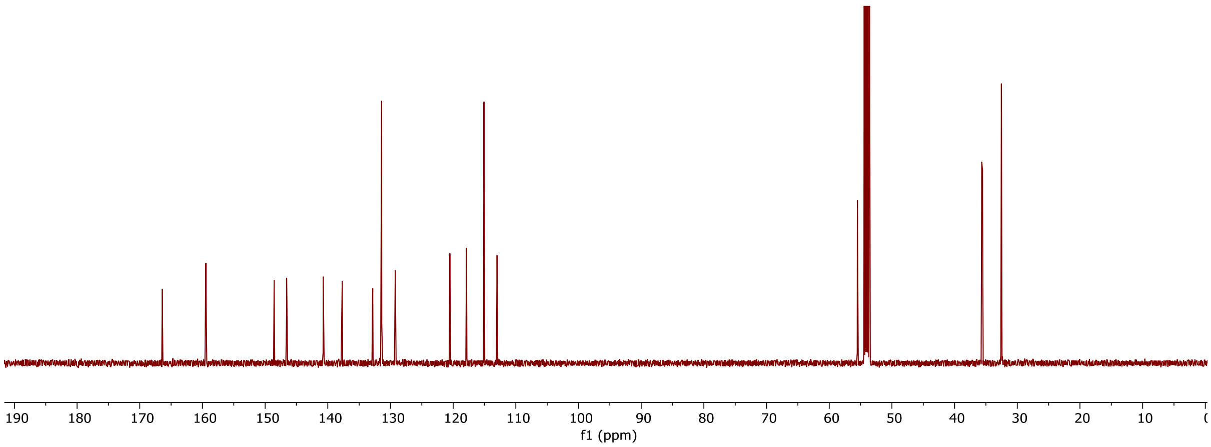
190 180 170 160 150 140 130 120 110 100 90 80 70 60 50 40 30 20 10
f1 (ppm)

Aza-DBDPM 23

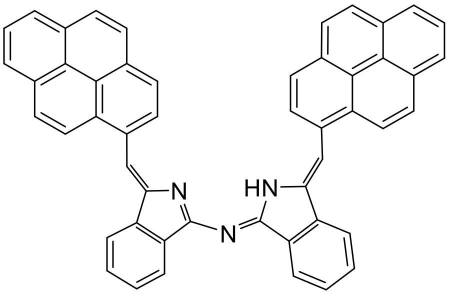

Following the general procedure, aza-DBDPM **23** was isolated as red crystals from THF/Hexane (58 mg, 12 %). **Mp** >300 °C. **^{1}H-NMR** (500 MHz, THF-d_8) δ 13.68 (s, 1H, N-H), 8.78 (d, J = 8.4 Hz, 2H), 8.65 (d, J = 8.9 Hz, 2H), 8.27-8.21 (m, 6H), 8.14-8.09, (m, 4H), 8.03 (t, J = 7.6 Hz, 2H), 7.93 (s, 2H), 7.65 (dt, J = 7.6, 1.0 Hz, 2H), 7.57 (dt, J = 7.3, 1.0 Hz, 2H), 7.48 (d, J = 8.4 Hz, 2H), 6.83 (d, J = 8.0 Hz, 2H), 6.65 (d, J = 8.6 Hz, 2H). **MS (MALDI-TOF):** ***m/z*** 671.62 $[M]^+$ (100%). **UV−Vis** (THF) λ_{max}/nm (ε/dm^3.mol^{-1}.cm^{-1}): 412 (4.5x10^4), 398 (4.4x10^4).

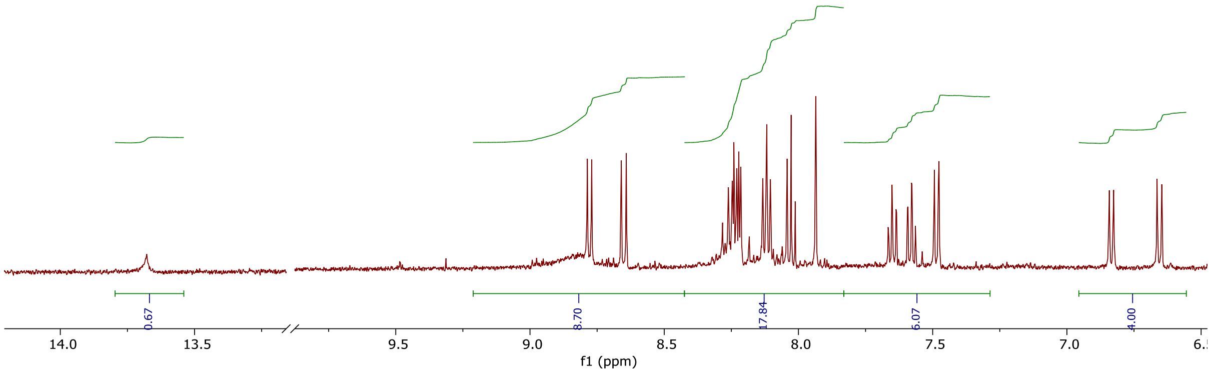

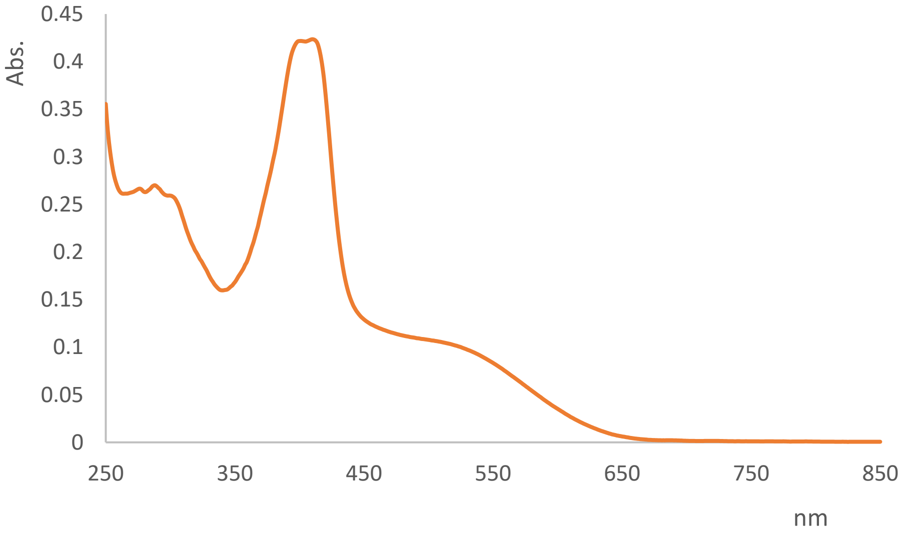

Absorption Spectrum of **23** (in THF).

Aza-DBDPM 31

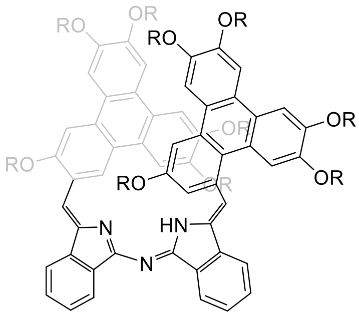

31

Following the general procedure, aza-DBDPM **31** was isolated as a red solid after reprecipitation from DCM/EtOH (70 mg, 13 %). **Mp** 263-265 °C. **MS (MALDI-TOF):** ***m/z*** 1724 [M$^+$]. **UV−Vis** (THF) λ_{max}/nm (ε/dm^3.mol^{-1}.cm^{-1}): 400 (3.7x10^4), 455 (shoulder), 550 (0.3x10^4).

<u>General difluoroborylation of aza-dibenzodipyrromethenes (synthesis of aza-dibenzoBODIPYs)</u>

In a typical procedure, azaDPM derivatives (100 mg, 1eq) were stirrred in dry dichloromethane (DCM), then DBU (10 eq) and BF_3.OEt_2 (50eq) were added and the mixture was left stirring for 24 h. Trimethylsilyl chloride (1.5 mL) was added and the mixture was refluxed for 3 h. After cooling, the mixture was diluted with DCM (50 mL) and washed with 1M HCl (50 mL). Evaporation of the solvents and recrystallisation from DCM/Petroleum ether gave the pure product.

Aza-DBBODIPY 5d

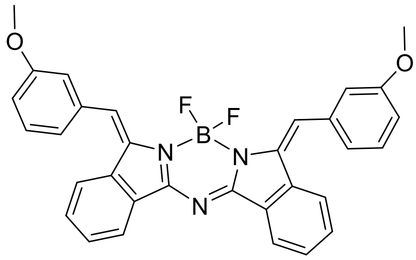

Following the general procedure, aza-DBBODIPY **5d** was isolated as orange crystals (80 mg, 73 %). **Mp** 190-192 °C. **^{1}H NMR** (500 MHz, Chloroform-*d*) δ 8.18 (d, *J* = 7.7 Hz, 2H), 7.84 (s, 2H), 7.70 (d, *J* = 7.9 Hz, 2H), 7.54 (td, *J* = 7.5, 1.0 Hz, 2H), 7.46 (ddd, *J* = 8.3, 7.4, 1.2 Hz, 2H), 7.40 (t, *J* = 7.7 Hz, 2H), 7.22 (d, *J* = 7.4 Hz, 2H), 7.15 (t, *J* = 1.6 Hz, 2H), 6.98 (dd, *J* = 8.3, 1.6 Hz, 2H), 3.85 (s, 6H). **^{13}C NMR** (126 MHz, Chloroform-*d*) δ 163.79, 159.75, 138.71, 136.26, 133.20, 132.50, 129.72, 129.31, 125.39, 123.72, 123.66, 122.06, 119.71, 114.91, 114.44, 55.40. **^{19}F NMR** (376 MHz, Chloroform-*d*) δ -139.12 (q, *J* = 30.5 Hz). **MS (MALDI-TOF):** ***m/z*** = 531 [M$^+$]. **UV−Vis** (CH_2Cl_2) λ_{max}/nm (ε/dm^3.mol^{-1}.cm^{-1}): 321 (8.2x10^4), 449 (9.0x10^4). **Fluorescence** (CH_2Cl_2, Excitation at 445 nm): 523 nm.

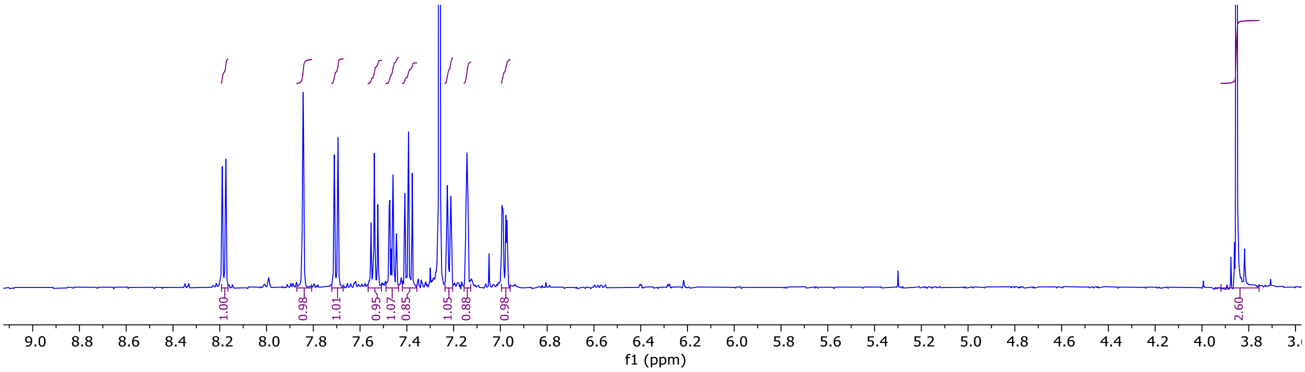

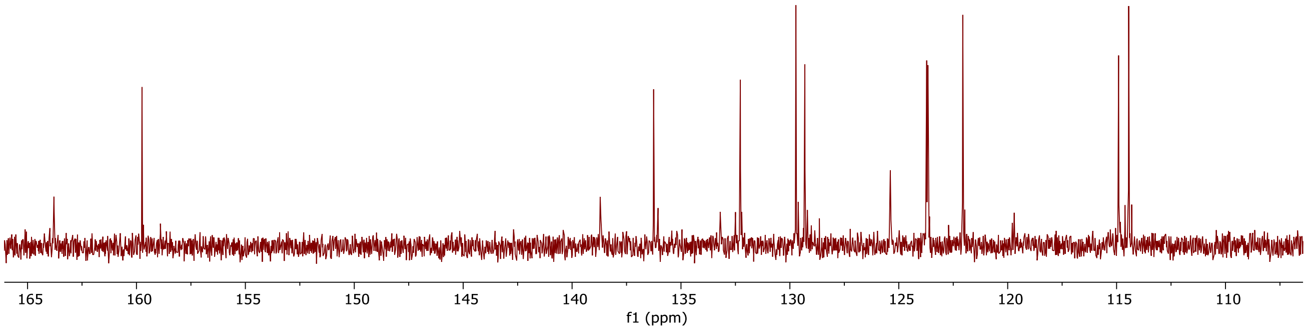
165
160
155
150
145
140
135
130
125
120
115
110
f1 (ppm)

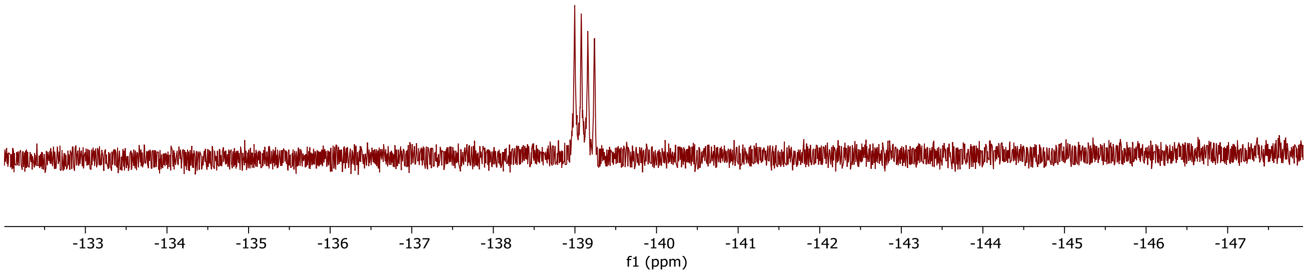
-133
-134
-135
-136
-137
-138
-139
-140
-141
-142
-143
-144
-145
-146
-147
f1 (ppm)

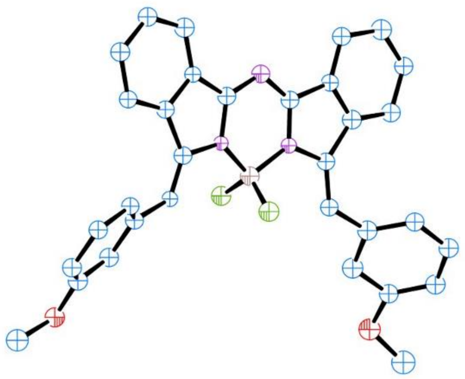

Aza-DBBODIPY 5b

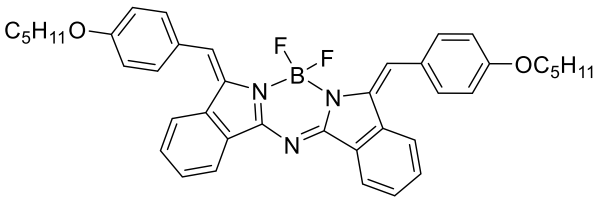

Following the general procedure, aza-DBBODIPY **5b** was isolated as poorly soluble orange crystals (80 mg, 74 %). **Mp** 170-171 °C. **^{1}H NMR** (500 MHz, Methylene Chloride-d_2) δ 8.22 (d, *J* = 7.5 Hz, 2H), 7.85 (d, *J* = 7.9 Hz, 2H), 7.77 (s, 2H), 7.60 – 7.52 (m, 6H), 7.50 (dt, *J* = 7.5, 1.1 Hz, 2H), 7.01 (d, *J* = 8.0 Hz, 4H), 4.05 (t, *J* = 6.6 Hz, 4H), 1.91 – 1.85 (m, 4H), 1.54 – 1.45 (m, 8H), 0.96 (t, *J* = 7.2 Hz, 6H). **^{13}C NMR** (126 MHz, Methylene Chloride-d_2) 159.97, 132.17, 129.15, 126.86, 123.48, 123.24, 114.60, 113.57, 68.22, 28.94, 28.19, 22.48, 13.80. **MS (MALDI-TOF):** ***m/z*** **=** 643 [M$^+$]. **UV−Vis** (CH_2Cl_2) λ_{max}/nm (ε/dm^3.mol^{-1}.cm^{-1}): 324 (6.5x10^4), 475 (7.5x10^4). **Fluorescence** (DCM, Excitation at 475 nm): 561 nm.

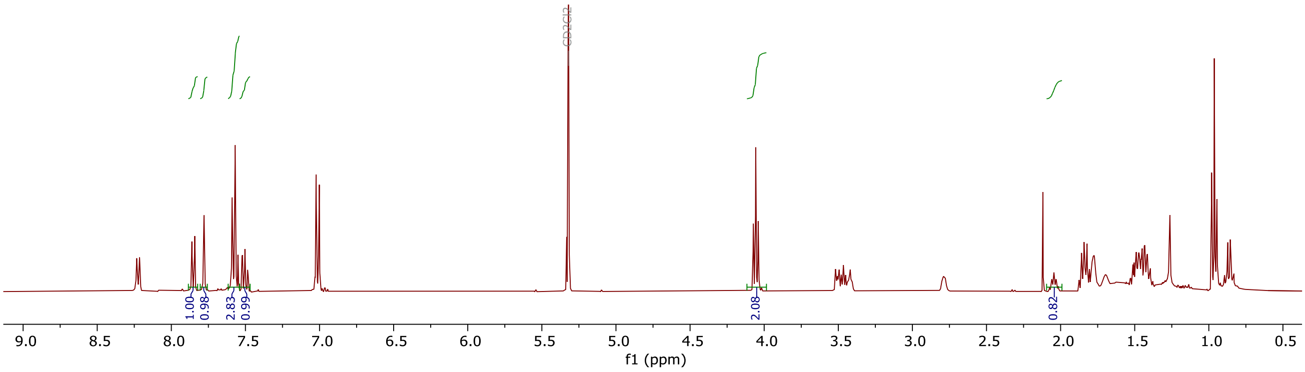

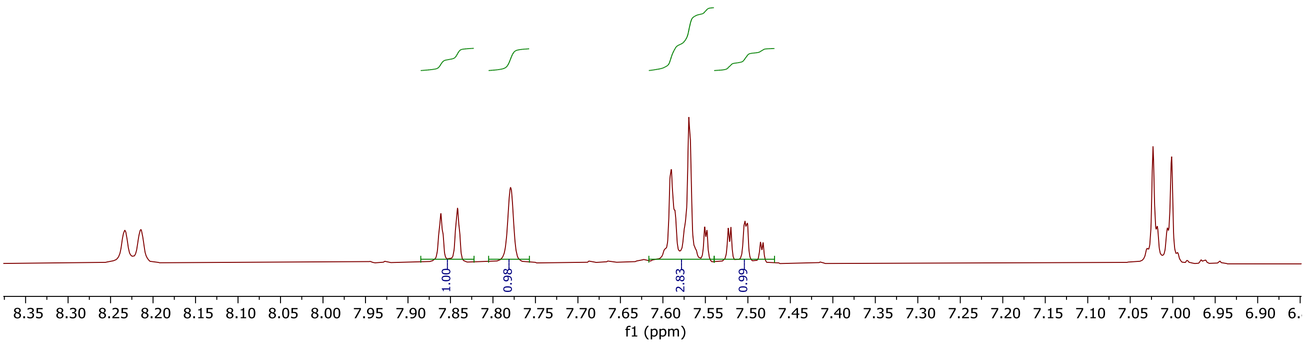

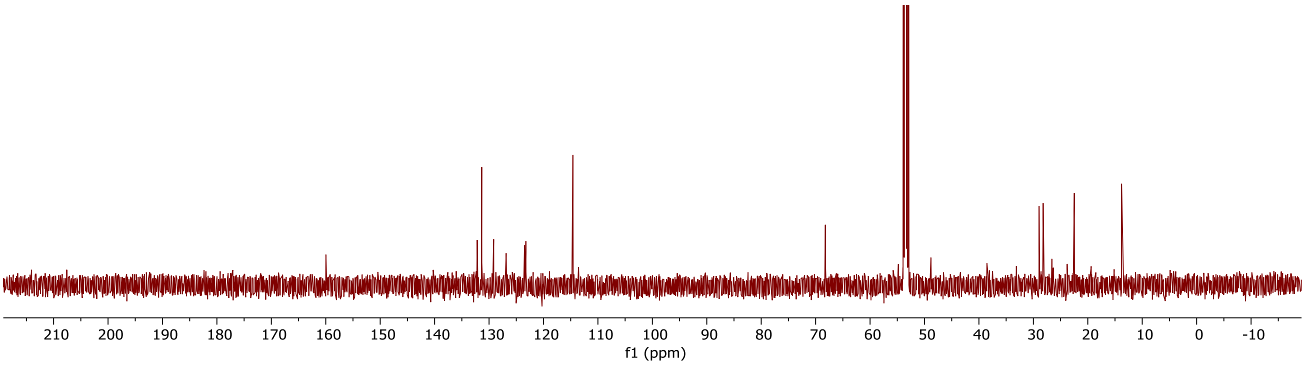

Aza-DBBODIPY 5f

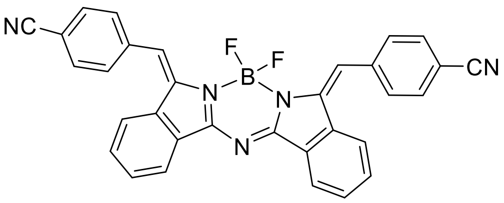

Following the general procedure, aza-DBBODIPY **5f** was isolated as poorly soluble orange crystals (80 mg, 73 %). **Mp** 329-330 °C. **^{1}H NMR** (400 MHz, Chloroform-*d*) δ 8.21 (d, *J* = 7.7 Hz, 2H), 7.82 – 7.72 (m, 10H), 7.60 (td, *J* = 7.7 1.4 Hz, 2H), 7.56 – 7.47 (m, 4H). **^{19}F NMR** (376 MHz, Chloroform-*d*) δ -139.16 (q, *J* = 30.3 Hz). **MS (MALDI-TOF): *m/z* =** 521 [M^{+}] **UV−Vis** (CH_2Cl_2) λ_{max}/nm (ε/dm^{3}.mol^{-1}.cm^{-1}): 328 (7.6x10^{4}), 441 (9.2x10^{4}). **Fluorescence** (DCM, Excitation at 441 nm): 527 nm.

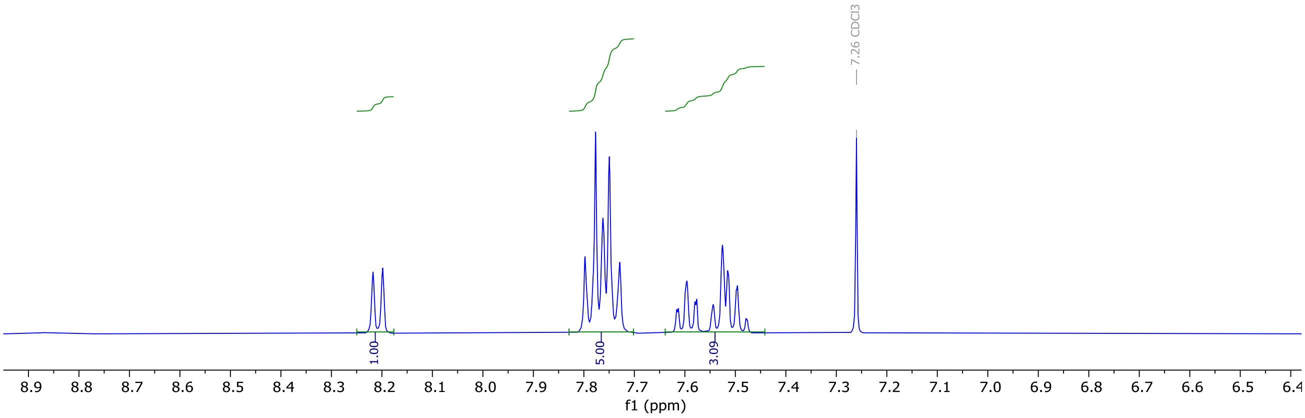

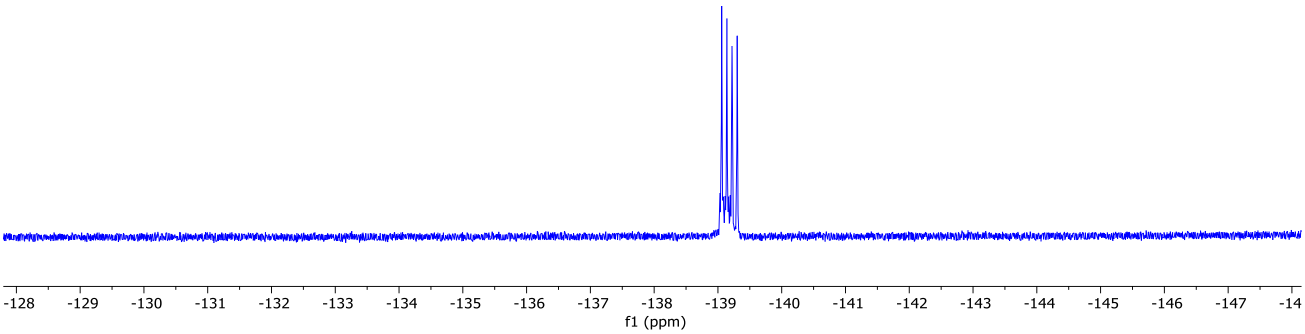

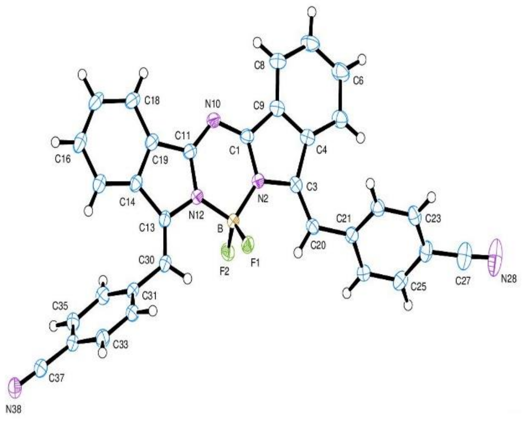

General diphenoxyborylation of aza-dibenzodipyrromethenes

A mixture of aminoisoindoline derivative (1 eq) and triphenyl borate (2 eq) in *p*-xylene (2 mL), were sealed in a microwave vessel with a magnetic bar and then purged and refilled with N_2 three times. The mixture was irradiated in a microwave reactor at 220 ºC for 1 h. After cooling, the solvent was removed under reduced pressure, and the residue was finally purified by column chromatography in silica gel.

Aza-DBBODIPY 5h

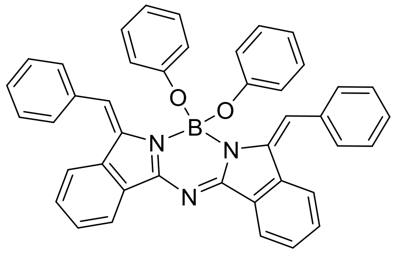

Following the general procedure, aza-DBBODIPY **5H** was isolated by column chromatography (EtOAc:Pet. Ether 2:7) as a yellow solid (80 mg, 73 %). **Mp** 180-182 °C. **^{1}H NMR** (500 MHz, $CDCl_3$) δ 8.36 (s, 2H), 8.15 (d, 2H, J = 7.6 Hz), 7.54 – 7.37 (m, 16H), 7.02 (t, 4H, J = 8.0 Hz), 6.79 – 6.72 (m, 6H). **^{13}C-NMR** (125.7 MHz, $CDCl_3$): δ (ppm) = 164.5, 156.8, 138.4, 136.2, 135.3, 133.0, 132.3, 129.7, 129.4, 129.2, 128.7 (4), 128.7 (0), 127.5, 123.7, 123.6, 120.5, 119.3. **^{11}B-NMR** (160.5 MHz, $CDCl_3$): δ (ppm) = 2.34 (s). **MS (MALDI-TOF): *m/z* =** 620 [M+H$^+$]. **UV−Vis** (CH_2Cl_2) λ_{max}/nm (ε/dm^3.mol^{-1}.cm^{-1}): 319 (2.0x10^4), 451 (1.8x10^4). **Fluorescence** (DCM, Excitation at 450 nm): 548 nm.

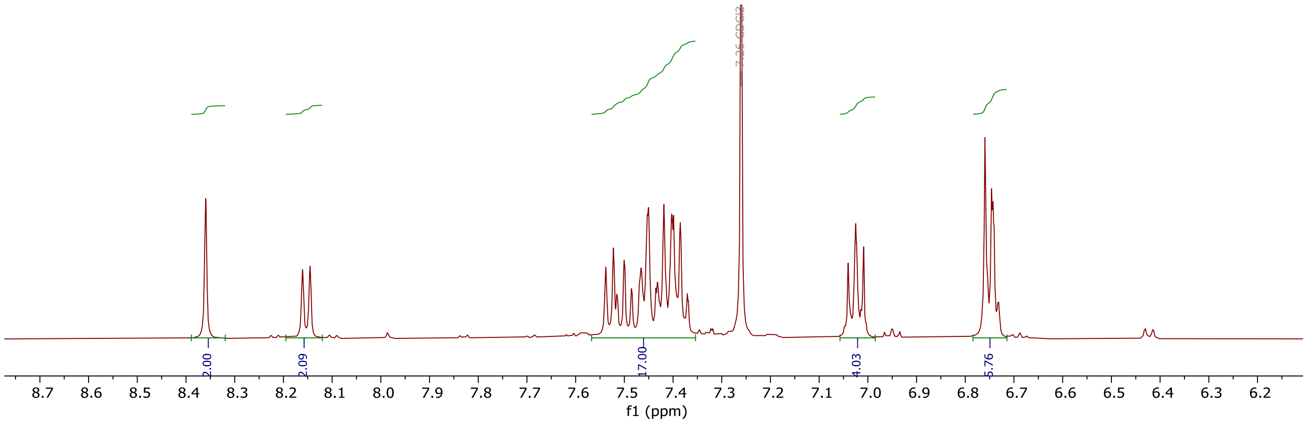

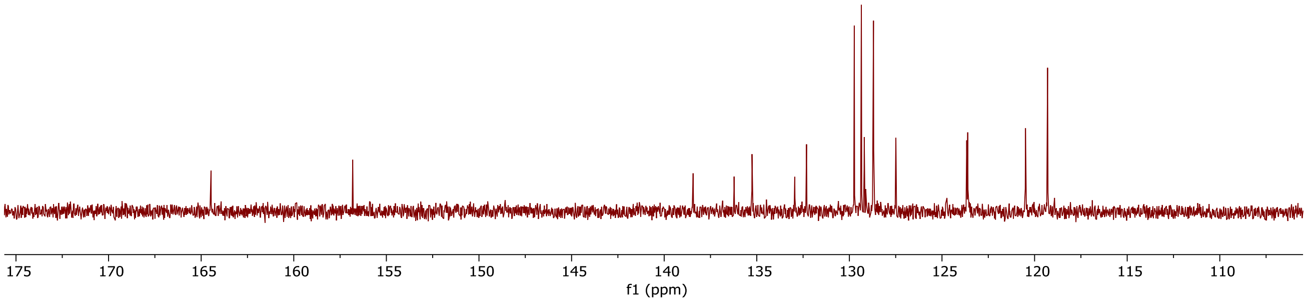

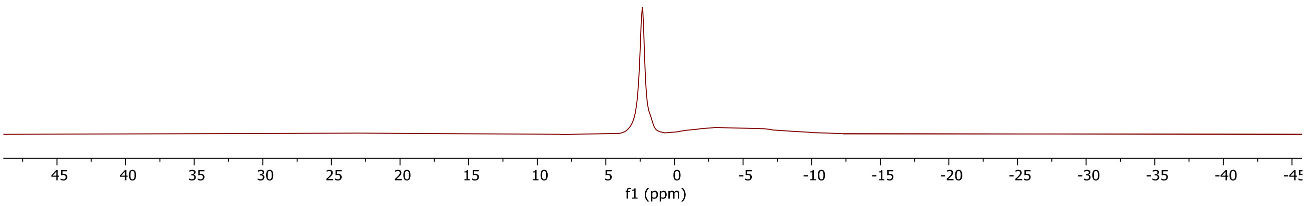

Aza-DBBODIPY 5h

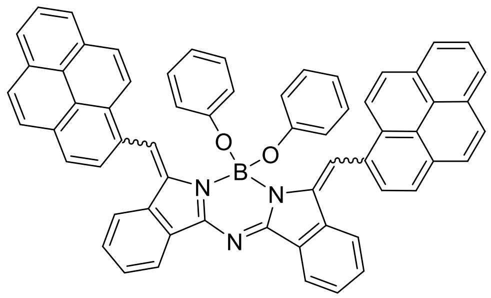

Following the general procedure, aza-DBBODIPY **5h** was isolated by column chromatography (x2, Et_2O:hexane:DCM 1:5:2, then DCM:Pet. Ether 3:2) as a red solid (80 mg, 73 %). Crystals suitable for crystallography were grown from DCM/Methanol. **Mp** 322-325 °C. ^{1}**H NMR** (500 MHz, $CDCl_3$) shows a complex mixture of equilibrating isomers/atropisomers (integration values are normalised to 38H). 9.10 (s, 2H, 1.2), 8.24 – 7.95 (m, ~16.7H), 7.93 – 7.13 (m, 11.5H), 7.07-6.47 (m, 7.7H), 6.20 (2, 0.9H, J = 7.8 Hz). ^{13}**C-NMR** (125.7 MHz, $CDCl_3$): δ (ppm) = 164.7, 157.0, 156.6, 139.6, 136.4, 133.0, 132.4, 131.9, 131.4, 131.3, 131.2, 129.8, 129.7, 129.3, 129.1, 128.3, 128.3, 127.8, 127.5, 126.4, 126.0, 125.8, 125.7, 125.0(1), 125.0(0), 124.8, 124.7, 123.7, 120.7, 120.3, 119.6, 119.3, 118.7. ^{11}**B-NMR** (160.5 MHz, $CDCl_3$, 298 K): δ (ppm) = 2.76 (s, 1B, major), 1.80 (s, 1B, minor), ratio 4:1. **MS (MALDI-TOF):** ***m/z*** **=** 774 [M^+]. **UV−Vis** (DCM) λ_{max}/nm (ε/dm^3.mol^{-1}.cm^{-1}): 346 (2.0x10^4), 490 (1.3x10^4). **Fluorescence** (DCM, Excitation at 490 nm): 626 nm.

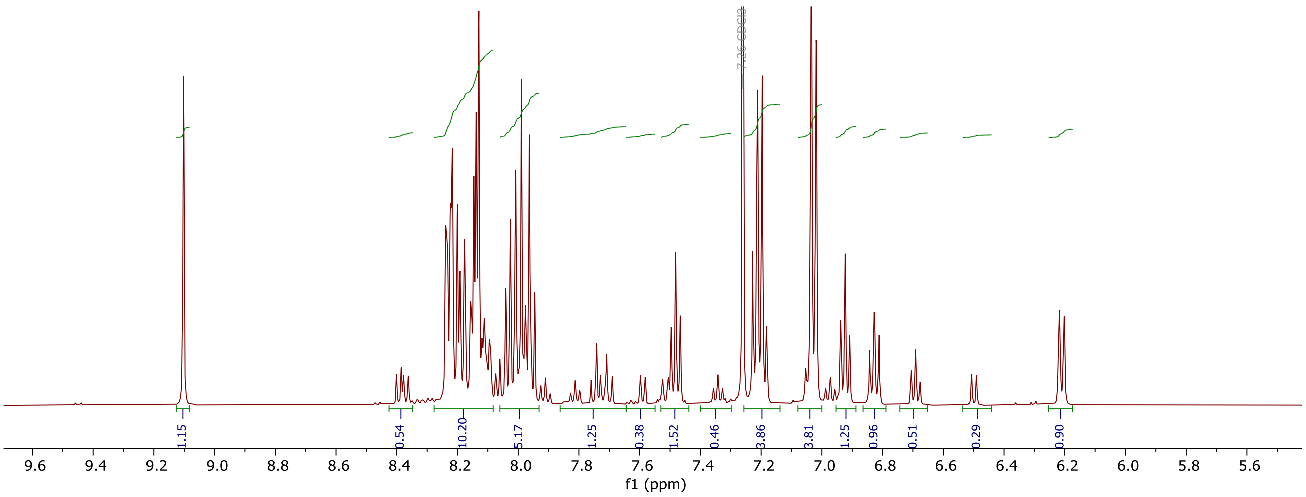

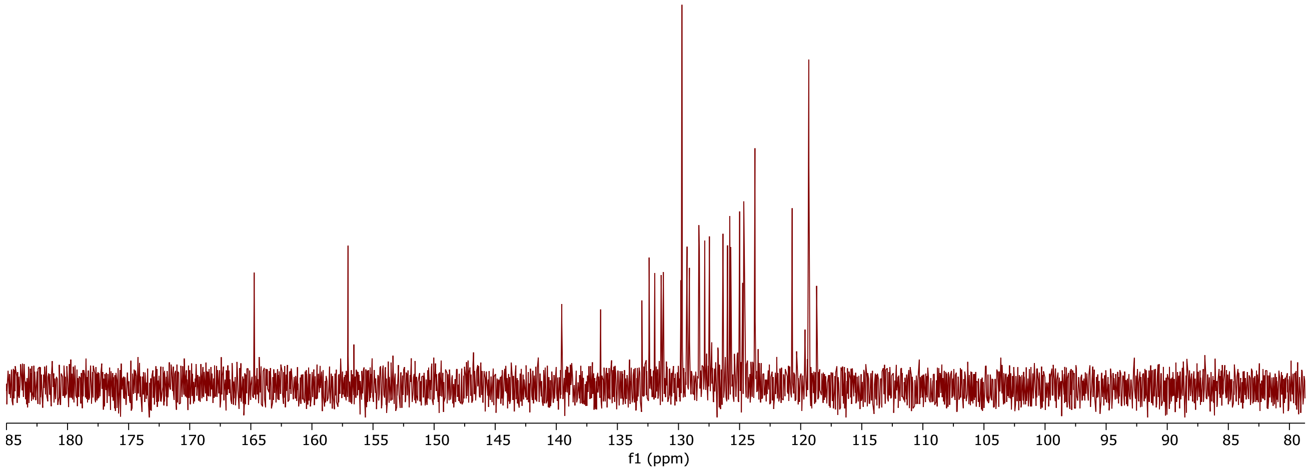
f1 (ppm)

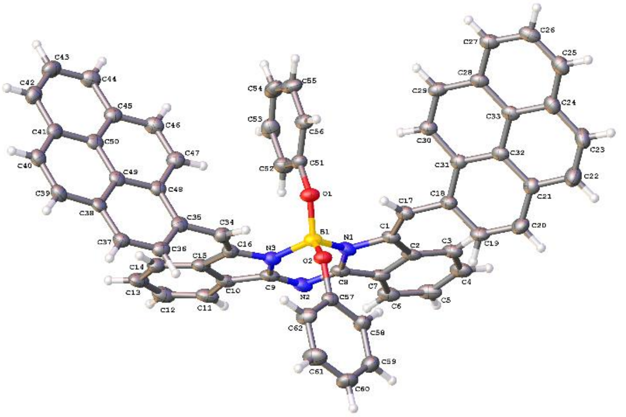

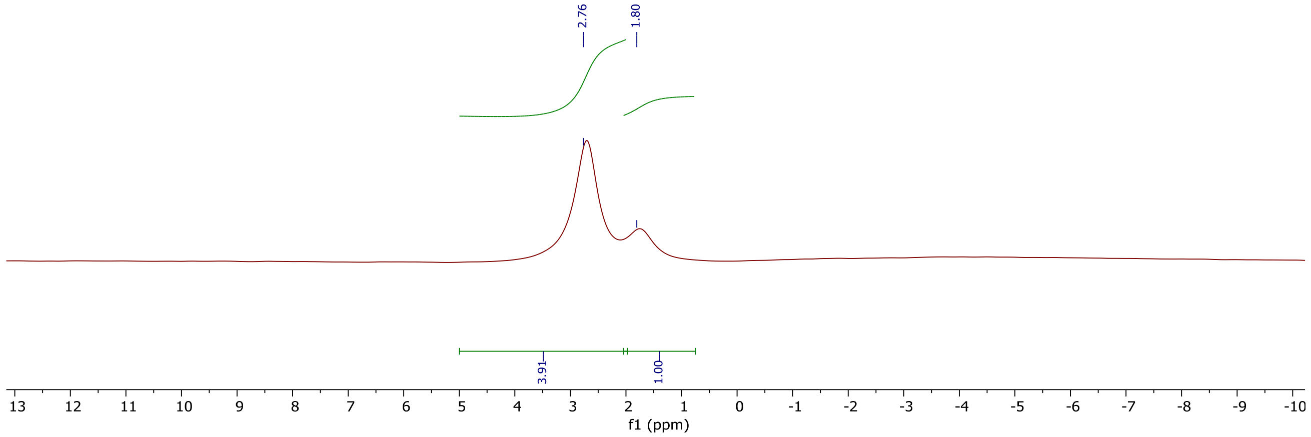
2.76
1.80
3.91
1.00
f1 (ppm)

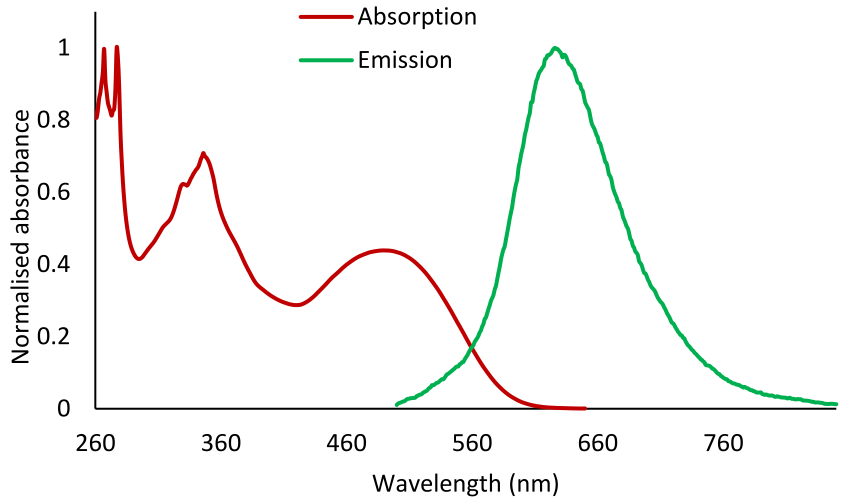

Absorption and emission (λ_{ex} = 490 nm) for **23** (in dichloromethane).

Unsymmetrical Aza-DBDPMs

Synthesis of mixed alkoxophenyl aza-DPM 30

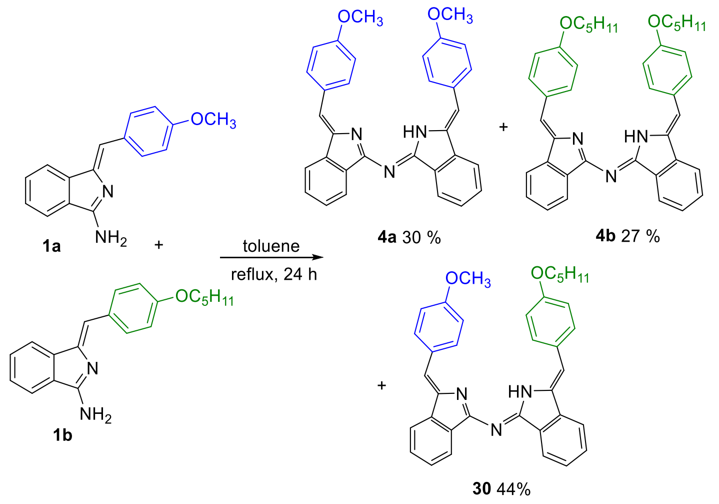

A mixture of aminoisoindolines **1b** (0.200 g, 0.652 mmol, 1 eq) and **1a** (0.195 g, 0.783 mmol, 1.2 eq) was dissolved in toluene (12 ml) and the mixture was refluxed overnight. After evaporating the solvent, the crude mixture was purified by column chromatography using DCM and petroleum ether 3:1. The isolated compounds were recrystallised from 1:1 DCM and methanol to produce compound **4a** (55 mg, 30 %), compound **4b** (53 mg, 27 %), and the desired unsymmetrical dimer **30** (155 mg, 44 %). **MP =** 151-152 °C. **^{1}H NMR** (500 MHz, Chloroform-*d*) δ 8.08 (d, *J* = 7.5 Hz, 2H), 7.88 – 7.83 (m, 4H), 7.80 (d, *J* = 7.5, Hz, 2H), 7.53 (td, *J* = 7.4, 1.2 Hz, 2H), 7.48 (td, *J* = 7.4, 1.2 Hz, 2H), 6.78 (2x s, 2H), 6.64-6.60 (m, 4H), 3.79 (t, *J* = 6.7 Hz, 2H), 3.69 (s, 3H), 1.75 (pen, *J* = 6.8 Hz, 2H), 1.50 – 1.36 (m, 4H), 0.95 (t, *J* = 7.1 Hz, 3H). **^{13}C NMR** (126 MHz, Chloroform-*d*) δ 159.27, 158.94, 140.23, 139.99, 139.86, 139.80, 131.32, 130.13, 130.11, 128.47, 128.23, 128.05, 122.61, 119.30, 115.26, 114.75, 67.94, 55.13, 29.10, 28.33, 22.64, 14.19. **MS (MALDI-TOF):** ***m/z*** **=** 539 [M,100 %]. **UV−Vis** (CH_2Cl_2) λ_{max}/nm (ε/dm^3.mol^{-1}.cm^{-1}): 360 (9.7x10^4), 500 (1.9x10^4).

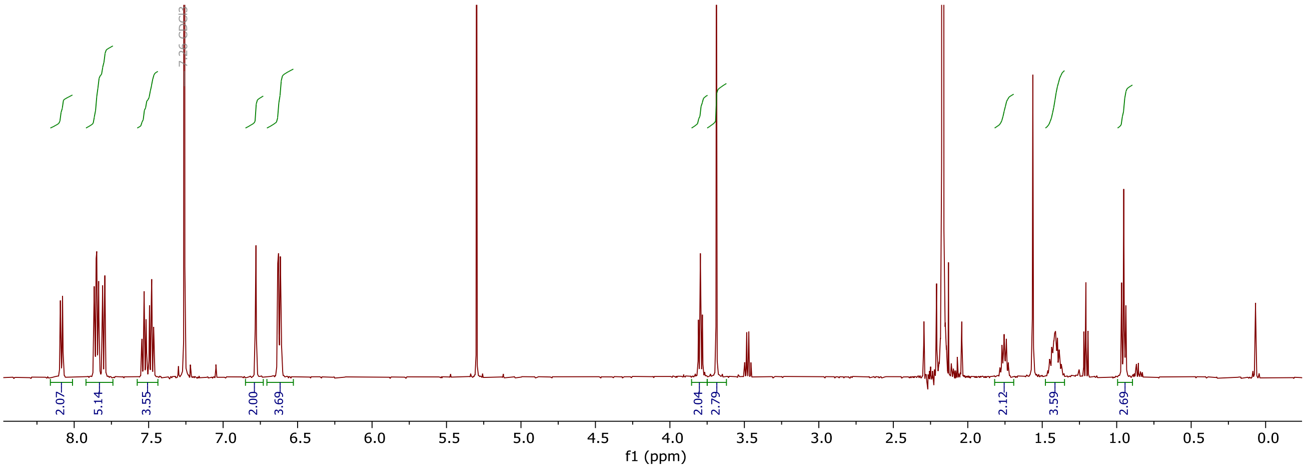

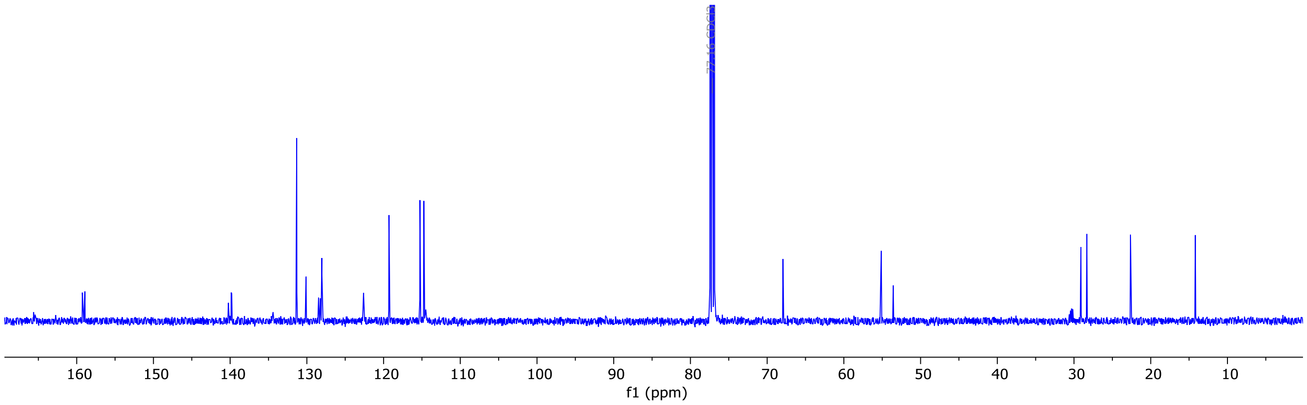

Mixed condensation/dimerization between aminoisoindolines 18 and 1c to give unsymmetrical aza-DBDPM 32

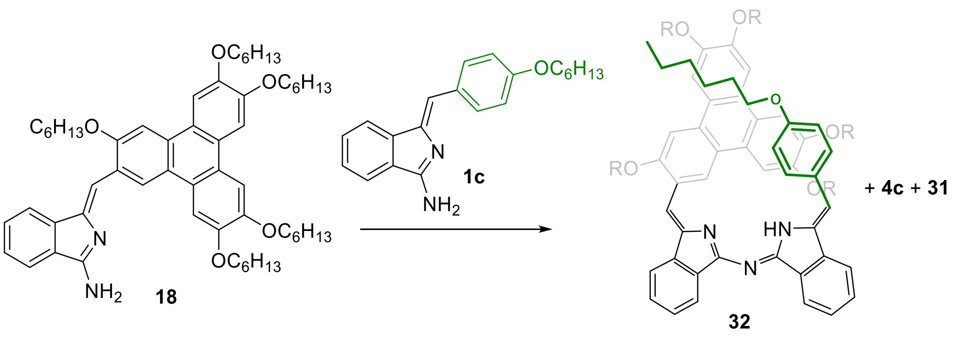

A mixture of aminoisoindolines **18** (0.10 g, 0.11 mmol, 1 eq) and **1c** (0.10 g, 0.31 mmol, 2.8 eq) was dissolved in toluene (12 ml) and the mixture was refluxed overnight. After evaporating the solvent, the crude mixture was separated by column chromatography using DCM and petroleum ether 1:4. Reprecipitation from DCM/EtOH gave pure unsymmetrical aza-DBDPM **32** (28 mg, 22 %). **MP =** 161-163°C. **^{1}H NMR** (500 MHz, Acetone-d_6) δ 13.38 (s, 1H,NH), 9.50 (s, 1H), 8.13 (d, J = 7.5 Hz, 1H), 8.08 (dd, J = 8.4, 7.5 Hz, 2H), 8.03 – 7.95 (m, 4H), 7.91 (s, 1H), 7.88 (s, 1H), 7.70 (td, J = 7.4, 1.1 Hz, 1H), 7.62 (tdd, J = 7.4, 2.6, 1.1 Hz, 2H), 7.57 (td, J = 7.4, 1.0 Hz, 1H), 7.55 (d, J = 8.8 Hz, 2H), 7.45 (s, 1H), 6.90 (s, 1H), 5.87 (d, J = 8.7 Hz, 2H), 4.34 (t, J = 6.5 Hz, 2H), 4.28 (dt, J = 12.8, 6.4 Hz, 4H), 4.21 (t, J = 6.4 Hz, 2H), 3.79 (t, J = 6.5 Hz, 2H), 2.93 (t, J = 6.8 Hz, 2H), 1.91 –1.53 (m, 12H), 1.50 – 1.39 (m,12H), 1.38 – 1.03 (m, 24H), 1.00 – 0.83 (m, 18H). **^{13}C NMR** (126 MHz, Chloroform-d & THF) δ 157.23, 154.47, 149.24, 148.60, 147.92, 147.66, 139.45, 129.51, 125.27, 124.54, 123.13, 122.87, 121.99, 118.53, 118.24, 112.66, 106.86, 106.57, 105.64, 104.58, 103.41, 83.12, 68.77, 68.26, 68.05, 67.71, 65.91, 49.97, 41.16, 30.88, 30.84, 29.02, 28.77, 28.72, 28.67, 28.41, 28.35, 28.13, 21.76, 16.28, 12.80, 8.00. **MS (MALDI-TOF): *m/z* =** 1175 [M+H]. **UV−Vis** (CH_2Cl_2) λ_{max}/nm (ε/dm^3.mol^{-1}.cm^{-1}): 371 (8.6x10^4), 403 (shoulder), 525 (1.6x10^4).

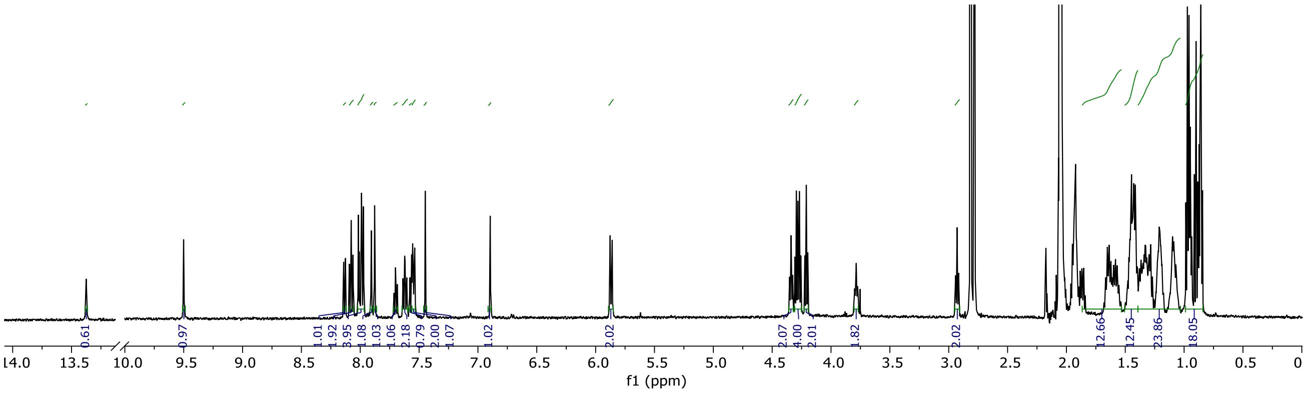

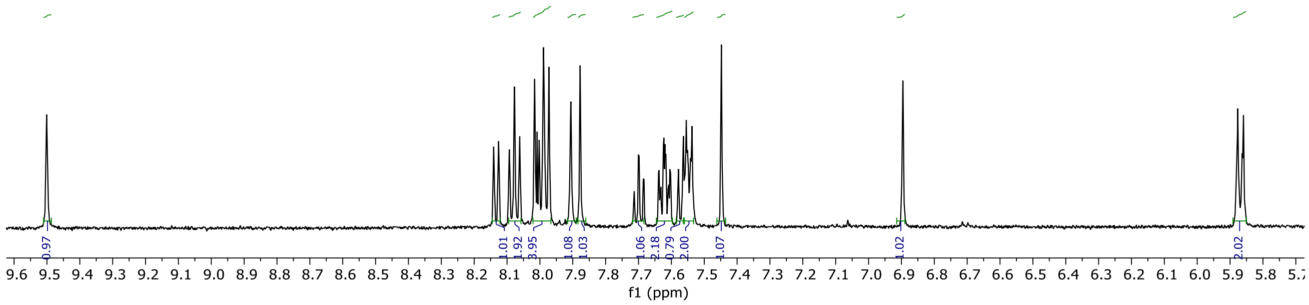

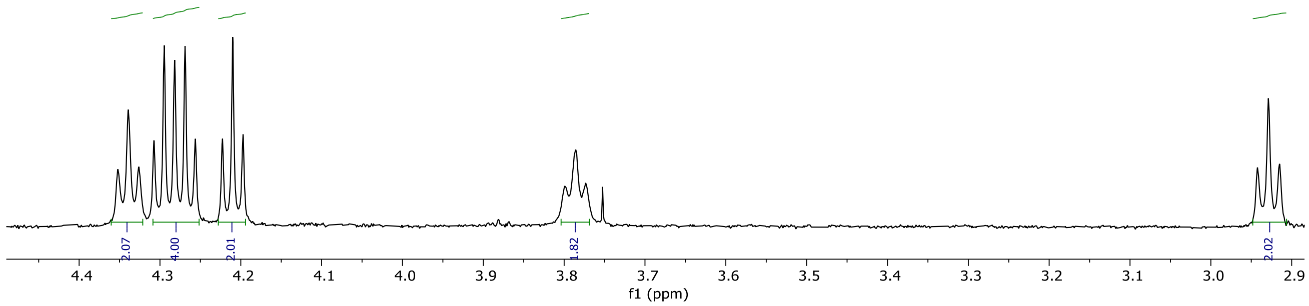

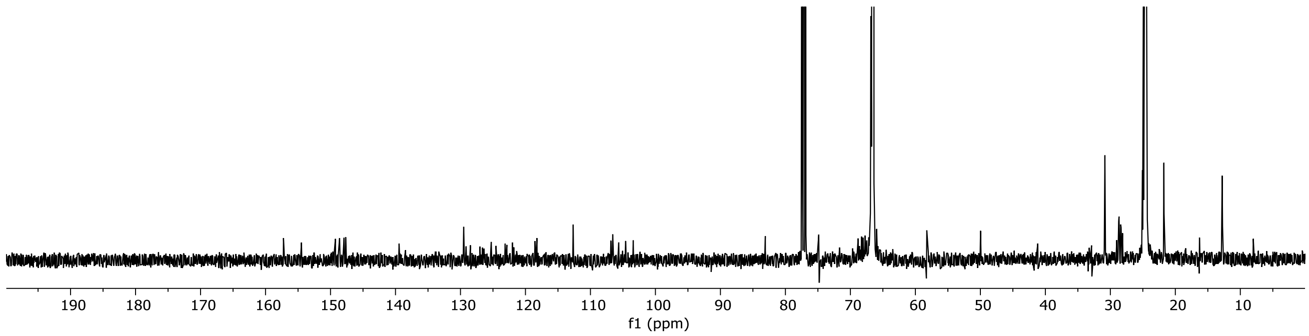

Mixed condensation/dimerization between aminoisoindolines 18 and 17 to give unsymmetrical aza-DBDPM 33

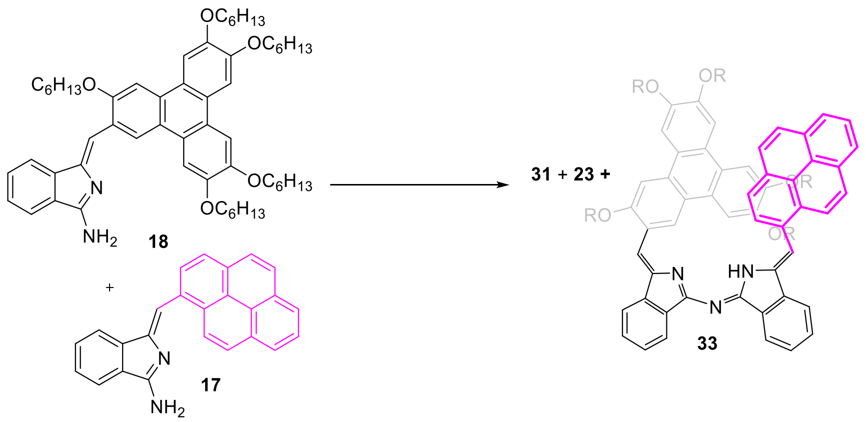

A mixture of aminoisoindolines **18** (0.10 g, 0.11 mmol, 1 eq) and **17** (0.06 g, 0.17 mmol, 1.5 eq) was dissolved in toluene (2 ml) and the mixture was refluxed overnight. After evaporating the solvent, the crude mixture was separated by column chromatography using EtOAc and petroleum ether 1:4. Recrystallisation from DCM/EtOH gave pure unsymmetrical aza-DBDPM **33** (42 mg, 32 %). **MP** = 185-187°C. **^{1}H NMR** (500 MHz, THF-d_8) δ 14.71 (s, 1H), 9.53 (s, 1H), 8.94 (d, *J* = 8.1

Hz, 1H), 8.23 (d, *J* = 8.8 Hz, 1H), 8.20 (d, *J* = 7.8 Hz, 1H), 8.14 – 8.09 (m, 3H), 7.96 (d, *J* = 7.5 Hz, 1H), 7.90 – 7.86 (m, 2H), 7.83 (d, *J* = 7.5 Hz, 1H), 7.80 (s, 1H), 7.76 (s, 1H), 7.71 (s, 1H), 7.66 – 7.51 (m, 4H), 7.49 (s, 1H), 7.42 (s, 1H), 7.19 (d, *J* = 8.8 Hz, 1H), 6.96 (d, *J* = 8.5 Hz, 1H), 6.92 (d, *J* = 8.5 Hz, 1H), 6.39 (s, 1H), 6.26 (s, 1H), 4.42 (br t, 2H), 4.26 (t, *J* = 6.2 Hz, 2H), 3.89 (t, *J* = 6.3 Hz, 2H), 3.78 (t, *J* = 6.8 Hz, 2H), 3.68 (t, *J* = 6.8 Hz, 2H), 2.12 – 1.10 (m, 40H), 1.09 (t, *J* = 6.8 Hz, 3H), 1.05 – 0.98 (m, 9H), 0.86 (t, *J* = 6.8 Hz, 3H). **^{13}C NMR** (126 MHz, Chloroform-*d* + THF, data assignments are tentative because a high quality spectrum could not be obtained due to aggregation) δ 155.57, 149.82, 148.51, 148.31, 147.87, 142.69, 141.17, 139.98, 139.78, 131.08, 130.12, 129.15, 128.01, 127.22, 126.95, 126.80, 126.09, 125.26, 125.16, 124.99, 124.55, 124.38, 122.72, 122.47, 122.31, 121.61, 120.48, 119.57, 108.24, 104.89, 104.35, 104.09, 84.13, 51.01, 32.04, 32.00, 31.95, 30.06, 29.94, 29.69, 29.51, 26.29, 26.26, 26.23, 22.92, 22.71, 21.68, 13.90, 13.84, 9.07. **MS (MALDI-TOF): *m/z* =** 1199 [M+H]. **UV−Vis** (CH_2Cl_2) λ_{max}/nm (ε/$dm^3.mol^{-1}.cm^{-1}$): 400 ($3.7x10^4$), 455 (shoulder), 550 ($0.2x10^4$).

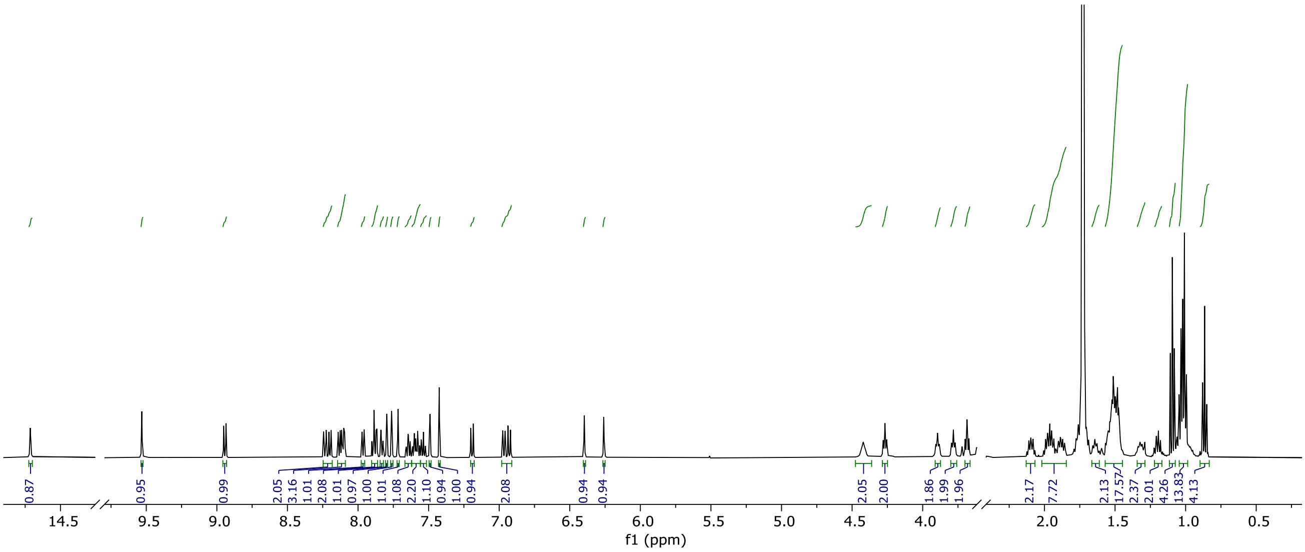

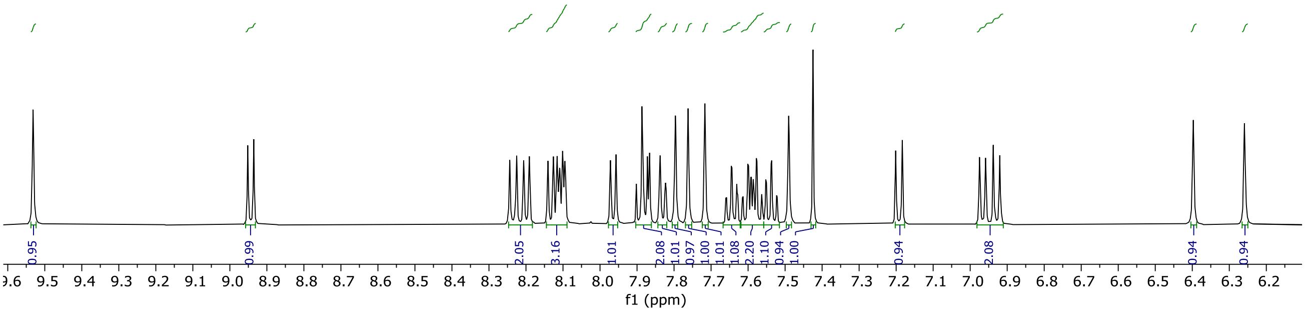

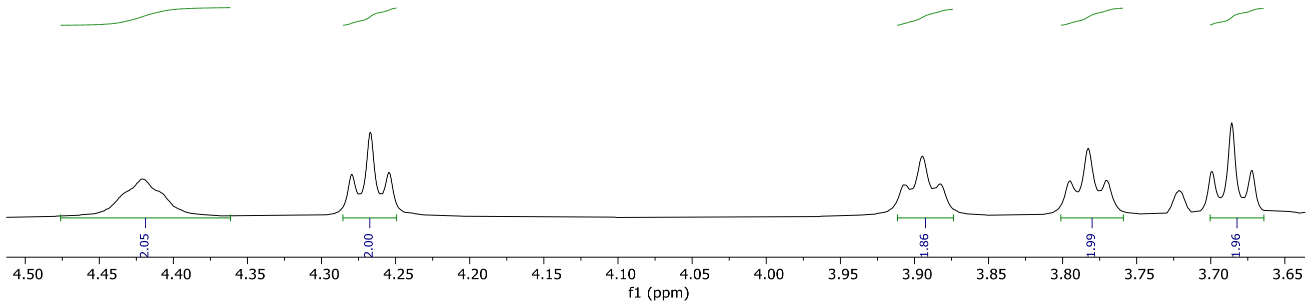
2.05
2.00
1.86
1.99
1.96
4.50 4.45 4.40 4.35 4.30 4.25 4.20 4.15 4.10 4.05 4.00 3.95 3.90 3.85 3.80 3.75 3.70 3.65
f1 (ppm)

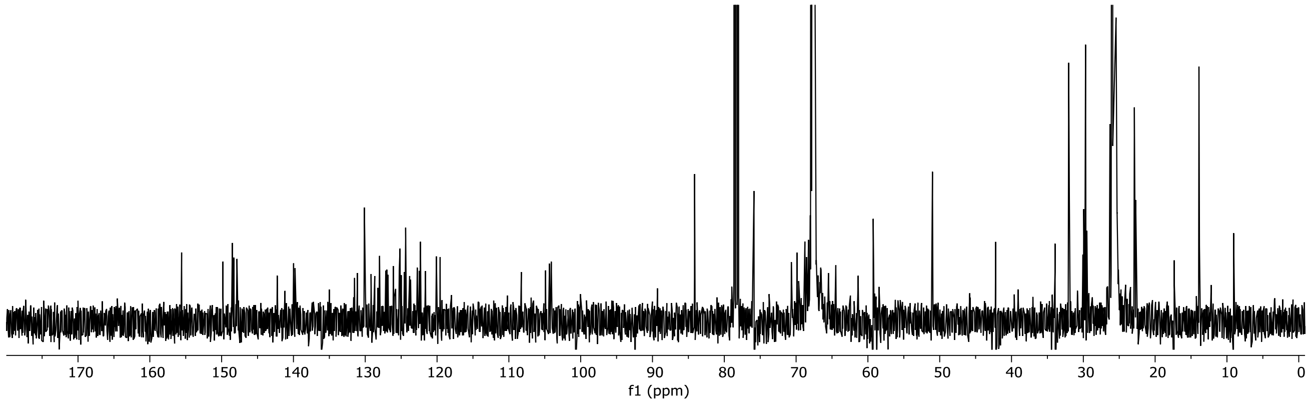
170 160 150 140 130 120 110 100 90 80 70 60 50 40 30 20 10 0
f1 (ppm)

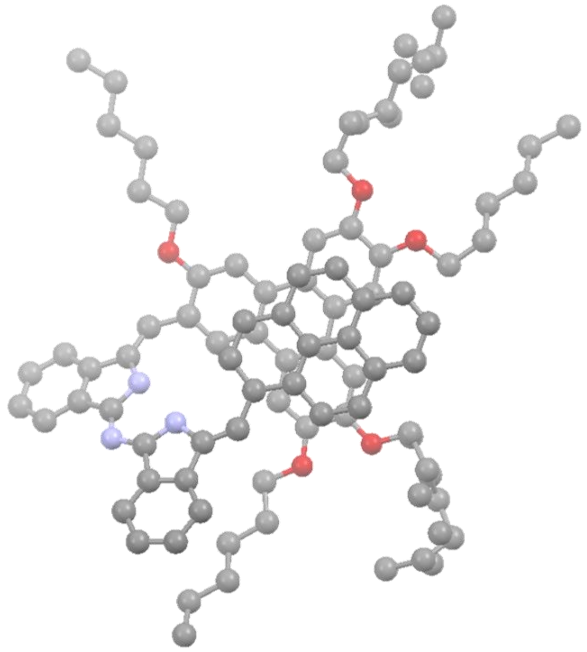

Aminoisoindoline tosylate 35a

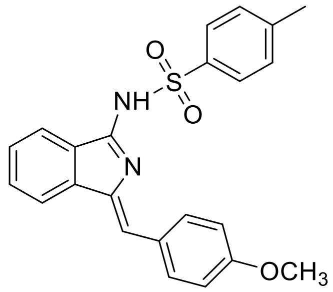

Aminoisoindoline **1a** (0.25 g, 1.0 mmol, 1 eq) was stirred with *p*-toluene sulfonyl chloride (0.21 g, 1.1 mmol, 1.1 eq) in dry DCM (6 ml) and triethylamine (0.3 ml) at room temperature. The reaction was monitored by TLC until completion (overnight) then diluted with water (20 mL), extracted with DCM (2 x 25 mL), dried over $MgSO_4$ and filtered. The solvent was removed and the residue recrystallised from DCM:PE to give tosylate **35a** as yellow crystals (380 mg, 94 %). **MP =** 196-197 °C. **^{1}H NMR** (500 MHz, Chloroform-*d*) δ 10.58 (s, 1H), 7.91 (dd, *J* = 8.2, 2.8 Hz, 3H), 7.77 (d, *J* = 7.8 Hz, 1H), 7.63 (t, *J* = 7.6 Hz, 1H), 7.47 (dd, *J* = 8.2, 6.0 Hz, 3H), 7.29 (d, *J* = 8.0 Hz, 2H), 7.05 (d, *J* = 8.3 Hz, 2H), 6.69 (s, 1H), 3.88 (s, 3H), 2.40 (s, 3H). **^{13}C NMR** (126 MHz, Chloroform-*d*) δ 159.87, 159.25, 143.17, 139.15, 136.43, 132.68, 132.65, 130.73, 130.14, 129.44, 128.92, 126.76, 126.60, 124.05, 119.58, 115.18, 109.95, 55.45, 21.56. **MS (MALDI-TOF): *m/z*** = 404 [M].

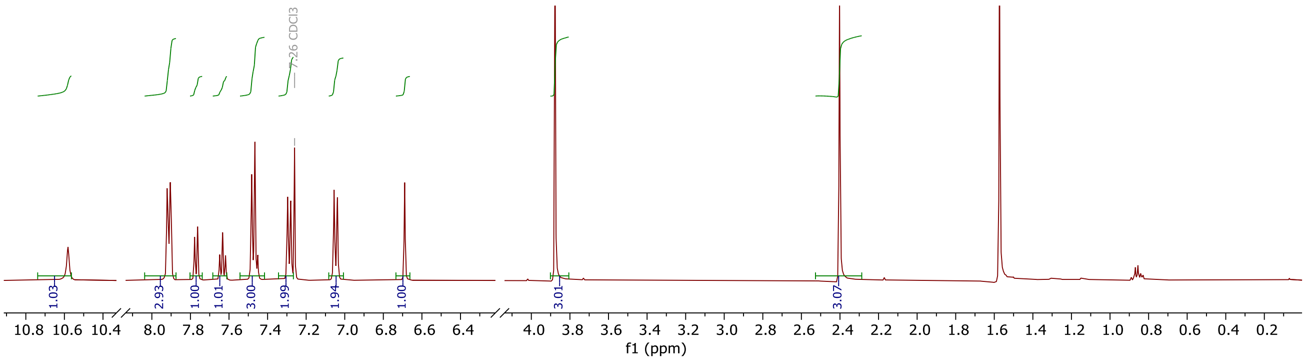

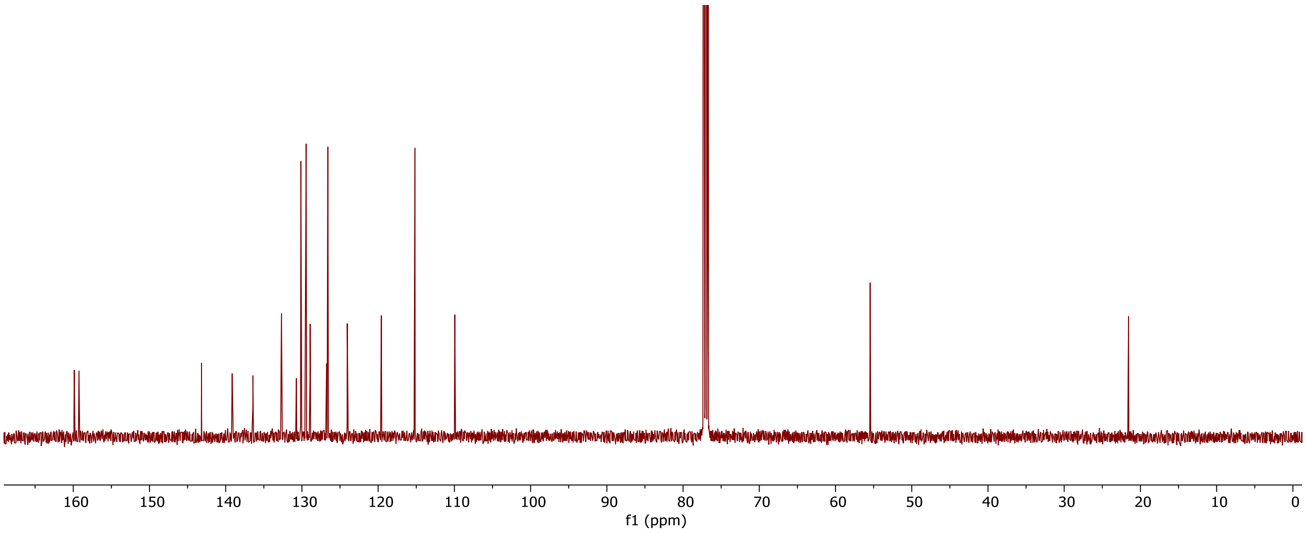

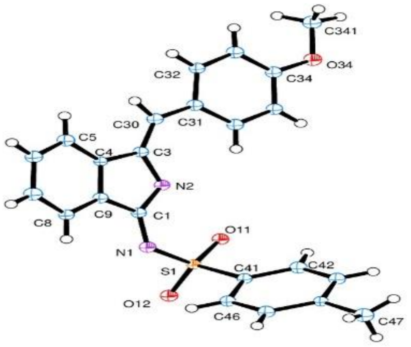

Aminoisoindoline tosylate 35b

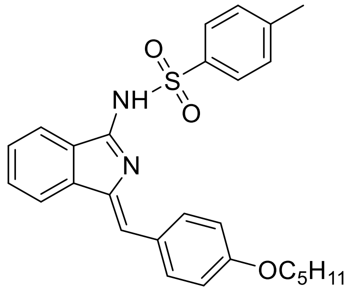

Synthesized as described above from aminoisoindoline **1b** to give tosylate **35b** as yellow crystals (345 mg, 92 %). **MP =** 105-106 °C. **^{1}H NMR** (500 MHz, Chloroform-*d*) δ 10.58 (s, 1H), 7.91 (dd, *J* = 7.5, 5.5 Hz, 3H), 7.76 (d, *J* = 8.0 Hz, 1H), 7.63 (td, *J* = 7.6, 1.1 Hz, 1H), 7.46 (dd, *J* = 8.2, 5.5 Hz, 3H), 7.29 (d, *J* = 8.1 Hz, 2H), 7.03 (d, *J* = 8.0 Hz, 2H), 6.68 (s, 1H), 4.02 (t, *J* = 6.6 Hz, 2H), 2.40 (S, 1H), 1.89-1.79 (m, 2H), 1.49 – 1.38 (m, 4H), 0.95 (t, *J* = 7.1 Hz, 3H). **^{13}C NMR** (126 MHz, Chloroform-*d*) δ 159.50, 159.24, 143.16, 139.17, 136.46, 132.67, 132.48, 130.67, 130.14, 129.44, 128.86, 126.60, 126.49, 124.03, 119.57, 115.68, 110.15, 68.23, 28.90, 28.19, 22.47, 21.55, 14.04. **MS (MALDI-TOF):** ***m/z*** **=** 460 [M].

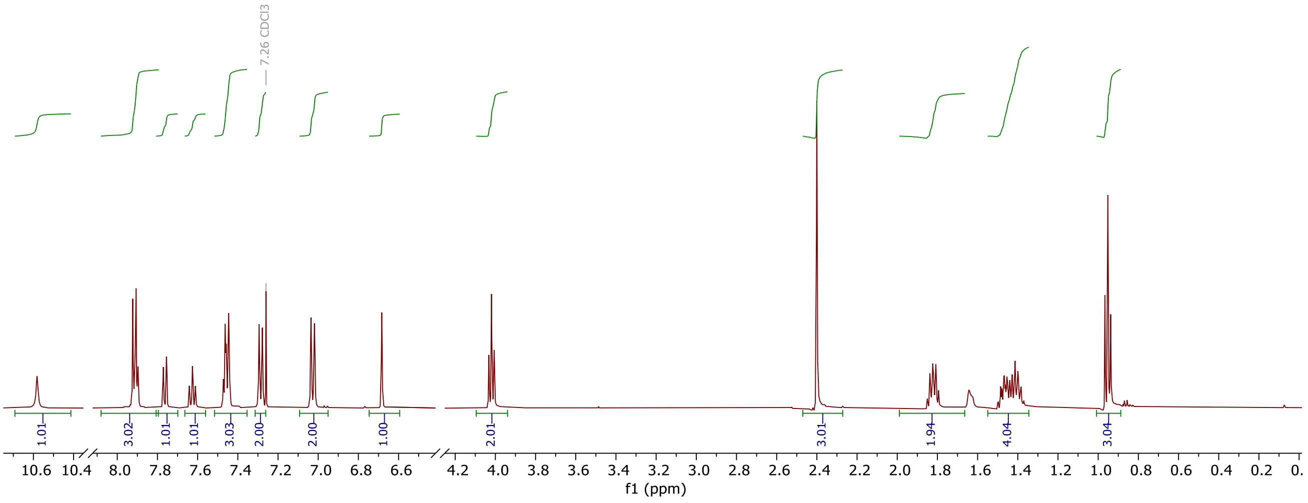

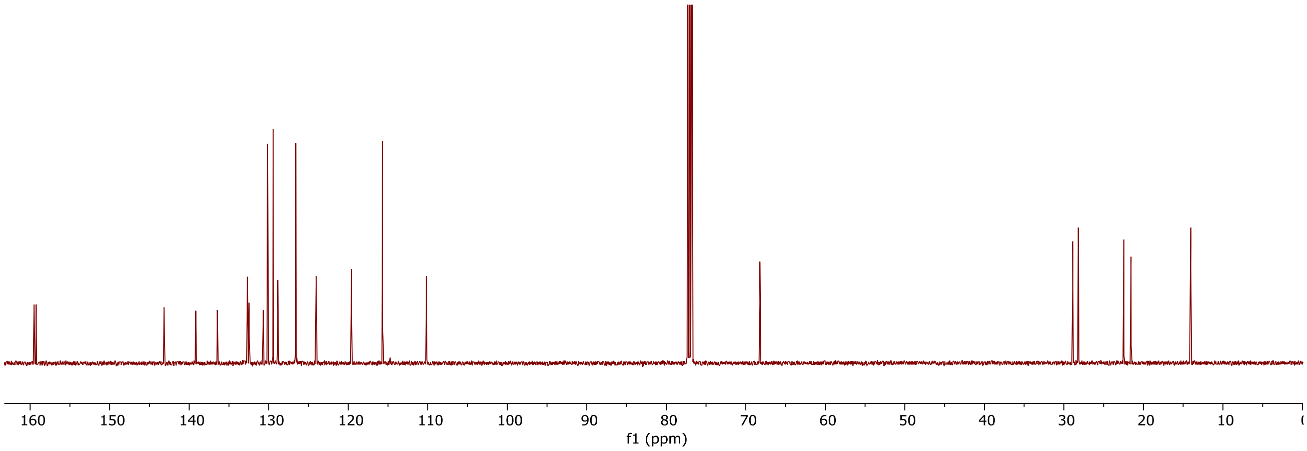

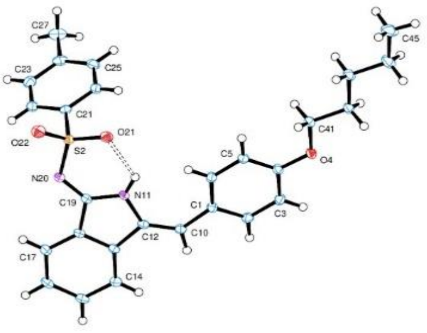

Aminoisoindoline triflate 36a

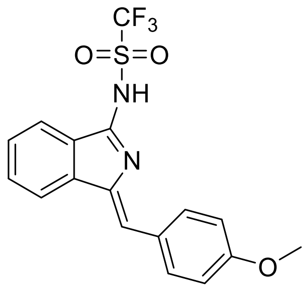

Aminoisoindoline **1a** (0.25 g, 1.0 mmol, 1 eq) was stirred in dry DCM (30 ml) and pyridine (0.1g, 1.2 mmol, 1.2 eq) -20 ^{o}C under nitrogen. Trifluoromethanesulfonic acid anhydride (0.33 g, 1.2 mmol, 1.2 eq) was added over 30 min. The reaction was stirred at room temperature overnight then diluted with water (60 mL), extracted with DCM (3 x 15 ml). The solvent was evaporated and the residue purified by column chromatography (DCM:Pet. Ether) and recrystallised from DCM/Pet. Ether to give triflate **35a** (280 mg, 74 %). **MP =** 150-151 °C. **^{1}H NMR** (400 MHz, Chloroform-*d*) δ 10.48 (s, 1H), 8.00 (d, *J* = 7.8 Hz, 1H), 7.84 (d, *J* = 7.8 Hz, 1H), 7.74 (t, *J* = 7.8 Hz, 1H), 7.56 (t, *J* = 7.8 Hz, 1H), 7.43 (d, *J* = 8.0 Hz, 2H), 7.04 (d, *J* = 8.0 Hz, 2H), 6.92 (s, 1H), 3.87 (s, 3H). **^{13}C NMR** (101 MHz, Chloroform-*d*) δ 163.10, 160.70, 136.37, 133.92, 131.68, 130.60, 129.56, 129.44, 125.77, 124.58, 119.85, 119.8 (t, J = 319 Hz), 115.45, 114.66, 55.50. **MS (MALDI-TOF): m/z** 382 [M].

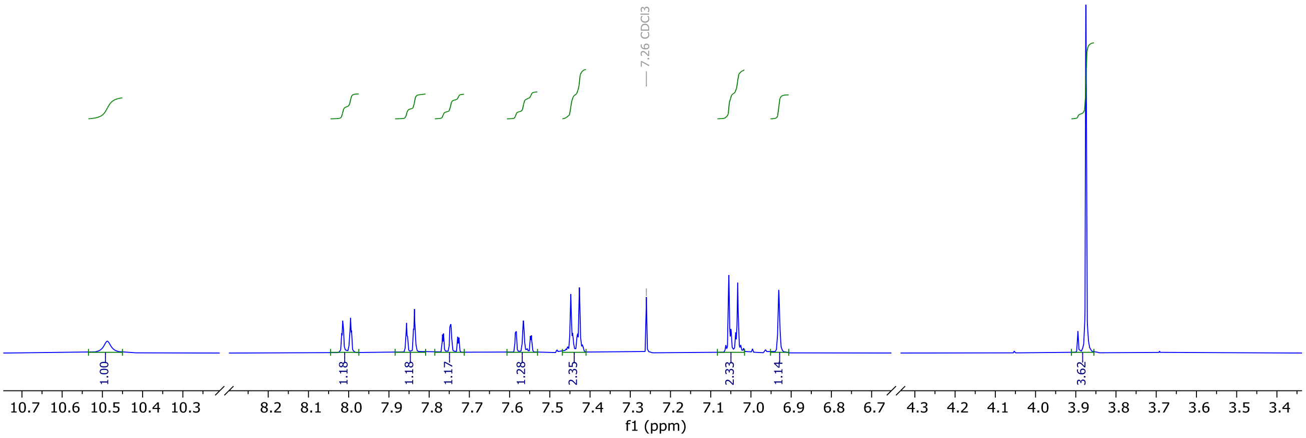

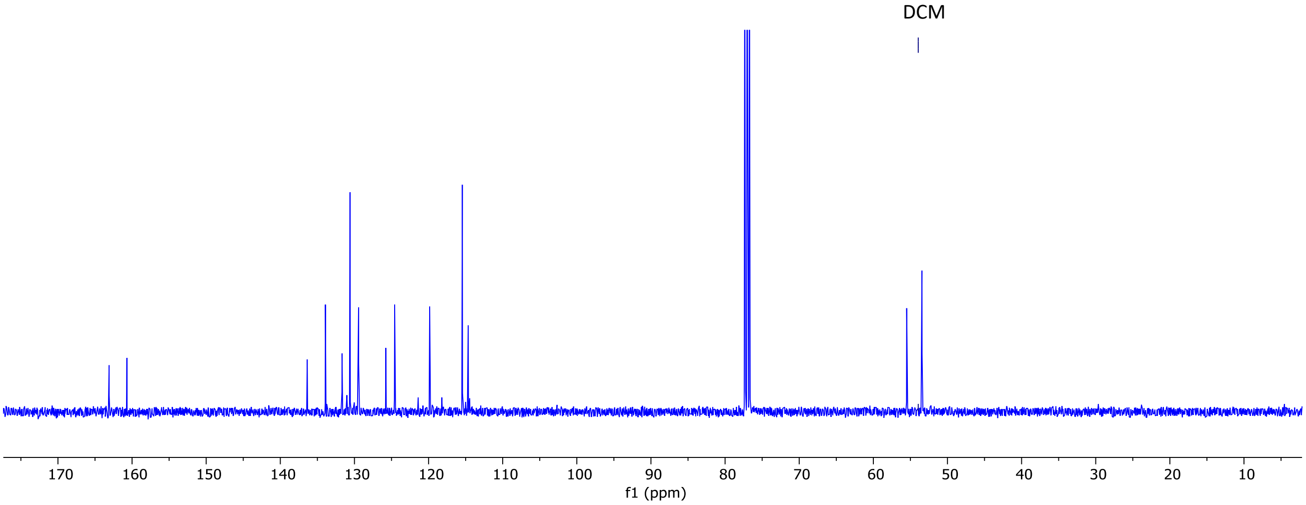

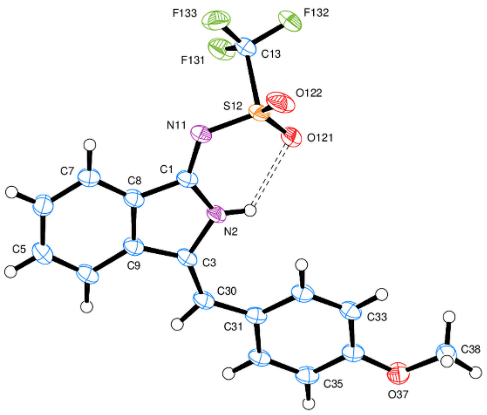

Synthesis of unsymmetrical aza-DBDPM 34a from tosylate 35a (or triflate 36a) and aminoisoindoline 1f

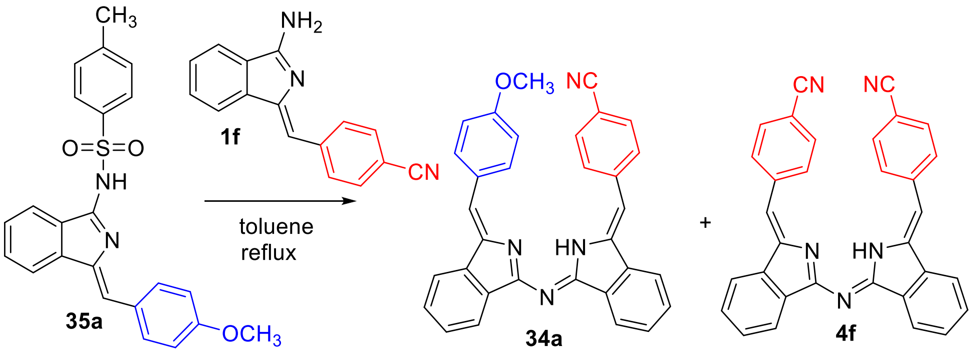

A mixture of triflate **35a** (0.082 g, 0.203 mmol, 1 eq) and aminoisoindoline **1f** (0.050 g, 0.203 mmol, 1 eq) was dissolved in toluene (4 ml) and refluxed overnight. After evaporating the solvents, the crude mixture was separated by column chromatography using DCM and petroleum ether 3:1. The isolated compounds were recrystallised from DCM-methanol to give **4f** (12 mg, 24 %), and the desired unsymmetrical aza-DBDPM **34a** (49 mg, 50 %).

Under identical conditions, replacing tosylate **35a** with triflate **36a** led to isolation of **4f** (10 mg, 20 %), and the desired unsymmetrical aza-DBDPM **34a** (63 mg, 64 %).

MP = 219-220 °C. **IR** (thin film cm $^{-1}$) 2225. **^{1}H NMR** (500 MHz, Acetone-d_6) δ 13.20 (s, 2H), 8.14 (d, *J* = 8.2 Hz, 2H), 8.09 – 8.01 (m, 4H), 7.87 (d, *J* = 8.2 Hz, 2H), 7.69 – 7.56 (m, 4H), 7.37 (d, *J* = 8.2 Hz, 2H), 7.13 (s, 1H), 7.10 (s, 1H), 6.69 (d, *J* = 8.3 Hz, 2H), 3.80 (s, 3H). **^{13}C NMR** (126 MHz, Acetone-d_6 +DCM) 141.76, 133.35, 132.08, 131.71, 141.36, 130.98, 129.91, 123.04, 122.85, 121.15, 120.80, 115.70, 115.53,113.53, 111.16, 56.67. **MS (MALDI-TOF):** ***m/z*** **=** 478 [M]. **UV−Vis** (CH_2Cl_2) λ_{max}/nm (ε/dm^3.mol^{-1}.cm^{-1}): 362 (6.8x10^4), 495 (1.0x10^4).

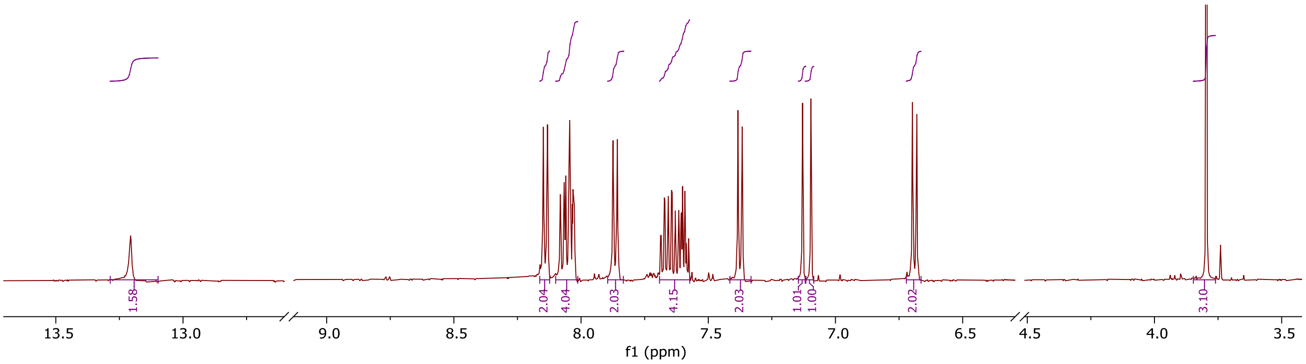

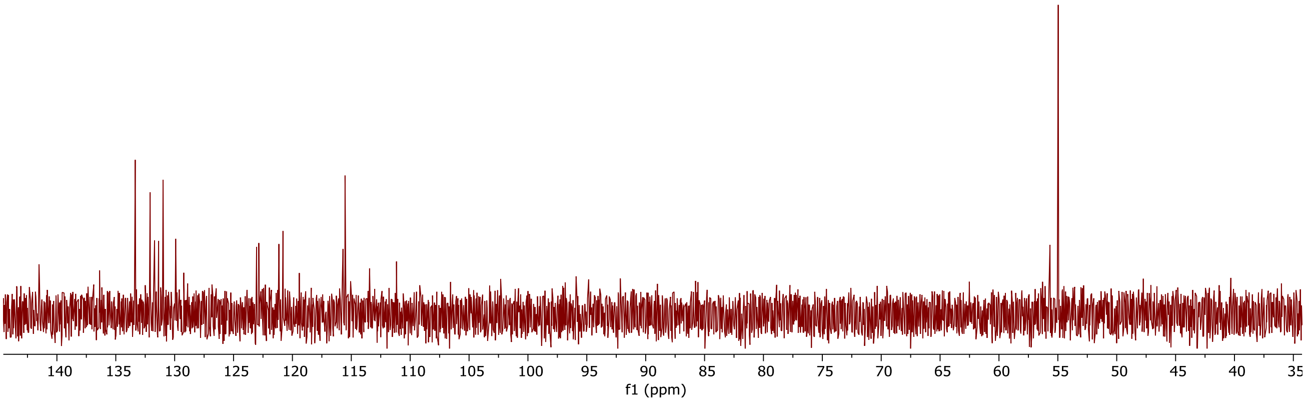

Synthesis of unsymmetrical aza-DBDPM 34b from tosylate 33b and aminoisoindoline 1f

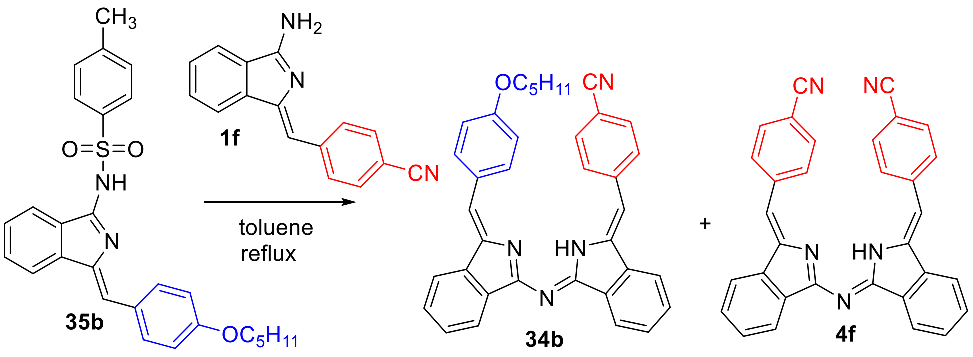

Prepared following the above procedure used for synthesis of **34a** to give **4f** (11 mg, 23 %), and the desired unsymmetrical aza-DBDPM **34** (54 mg, 49 %). **MP** =197-198 °C. **IR** (thin film cm $^{-1}$) 2220. **^{1}H NMR** (500 MHz, Acetone-d_6, major isomer) δ 13.21 (s, 1H), 8.15 (d, *J* = 8.3 Hz, 2H), 8.09– 8.01 (m, 4H), 7.86 (d, *J* = 8.1 Hz, 2H), 7.72 – 7.56 (m, 4H), 7.37

(d, *J* = 8.3 Hz, 2H), 7.13 (s, 1H), 7.10 (s, 1H), 6.69 (d, *J* = 8.3 Hz, 2H), 3.94 (t, *J* = 6.6 Hz, 2H), 1.86 – 1.77 (m, 2H), 1.52 – 1.38 (m, 4H), 0.96 (t, *J* = 7.3 Hz, 3H). **^{13}C NMR** (126 MHz, Acetone-d_6) 133.31. 132.10, 130.98, 129.90, 129.21, 123.05, 122.85, 121.15, 120.80, 115.96 68.69, 28.94, 23.22, 14.35. **MS (MALDI-TOF): *m/z*** = 534 [M]. **UV−Vis** (CH_2Cl_2) λ_{max}/nm (ε/dm^3.mol^{-1}.cm^{-1}): 363 (8.7x10^4), 508 (1.2x10^4).

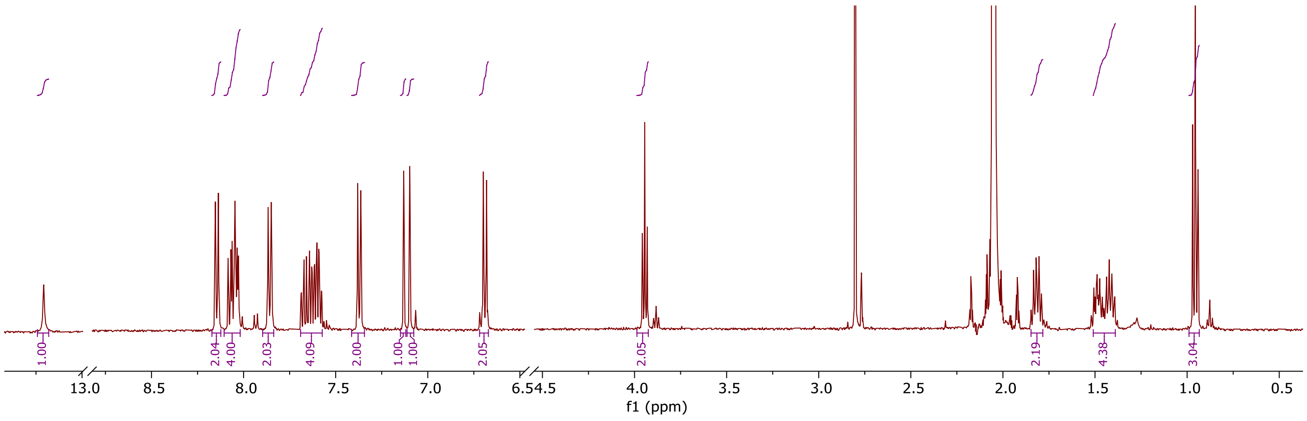

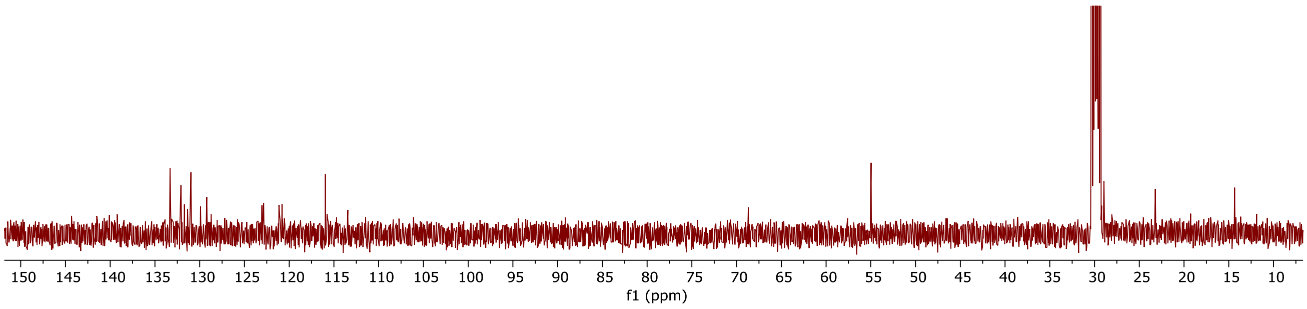

Aza-DBBODIPY 37

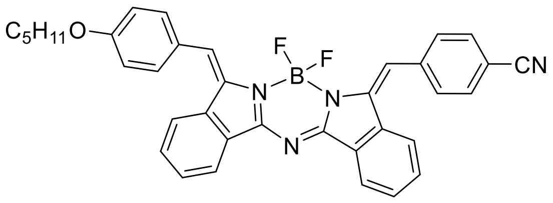

Following the general procedure, aza-DBBODIPY **37** was isolated as an orange crystalline solid (77 mg, 70 %). **MP** = 234-235 °C. **IR** (thin film cm $^{-1}$) 2223. **^{1}H NMR** (500 MHz, Methylene Chloride-d_2) δ 8.21 (d, *J* = 7.7 Hz, 2H), 7.88 (d, *J* = 7.8 Hz, 1H), 7.86 (s, 1H), 7.82 – 7.70 (m, 4H), 7.62 – 7.46 (m, 7H), 7.01 (d, *J* = 8.6 Hz, 2H), 4.05 (t, *J* = 6.6 Hz, 2H), 1.87 – 1.80 (m, 2H), 1.52 – 1.38 (m, 4H), 0.95 (t, *J* = 7.2 Hz, 3H). **^{13}C NMR** (126 MHz, Methylene Chloride-d_2) δ 160.57, 140.35, 136.42, 132.89, 132.85, 131.85, 130.77, 130.42, 129.67, 127.09, 124.17, 123.95, 123.79, 123.67, 119.03, 115.07, 112.43, 68.66, 29.35, 28.61, 22.89, 14.22. **^{19}F NMR** (376 MHz, Methylene Chloride-d_2) δ -138.99 (q, *J* = 30.03 Hz). **MS (MALDI-TOF): *m/z*** = 582 [M]. **UV−Vis** (CH_2Cl_2) λ_{max}/nm (ε/dm^3.mol^{-1}.cm^{-1}): 326 (7.5x10^4), 465 (5.8x10^4). **Fluorescence** (DCM, Excitation at 465 nm): 567 nm.

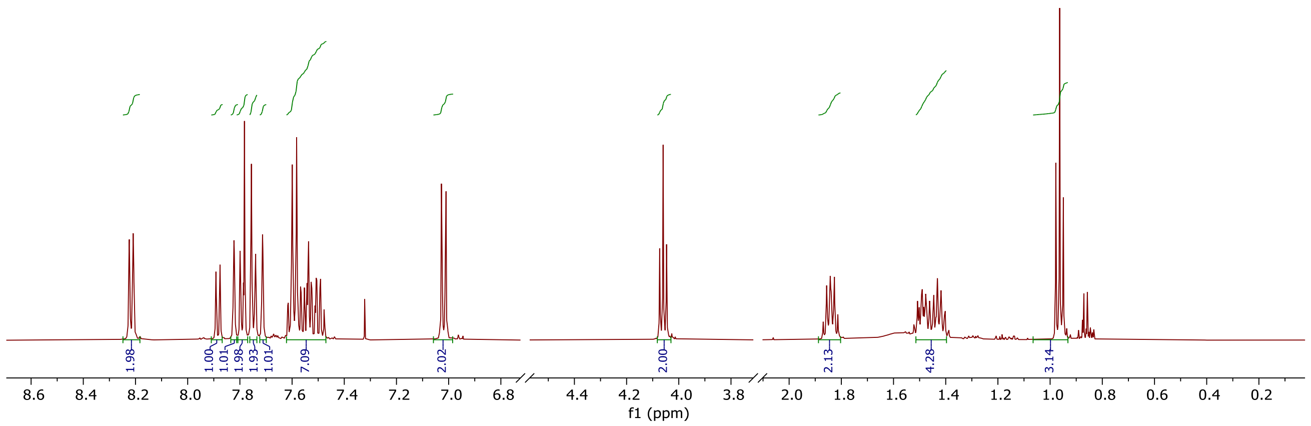

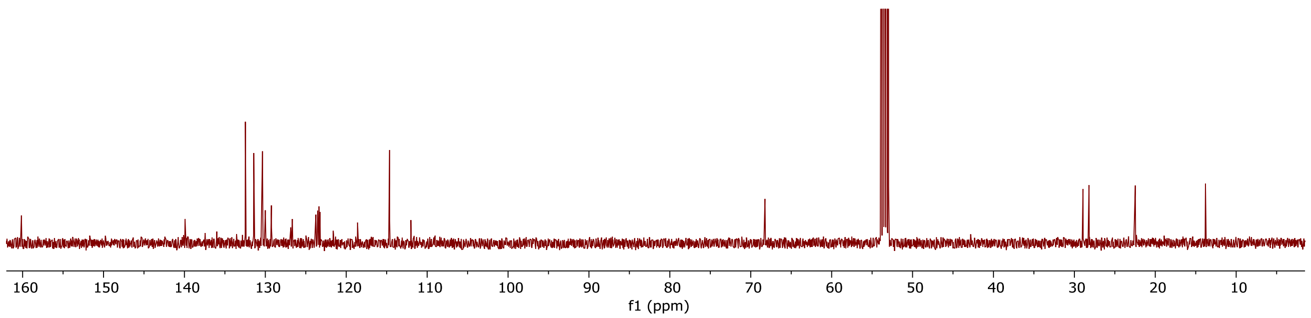

Synthesis of unsymmetrical aza-DBDPM 30 from trifate 36a and aminoisoindoline 1b

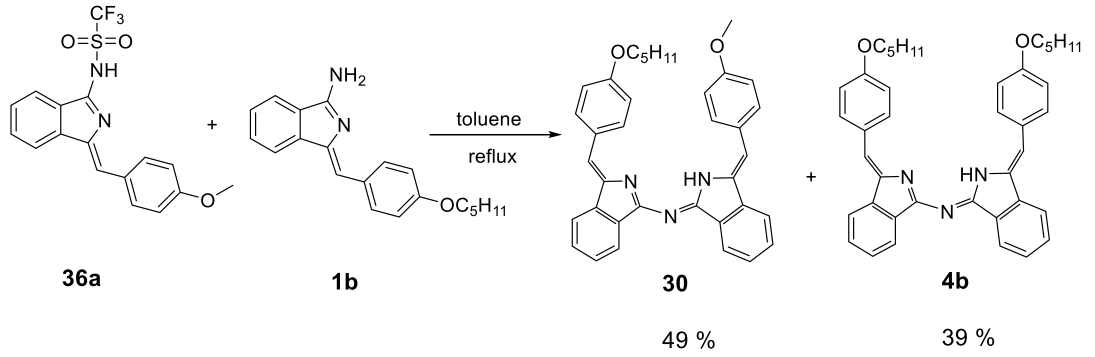

A mixture of triflate **36a** (0.060 g, 0.156 mmol, 1 eq) and aminoisoindoline **1b** (0.062 g, 0.202 mmol, 1.3 eq) was dissolved in toluene (4 ml) and refluxed overnight. After evaporating the solvents, the crude mixture was purified by column chromatography using DCM/petroleum ether 3:1. The isolated compounds were recrystallised from DCM and methanol to give **4b** (23 mg, 39 %), and the desired unsymmetrical aza-DBDPM **30** (42 mg, 49 %).

Crystallographic Details

Compound 4d

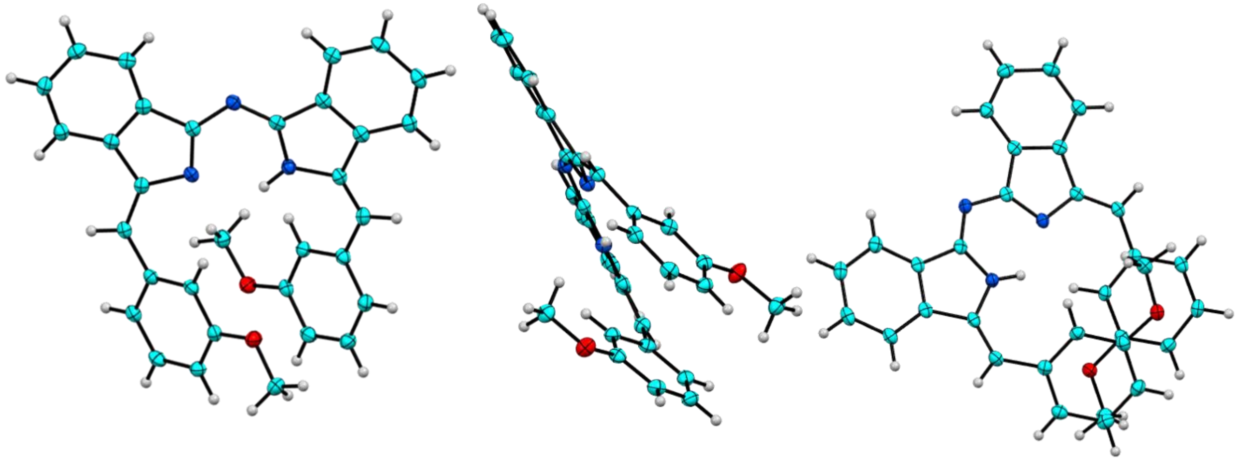

Crystal data and structure refinement for $C_{16}H_{12}NO-N=C_{16}H_{13}NO$

Identification code	budur5
Elemental formula	C32 H25 N3 O2
Formula weight	483.55
Crystal system, space group	Triclinic, P -1 (no. 2)
Unit cell dimensions	a = 7.47981(11) Å α = 101.5042(12) °
	b = 11.99860(18) Å β = 95.5867(11) °
	c = 14.03558(19) Å γ = 103.8877(13) °
Volume	1184.22(3) $Å^3$
Z, Calculated density	2, 1.356 Mg/m^3
F(000)	508
Absorption coefficient	0.679 mm^{-1}
Temperature	100.01(10) K
Wavelength	1.54184 Å
Crystal colour, shape	deep red plate
Crystal size	0.185 x 0.14 x 0.04 mm
Crystal mounting:	on a small loop, in oil, fixed in cold N_2 stream
On the diffractometer:	
Theta range for data collection	3.251 to 69.994 °

Limiting indices	-8<=h<=9, -14<=k<=14, -17<=l<=17
Completeness to theta = 67.684	99.6 %
Absorption correction	Semi-empirical from equivalents
Max. and min. transmission	1.00000 and 0.78261
Reflections collected (not including absences)	35660
No. of unique reflections	4473 [R(int) for equivalents = 0.045]
No. of 'observed' reflections ($I > 2\sigma_I$)	4170
Structure determined by:	dual methods, in SHELXT
Refinement:	Full-matrix least-squares on F^2, in SHELXL
Data / restraints / parameters	4473 / 0 / 340
Goodness-of-fit on F^2	1.057
Final R indices ('observed' data)	$R_1 = 0.045$, $wR_2 = 0.102$
Final R indices (all data)	$R_1 = 0.047$, $wR_2 = 0.103$
Reflections weighted: $w = [\sigma^2(Fo^2)+(0.0380P)^2+0.8695P]^{-1}$ where $P=(Fo^2+2Fc^2)/3$	
Extinction coefficient	n/a
Largest diff. peak and hole	0.25 and -0.23 e.Å^{-3}
Location of largest difference peak	near C(29)-C(23) bond

Crystal structure analysis of 4d

Crystal data: $C_{32}H_{25}N_3O_2$, M = 483.55. Triclinic, space group P-1 (no. 2), a = 7.47981(11), b = 11.99860(18), c = 14.03558(19) Å, α = 101.5042(12), β = 95.5867(11), γ = 103.8877(13) °, V = 1184.22(3) Å^3. Z = 2, Dc = 1.356 g cm^{-3}, F(000) = 508, T = 100.01(10) K, μ(Cu-Kα) = 6.8 cm^{-1}, λ(Cu-Kα) = 1.54184 Å.

The crystals were deep red plates. One, *ca* 0.04 x 0.14 x 0.185 mm, was mounted in oil on a small loop and fixed in the cold nitrogen stream on a Rigaku Oxford Diffraction XtaLAB Synergy diffractometer, equipped with Cu-Kα radiation, HyPix detector and mirror monochromator. Intensity data were measured by thin-slice ω-scans. Total no. of reflections recorded, to θ_{max} = 70.0°, was 35660 of which 4473 were unique (Rint = 0.045); 4170 were 'observed' with $I > 2\sigma_I$.

Data were processed using the CrysAlisPro-CCD and -RED (Rigaku Oxford Diffraction Ltd., Abingdon, UK (2018)) programs. The structure was determined by the intrinsic phasing routines in the SHELXT program[33] and refined by full-matrix least-squares methods, on F^2's, in SHELXL.[34] The non-hydrogen atoms were refined with anisotropic thermal parameters. The hydrogen atom on N(22) was located in a difference map and was refined freely. The remaining hydrogen atoms were included in idealised positions and their Uiso values were set to ride on the Ueq values of the parent carbon atoms. At the conclusion of the refinement, wR_2 = 0.103 and R_1 = 0.047 (2B) for all 4473 reflections weighted $w = [\sigma^2(F_o^2) + (0.0380\ P)^2 + 0.8695\ P]^{-1}$ with $P = (F_o^2 + 2F_c^2)/3$.

In the final difference map, the highest peak (*ca* 0.25 eÅ^{-3}) was near the C(29)-C(23) bond.

Notes on the structure

The molecule shows pseudo-twofold symmetry about N(1); the major distortions from real symmetry are (1) in the hydrogen bond in N(22)-H(22)…N(2) and (2) in the alignment of the methoxy groups.

The hydrogen atom of the hydrogen bond was located clearly in a difference map and was refined freely, isotropically and satisfactorily. The bond distances and angles in the chain between N(2) and N(22) are in agreement with the arrangement of single- and double-bonds of the formula.

The two bicyclic C_8N moieties are essentially coplanar, with the CHC_6H_4 groups veering away to opposite sides of the plane. There are rotations about the C(10)-C(11) and C(30)-C(31) bonds of about 28° which allow the two phenyl rings to lie almost parallel with C(13) and C(33) overlaid at a distance of 3.562 Å. The O-Me bonds are diametrically opposed, with O(13)-C(131) pointing away from N(1) and O(33)-C(331) towards the N(1) centre.

Molecules are stacked in columns parallel to the *a* axis, each molecule involved with three π…π columns; the central bis-bicyclic rings have centrosymmetrically groups on each side at a distance of *ca* 3.5 Å, and each of the C_6H_4OMe groups has, on one side, the other C_6H_4OMe group of the same molecule and, on the other side, the second C_6H_4OMe group of the next molecule along the *a* axis.

Compound 4f

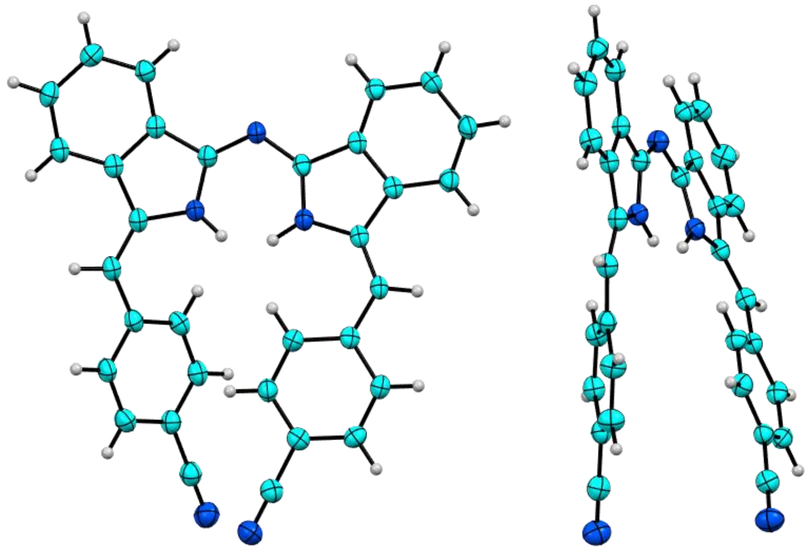

Crystal data and structure refinement for N(C_8NH_5-CH-C_6H_4-CN)(C_8NH_4-CH-C_6H_4-CN)

Identification code	budur11
Elemental formula	C32 H19 N5
Formula weight	473.52
Crystal system, space group	Monoclinic, $P2_1/n$ (eqiv. to no. 14)
Unit cell dimensions	a = 12.5809(2) Å α = 90 °<br>b = 8.0343(2) Å β = 95.576(2) °<br>c = 23.6089(4) Å γ = 90 °
Volume	2375.07(8) $Å^3$
Z, Calculated density	4, 1.324 Mg/m^3
F(000)	984
Absorption coefficient	0.632 mm^{-1}
Temperature	100.01(10) K
Wavelength	1.54184 Å
Crystal colour, shape	dark red needle
Crystal size	0.3 x 0.1 x 0.05 mm
Crystal mounting:	on a Micro-mount, in oil, fixed in cold N_2 stream
On the diffractometer:	
Theta range for data collection	7.688 to 72.478 °

Limiting indices	-15<=h<=13, -9<=k<=9, -29<=l<=27
Completeness to theta = 67.684	99.4 %
Absorption correction	Semi-empirical from equivalents
Max. and min. transmission	1.00000 and 0.81773
Reflections collected (not including absences)	17294
No. of unique reflections	4624 [R(int) for equivalents = 0.032]
No. of 'observed' reflections ($I > 2\sigma_I$)	4049
Structure determined by:	dual methods, in SHELXT
Refinement:	Full-matrix least-squares on F^2, in SHELXL
Data / restraints / parameters	4624 / 0 / 343
Goodness-of-fit on F^2	1.044
Final R indices ('observed' data)	R_1 = 0.037, wR_2 = 0.091
Final R indices (all data)	R_1 = 0.043, wR_2 = 0.094
Reflections weighted: $w = [\sigma^2(Fo^2)+(0.0436P)^2+0.5908P]^{-1}$ where $P=(Fo^2+2Fc^2)/3$	
Extinction coefficient	n/a
Largest diff. peak and hole	0.17 and -0.18 e.Å^{-3}
Location of largest difference peak	near midpoint of C(1)-C(9) bond

Crystal structure analysis of 4f

Crystal data: $C_{32}H_{19}N_5$, M = 473.52. Monoclinic, space group $P2_1/n$ (equiv. to no. 14), a = 12.5809(2), b = 8.0343(2), c = 23.6089(4) Å, β = 95.576(2) °, V = 2375.07(8) $Å^3$. Z = 4, Dc = 1.324 g cm^{-3}, F(000) = 984, T = 100.01(10) K, μ(Cu-Kα) = 6.32 cm^{-1}, λ(Cu-Kα) = 1.54184 Å.

The crystal was a dark red needle. From a sample under oil, one, *ca* 0.05 x 0.1 x 0.3 mm, was mounted on a small loop and fixed in the cold nitrogen stream on a Rigaku Oxford Diffraction XtaLAB Synergy diffractometer, equipped with Cu-Kα radiation, HyPix detector and mirror monochromator. Intensity data were measured by thin-slice ω-scans. Total no. of reflections recorded, to θ_{max} = 72.5°, was 17294 of which 4624 were unique (Rint = 0.032); 4049 were 'observed' with I > $2\sigma_I$.

Data were processed using the CrysAlisPro-CCD and -RED (Rigaku Oxford Diffraction Ltd., Abingdon, UK (2018)) programs. The structure was determined by the intrinsic phasing routines in the SHELXT program[33] and refined by full-matrix least-squares methods, on F^2's, in SHELXL.[34] The non-hydrogen atoms were refined with anisotropic thermal parameters. The amino hydrogen atom was disordered over the two nitrogen atoms, N(2) and N(22), located in difference maps and both were refined freely. The remaining hydrogen atoms were included in idealised positions and their Uiso values were set to ride on the Ueq values of the parent carbon atoms. At the conclusion of the refinement, wR_2 = 0.094 and R_1 = 0.043 (2B) for all 4624 reflections weighted $w = [\sigma^2(F_o^2) + (0.0436\ P)^2 + 0.591\ P]^{-1}$ with $P = (F_o^2 + 2F_c^2)/3$; for the 'observed' data only, R_1 = 0.037.

In the final difference map, the highest peak (*ca* 0.17 $eÅ^{-3}$) was near the midpoint of the C(1)-C(9) bond.

Notes on the structure

The two isoindole groups, connected through the central N(41) atom, are essentially identical in dimensions except in the rotation of the phenyl group planes about the C(10)-C(11) and C(30)-C(31) bonds; the benzyl isoindole group of C(21) to N(38) is approximately planar whereas there is a rotation of *ca* 29.9 ° about the C(10)-C(11) bond in the group of C(1) to N(18).

All the bonds in the chain of N(2)-C(1)-N(41)-C(21)-N(22) are very similar, at *ca* 1.34 Å, suggesting delocalisation. However, the amino nitrogen atoms are different - the hydrogen on N(2) comes up strongly in early difference maps while the H on N(22) appears weaker. It is assumed that there is only one H atom here, shared unequally between the two N atoms. After refinement, the ratio of H(2):H(22) is *ca* 0.64:0.36. Both these hydrogen atoms are involved in hydrogen bonds - to the opposite N atom in the molecule.

Molecules are stacked in pairs about centres of symmetry: the ring of C(4)-C(9) lies over that of C(21'),N(22'),C(23'-24'),C(29'), with N(41') over the centre of the adjoining pyrazole ring of N(2), and C(1) and C(1') are almost directly superimposed, 3.292 Å apart. The phenyl rings of C(31-36) and C(31"-36") are overlapping with a C(36)-C(36") distance of 3.156 Å. Molecules are stacked parallel to the *b* axis through these π…π interactions.

Compound 4j

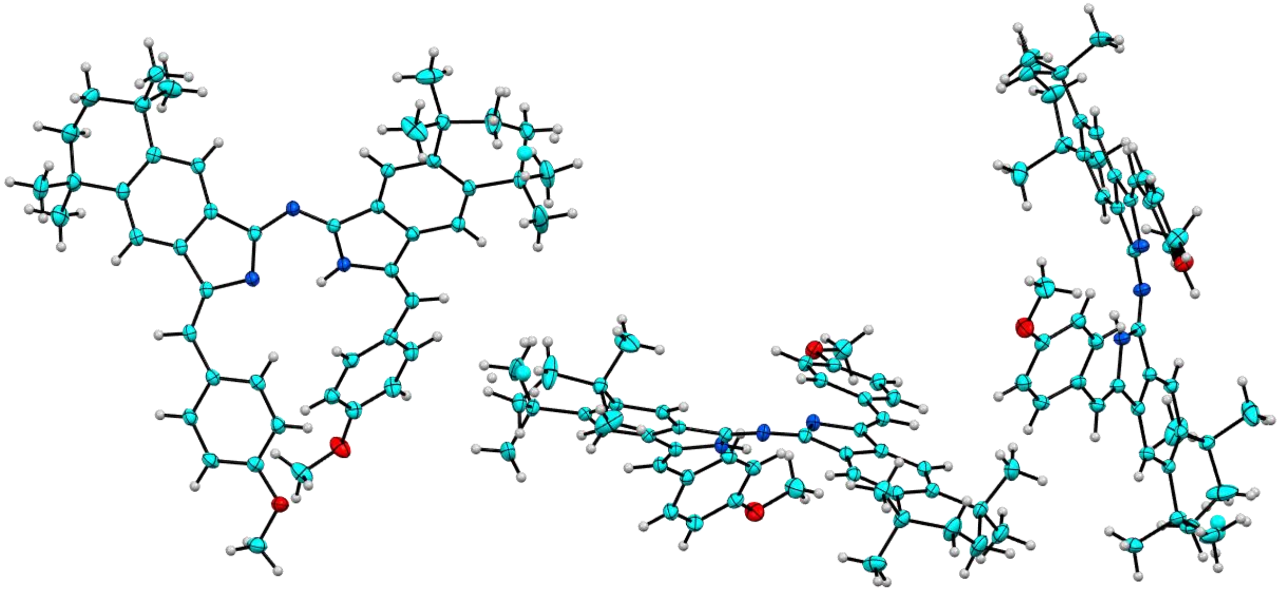

Crystal data and structure refinement for $(C_{24}H_{26}N_2O)$-N=$(C_{24}H_{27}N_2O)$

Identification code	faeza4
Elemental formula	C48 H53 N3 O2
Formula weight	703.93
Crystal system, space group	Monoclinic, P 2_1/c (no. 14)
Unit cell dimensions	a = 19.59378(19) Å α = 90.0 °<br>b = 16.33724(13) Å β = 96.1282(9) °<br>c = 12.15503(11) Å γ = 90.0 °
Volume	3868.69(6) Å^3
Z, Calculated density	4, 1.209 Mg/m^3
F(000)	1512
Absorption coefficient	0.566 mm^{-1}
Temperature	100.01(10) K
Wavelength	1.54184 Å
Crystal colour, shape	orange-red prism
Crystal size	0.20 x 0.17 x 0.09 mm
Crystal mounting:	on a small loop, in oil, fixed in cold N_2 stream
On the diffractometer:	
Theta range for data collection	3.531 to 74.989 °
Limiting indices	-20<=h<=24, -20<=k<=20, -15<=l<=15

Completeness to theta = 67.684 100.0 %

Absorption correction	Semi-empirical from equivalents
Max. and min. transmission	1.00000 and 0.72080
Reflections collected (not including absences)	115389
No. of unique reflections	7950 [R(int) for equivalents = 0.036]
No. of 'observed' reflections ($I > 2\sigma_I$)	7469
Structure determined by:	dual methods, in SHELXT
Refinement:	Full-matrix least-squares on F^2, in SHELXL
Data / restraints / parameters	7950 / 0 / 488
Goodness-of-fit on F^2	1.034
Final R indices ('observed' data)	$R_1 = 0.052$, $wR_2 = 0.127$
Final R indices (all data)	$R_1 = 0.054$, $wR_2 = 0.128$
Reflections weighted: $w = [\sigma^2(Fo^2)+(0.0552P)^2+3.017P]^{-1}$ where $P=(Fo^2+2Fc^2)/3$	
Extinction coefficient	n/a
Largest diff. peak and hole	0.86 and -0.68 e.Å^{-3}
Location of largest difference peak	near H(92a)

Crystal structure analysis of 4j

Crystal data: $C_{48}H_{53}N_3O_2$, M = 703.93. Monoclinic, space group $P2_1/c$ (no. 14), a = 19.59378(19), b = 16.33724(13), c = 12.15503(11) Å, β = 96.1282(9) °, V = 3868.69(6) Å^3. Z = 4, Dc = 1.209 g cm^{-3}, F(000) = 1512, T = 100.01(10) K, μ(Cu-Kα) = 5.66 cm^{-1}, λ(Cu-Kα) = 1.54184 Å.

The crystal was an orange-red prism, *ca* 0.20 x 0.17 x 0.09 mm, and was mounted, in oil, on a small loop and fixed in the cold nitrogen stream on a Rigaku Oxford Diffraction XtaLAB Synergy diffractometer, equipped with Cu-Kα radiation, HyPix detector and mirror monochromator. Intensity data were measured by thin-slice ω-scans. Total no. of reflections recorded, to θ_{max} = 75.0°, was 115389 of which 7950 were unique (Rint = 0.036); 7469 were 'observed' with $I > 2\sigma_I$.

Data were processed using the CrysAlisPro-CCD and -RED (Rigaku Oxford Diffraction Ltd., Abingdon, UK (2018)) programs. The structure was determined by the intrinsic phasing routines in the SHELXT program[33] and refined by full-matrix least-squares methods, on F^2's, in SHELXL.[34] The non-hydrogen atoms (except for C(91) in a disordered group) were refined with anisotropic thermal parameters. The hydrogen atom on N(22) was located in a difference map and was refined isotropically and freely. The remaining hydrogen atoms were included in idealised positions and their Uiso values were set to ride on

the Ueq values of the parent carbon atoms. At the conclusion of the refinement, $wR_2 = 0.128$ and $R_1 = 0.054$ (2B) for all 7950 reflections weighted $w = [\sigma^2(F_o^2) + (0.0552\ P)^2 + 3.017\ P]^{-1}$ with $P = (F_o^2 + 2F_c^2)/3$.

In the final difference map, the highest peak (*ca* 0.85 $e\text{Å}^{-3}$) was near H(92a) in the disordered region.

Notes on the structure

The hydrogen atom H(32) of the hydrogen bond was located clearly in a difference map and was refined freely, isotropically and satisfactorily. The bond distances and angles in the chain between N(2) and N(32) are in agreement with the arrangement of single- and double-bonds of the formula, with double-bond distances of 1.3235(18) and 1.3051(18) for C(1)-N(2) and N(1)-C(31) respectively.

In this crystal, there is no π-stacking of aromatic rings; most of the shorter intermolecular contacts are of the C-H…π type.

Compound 5b

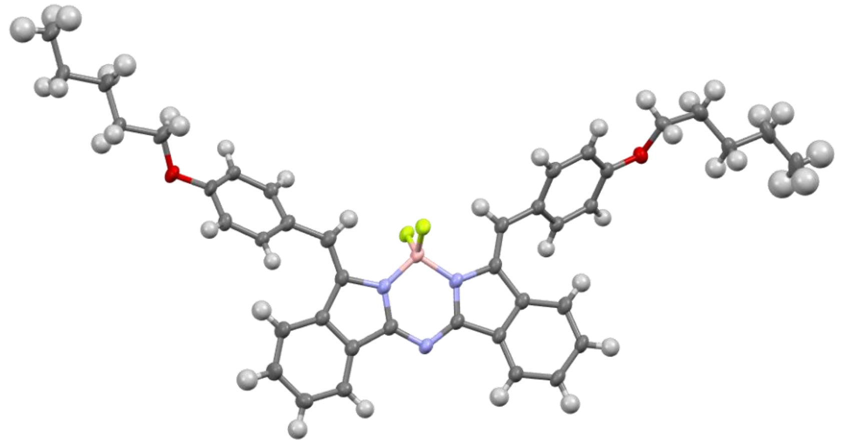

Crystal data and structure refinement for **5b**

Elemental formula	C40 H40 B F2 N3 O2
Formula weight	643.56
Crystal system, space group	Triclinic, P-1 (no. 2)
Unit cell dimensions	a = 9.5795(3) Å α = 75.693(3) °<br>b = 9.7567(3) Å β = 81.525(3) °<br>c = 18.4701(4) Å γ = 87.678(3) °
Volume	1654.47(8) Å^3
Z, Calculated density	2, 1.292 Mg/m^3
F(000)	680
Absorption coefficient	0.700 mm^{-1}
Temperature	120(2) K
Wavelength	1.54184 Å
Crystal colour, shape	red cuboid
Crystal size	0.03 x 0.09 x 0.35 mm
Crystal mounting:	on a small loop, in oil, fixed in cold N$_2$ stream
On the diffractometer:	
Theta range for data collection	7.643 to 69.998 °
Limiting indices	-11<=h<=11, -11<=k<=11, -22<=l<=18
Completeness to theta = 67.684	97.8 %
Absorption correction	Semi-empirical from equivalents

Max. and min. transmission 1.00000 and 0.87823

Reflections collected (not including absences) 18023

No. of unique reflections 6066 [R(int) for equivalents = 0.065]

No. of 'observed' reflections ($I > 2\sigma_I$) 4949

Structure determined by: dual methods, in SHELXT

Refinement: Full-matrix least-squares on F^2, in SHELXL

Data / restraints / parameters 6066 / 0 / 489

Goodness-of-fit on F^2 1.094

Final R indices ('observed' data) $R_1 = 0.054$, $wR_2 = 0.174$

Final R indices (all data) $R_1 = 0.072$, $wR_2 = 0.218$

Reflections weighted:
$w = [\sigma^2(Fo^2)+(0.1596P)^2+0.1184P]^{-1}$ where $P=(Fo^2+2Fc^2)/3$

Extinction coefficient n/a

Largest diff. peak and hole 0.43 and -0.36 e.A^{-3}

Location of largest difference peak near B(91)

___

Crystal structure analysis of 5b

Crystal data: $C_{40}H_{40}BF_2N_3O_2$, M = 643.56. Triclinic, space group P-1 (no. 2), a = 9.5795(3), b = 9.7567(3), c = 18.4701(4) Å, α = 75.693(3), β = 81.525(3), γ = 87.678(3) °, V = 1654.47(8) $Å^3$. Z = 2, Dc = 1.292 g cm^{-3}, F(000) = 680, T = 120.0(2) K, μ(Cu-Kα) = 7.0 cm^{-1}, λ(Cu-Kα) = 1.54184 Å.

The crystal was a red cuboid. From a sample under oil, one, *ca* 0.03 x 0.09 x 0.35 mm, was mounted on a small loop and fixed in the cold nitrogen stream on a Rigaku Oxford Diffraction XtaLAB Synergy diffractometer, equipped with Cu-Kα radiation, HyPix detector and mirror monochromator. Intensity data were measured by thin-slice ω-scans. Total no. of reflections recorded, to θ_{max} = 70.0°, was 18,023 of which 6066 were unique (Rint = 0.065); 4949 were 'observed' with I > $2\sigma_I$.

Data were processed using the CrysAlisPro-CCD and -RED (Rigaku Oxford Diffraction Ltd., Abingdon, UK (2018)) programs. The structure was determined by the intrinsic phasing routines in the SHELXT program[33] and refined by full-matrix least-squares methods, on F^2's, in SHELXL.[34] There is disorder in the orientation of the O-phenyl group of O(47) and in the ethyl group of C(51-52). The non-hydrogen atoms (including the partially occupied atoms) were refined with anisotropic thermal parameters. The hydrogen atoms were included in idealised positions and their Uiso values were set to ride on the Ueq values of the parent carbon atoms. At the conclusion of the refinement, wR_2 = 0.218 and R_1 = 0.072 (2B) for all 6066 reflections weighted $w = [\sigma^2(F_o^2) + (0.1596\ P)^2 + 0.1184\ P]^{-1}$ with $P = (F_o^2 + 2F_c^2)/3$; for the 'observed' data only, R_1 = 0.054.

In the final difference map, the highest peak (*ca* 0.4 $eÅ^{-3}$) was near B(91).

Notes on the structure

The molecule can be divided into three roughly planar units, (1) the phenyl group of C(10) to O(17), (2) from C(10) , through the two isoindole groups to C(41), and (3) the phenyl group of C41) to O(47); the latter phenyl ring is disordered in two orientations. The *n*-pentyl chain on O(17) has an all-*trans* conformation; the second *n*-pentyl chain has an approximately all-*trans* orientation with minor deviations resulting from disorder at both the O(47)/O(47a) end and in the end ethyl group.

Despite the presence of several planar aromatic groups in the molecule, there are no interplanar π…π interactions in these crystals. The closest interactions appear to be between C(45) and the related atom across the inversion centre at (½, ½, 1); these atoms are, however, disordered with site occupancies of 0.5,

and the closest atoms to C(45) will be with atoms of the alternative conformation at normal van der Waals' distances, e.g. C(45)…C(45a') at 3.850 Å.

Compound 5d

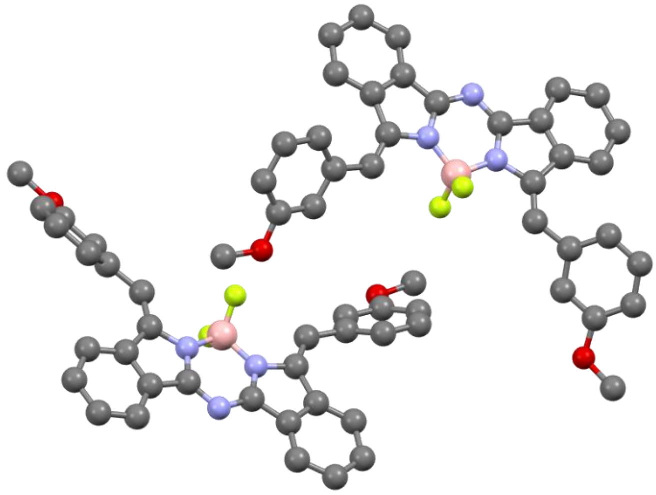

Crystal data and structure refinement for 5d

Identification code	5d
Elemental formula	2(C32 H24 B1 F2 N3 O2)
Formula weight	1062.70
Crystal system, space group	Orthorhombic, P 2_1 2_1 2 (no. 18)
Unit cell dimensions	a = 22.0145(8) Å α = 90 °<br>b = 27.0662(9) Å β = 90 °<br>c = 8.6554(4) Å γ = 90 °
Volume	5157.3(3) $Å^3$
Z, Calculated density	4, 1.369 Mg/m^3
F(000)	2208
Absorption coefficient	0.784 mm^{-1}
Temperature	100.01(10) K
Wavelength	1.54184 Å
Crystal colour, shape	yellow block
Crystal size	0.18 x 0.05 x 0.02 mm
Crystal mounting:	on a small loop, in oil, fixed in cold N_2 stream
On the diffractometer:	
Theta range for data collection	7.686 to 52.066 °
Limiting indices	-22<=h<=22, -27<=k<=27, -8<=l<=8
Completeness to theta = 52.066	98.8 %
Absorption correction	Semi-empirical from equivalents

Max. and min. transmission	1.00000 and 0.62028
Reflections collected (not including absences)	54513
No. of unique reflections	5715 [R(int) for equivalents = 0.109]
No. of 'observed' reflections ($I > 2\sigma_I$)	5033
Structure determined by:	dual methods, in SHELXT
Refinement:	Full-matrix least-squares on F^2, in SHELXL
Data / restraints / parameters	5715 / 0 / 721
Goodness-of-fit on F^2	0.990
Final R indices ('observed' data)	R_1 = 0.039, wR_2 = 0.092
Final R indices (all data)	R_1 = 0.046, wR_2 = 0.094
Reflections weighted: $w = [\sigma^2(Fo^2)+(0.0669P)^2]$ where $P=(Fo^2+2Fc^2)/3$	
Absolute structure parameter	0.01(9)
Extinction coefficient	n/a
Largest diff. peak and hole	0.20 and -0.20 e.A-3
Location of largest difference peak	near C(62)

Crystal structure analysis of 5d

Crystal data: 2($C_{32}H_{24}B_1F_2N_3O_2$), M = 1062.70. Orthorhombic, space group P 2_1 2_1 2 (no. 18), a = 22.0145(8), b = 27.0662(9), c = 8.6554(4) Å, V = 5157.3(3) $Å^3$. Z = 4, Dc = 1.369 g cm^{-3}, F(000) = 2208, T = 100(2) K, μ(Cu-Kα) = 7.84 cm^{-1}, λ(Cu-Kα) = 1.54184 Å.

The crystal was a yellow block. From a sample under oil, one, *ca* 0.18 x 0.05 x 0.02 mm, was mounted on a small loop and fixed in the cold nitrogen stream on a Rigaku Oxford Diffraction XtaLAB Synergy diffractometer, equipped with Cu-Kα radiation, HyPix detector and mirror monochromator. Intensity data were measured by thin-slice ω-scans. Total no. of reflections recorded, to θ_{max} = 52.1°, was 54,513 of which 5,715 were unique (Rint = 0.109); 5,033 were 'observed' with I > $2\sigma_I$.

Data were processed using the CrysAlisPro-CCD and -RED (Rigaku Oxford Diffraction Ltd., Abingdon, UK (2018)) programs. The structure was determined by the intrinsic phasing routines in the SHELXT program[33] and refined by full-matrix least-squares methods, on F^2's, in SHELXL.[34] There are two almost identical molecules in the unit cell. The non-hydrogen atoms were refined with anisotropic thermal parameters. The hydrogen atoms were included in idealised positions and their Uiso values were set to ride on the Ueq values of the parent carbon atoms. At the conclusion of the refinement, wR_2 = 0.094 and R_1 = 0.046 (2B) for all 5715 reflections weighted $w = [\sigma^2(F_o^2) + (0.0669\ P)^2]^{-1}$ with $P = (F_o^2 + 2F_c^2)/3$; for the 'observed' data only, R_1 = 0.039.

In the final difference map, the highest peak (*ca* 0.2 e$Å^{-3}$) was near C(62).

Notes on the structure

There are two independent molecules in this crystal; they are related by a pseudo-twofold symmetry axis. Each molecule also shows pseudo-symmetry about the plane containing the boron atom, the two fluorine atoms and the N(2) or N(22) atom; the symmetry does not extend beyond this particular molecule.

These molecules are very similar in conformation; the central C_2N_3B ring in each molecule has a shallow envelope shape with B(1) and B(41) displaced 0.065(5) and 0.047(5) Å from the good mean-planes of the other five ring atoms. The normals to these two planes, related by the pseudo-symmetry, are 10.8(2)° apart. In each molecule, the adjoining isoindoline groups are essentially coplanar with the central C_2N_3B rings. In every case (in these two adjoining molecules), there is rotation about the C(10)–C(11) type bond by *ca* 45°; this brings the four O atoms out of the general molecular plane, and they all lie on the same side of that plane and are displaced *ca* 1.3 Å from the plane.

The central C_2N_3B rings are essentially planar and show aromatic mean dimensions, viz. B–N 1.539, as N(1)–C(2) 1.348, as N(2)–C(22) 1.334 Å, and in the isoindoline-phenyl ring link, as C(9)–C(10) and C(10)–C(11) 1.348 and 1.461 Å, respectively.

The molecules are stacked by overlap of the $BN(isoindoline)_2$ ring systems in off-set columns parallel to the *c* axis.

The columns are linked to symmetry-related columns through 'weak hydrogen bonding'.

Compound 5f

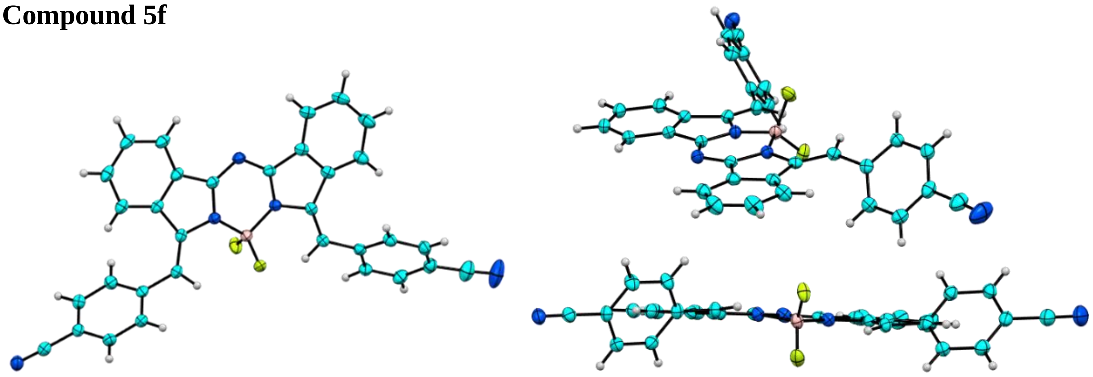

Crystal data and structure refinement for **5f**

Identification code	burdur10a - CNB-4
Elemental formula	C32 H18 B F2 N5, 0.5(C H2 Cl2)
Formula weight	563.79
Crystal system, space group	Orthorhombic, Pbca (no, 61)
Unit cell dimensions	a = 16.2443(2) Å α = 90 °<br>b = 15.9943(2) Å β = 90 °<br>c = 20.3605(2) Å γ = 90 °
Volume	5289.99(11) $Å^3$
Z, Calculated density	8, 1.416 Mg/m^3
F(000)	2312
Absorption coefficient	1.783 mm^{-1}
Temperature	100.01(10) K
Wavelength	1.54184 Å
Crystal colour, shape	dark yellow block
Crystal size	0.45 x 0.35 x 0.3 mm
Crystal mounting:	on a small loop, in oil, fixed in cold N_2 stream
On the diffractometer:	
Theta range for data collection	7.775 to 72.496 °
Limiting indices	-16<=h<=20, -16<=k<=19, -25<=l<=23
Completeness to theta = 67.684	99.5 %
Absorption correction	Semi-empirical from equivalents

Max. and min. transmission 1.00000 and 0.59544

Reflections collected (not including absences) 21805

No. of unique reflections 5148 [R(int) for equivalents = 0.033]

No. of 'observed' reflections ($I > 2\sigma_I$) 4449

Structure determined by: dual methods, in SHELXT

Refinement: Full-matrix least-squares on F^2, in SHELXL

Data / restraints / parameters 5148 / 0 / 388

Goodness-of-fit on F^2 1.057

Final R indices ('observed' data) $R_1 = 0.043$, $wR_2 = 0.110$

Final R indices (all data) $R_1 = 0.050$, $wR_2 = 0.113$

Reflections weighted:
$w = [\sigma^2(Fo^2)+(0.0521P)^2+3.0672P]^{-1}$ where $P=(Fo^2+2Fc^2)/3$

Extinction coefficient 0.00022(7)

Largest diff. peak and hole 0.54 and -0.44 e.Å^{-3}

Location of largest difference peak near N(28)

Crystal structure analysis of 5f

Crystal data: $C_{32}H_{18}BF_2N_5$, 0.5(CH_2Cl_2), M = 563.79. Orthorhombic, space group Pbca (no. 61), a = 16.2443(2), b = 15.9943(2), c = 20.3605(2) Å, V = 5289.99(11) Å^3. Z = 8, Dc = 1.416 g cm^{-3}, F(000) = 2312, T = 100.01(10) K, μ(Cu-Kα) = 17.83 cm^{-1}, λ(Cu-Kα) = 1.54184 Å.

The crystals were dark yellow blocks. From a sample under oil, one, *ca* 0.3 x 0.35 x 0.45 mm, was mounted on a small loop and fixed in the cold nitrogen stream on a Rigaku Oxford Diffraction XtaLAB Synergy diffractometer, equipped with Cu-Kα radiation, HyPix detector and mirror monochromator. Intensity data were measured by thin-slice ω-scans. Total no. of reflections recorded, to θ_{max} = 72.5°, was 21805 of which 5148 were unique (Rint = 0.033); 4449 were 'observed' with I > $2\sigma_I$.

Data were processed using the CrysAlisPro-CCD and -RED (Rigaku Oxford Diffraction Ltd., Abingdon, UK (2018)) programs. The structure was determined by the intrinsic phasing routines in the SHELXT program[33] and refined by full-matrix least-squares methods, on F^2's, in SHELXL.[34] The principal molecule was clear and well-defined. A solvent (CH_2Cl_2) molecule was found, disordered about a centre of symmetry. All the non-hydrogen atoms were refined with anisotropic thermal parameters. The hydrogen atoms were included in idealised positions and their Uiso values were set to ride on the Ueq values of the parent carbon atoms. At the conclusion of the refinement, wR_2 = 0.113 and R_1 = 0.050 (2B) for all 5148 reflections weighted $w = [\sigma^2(F_o^2) + (0.0521\ P)^2 + 3.067\ P]^{-1}$ with $P = (F_o^2 + 2F_c^2)/3$; for the 'observed' data only, R_1 = 0.043.

In the final difference map, the highest peak (*ca* 0.54 eÅ^{-3}) was near N(28).

Notes on the structure

The BF_2-linked N(bis-isoindoline) derivative molecule has a three-plane structure. The central plane comprises the two isoindole groups and the N(10) and B atoms that link them; the isoindole rings are tilted only slightly from the mean-plane of the central six-membered ring. The two phenyl rings are rotated from the isoindole rings, about the C(20)-C(21) and C(30)-C(31) bonds, by 52.2 and 61.8 ° respectively.

The solvent molecule, CH_2Cl_2, is disordered and lies close to a centre of symmetry with Cl(53’) 0.66 A from the C(51)-Cl(53) bond.

There are several short intramolecular contacts, e.g. H(15)-C(22) and, correspondingly, H(5)-H(36), both at 2.57 Å. Most of the intermolecular contacts are close to van der Waals’ distances.

Compound 8

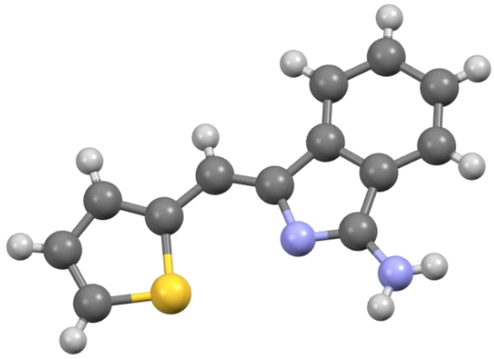

Crystal data and structure refinement for **8**

Identification code	norahalb
Elemental formula	C13 H10 N2 S
Formula weight	226.29
Crystal system, space group	Triclinic, P-1 (no. 2)
Unit cell dimensions	a = 12.0847(2) Å α = 95.9946(13) °<br>b = 13.8133(2) Å β = 102.3784(14) °<br>c = 13.9405(2) Å γ = 95.4710(13) °
Volume	2243.89(6) A3
Z, Calculated density	8, 1.340 Mg/m3
F(000)	944
Absorption coefficient	2.314 mm-1
Temperature	100.01(10) K
Wavelength	1.54184 Å
Crystal colour, shape	yellow plate
Crystal size	0.333 x 0.037 x 0.013 mm
Crystal mounting:	in a small loop, in oil, fixed in cold N_2 stream
On the diffractometer:	
Theta range for data collection	3.241 to 69.999 °
Limiting indices	-14<=h<=13, -15<=k<=16, -16<=l<=16
Completeness to theta = 67.684	98.5 %
Absorption correction	Semi-empirical from equivalents
Max. and min. transmission	1.00000 and 0.82519
Reflections collected (not including absences)	23359

No. of unique reflections	8299 [R(int) for equivalents = 0.031]
No. of 'observed' reflections ($I > 2\sigma_I$)	7729
Structure determined by:	dual methods, in SHELXT
Refinement:	Full-matrix least-squares on F^2, in SHELXL
Data / restraints / parameters	8299 / 0 / 617
Goodness-of-fit on F^2	1.065
Final R indices ('observed' data)	R_1 = 0.043, wR_2 = 0.126
Final R indices (all data)	R1 = 0.045, wR2 = 0.128
Reflections weighted: $w = [\sigma^2(Fo^2)+(0.0761P)^2+1.5555P]^{-1}$ where $P=(Fo^2+2Fc^2)/3$	
Extinction coefficient	n/a
Largest diff. peak and hole	0.73 and -0.62 e.A-3
Location of largest difference peak	near C(25)

Crystal structure analysis of 8

Crystal data: $C_{13}H_{10}N_2S$, M = 226.29. Triclinic, space group P-1 (no. 2), a = 12.0847(2), b = 13.8133(2), c = 13.9405(2) Å, α = 95.9946(13), β = 102.3784(14), γ = 95.4710(13) °, V = 2243.89(6) $Å^3$. Z = 8, Dc = 1.340 g cm^{-3}, F(000) = 944, T = 100.01(10) K, μ(Cu-Kα) = 23.14 cm^{-1}, λ(Cu-Kα) = 1.54184 Å.

The crystal was a yellow plate. From a sample under oil, one, *ca* 0.013 x 0.037 x 0.333 mm, was mounted on a small loop and fixed in the cold nitrogen stream on a Rigaku Oxford Diffraction XtaLAB Synergy diffractometer, equipped with Cu-Kα radiation, HyPix detector and mirror monochromator. Intensity data were measured by thin-slice ω-scans. Total no. of reflections recorded, to θ_{max} = 70.0°, was 23359 of which 8299 were unique (Rint = 0.031); 7729 were 'observed' with $I > 2\sigma_I$.

Data were processed using the CrysAlisPro-CCD and -RED (Rigaku Oxford Diffraction Ltd., Abingdon, UK (2018)) programs. The structure was determined by the intrinsic phasing routines in the SHELXT program[33] and refined by full-matrix least-squares methods, on F^2's, in SHELXL.[34]

There are four molecules in the asymmetric unit. The non-hydrogen atoms were refined with anisotropic thermal parameters. The hydrogen atoms were included in idealised positions and their Uiso values were refined freely. At the conclusion of the refinement, wR_2 = 0.128 and R_1 = 0.045 (2B) for all 8299 reflections weighted $w = [\sigma^2(F_o^2) + (0.00761\ P)^2 + 1.556\ P]^{-1}$ with $P = (F_o^2 + 2F_c^2)/3$; for the 'observed' data only, R_1 = 0.043.

In the final difference map, the highest peak (*ca* 0.7 $eÅ^{-3}$) was near C(25).

Notes on the structure

There are four independent molecules of the amino-isoindoline in this crystal and these are linked in pairs to form hydrogen bonded dimers. In each molecule, bond dimensions confirm (generally) that there are double bonds at C(3)-C(4), C(1)-C(5) and C(14)-N(15).

All four molecules are close to planar, with the angle between the normals to the two ring groups at 12.67(6), 13.57(8), 7.66(6) and 5.15(7) °.

Compound 21

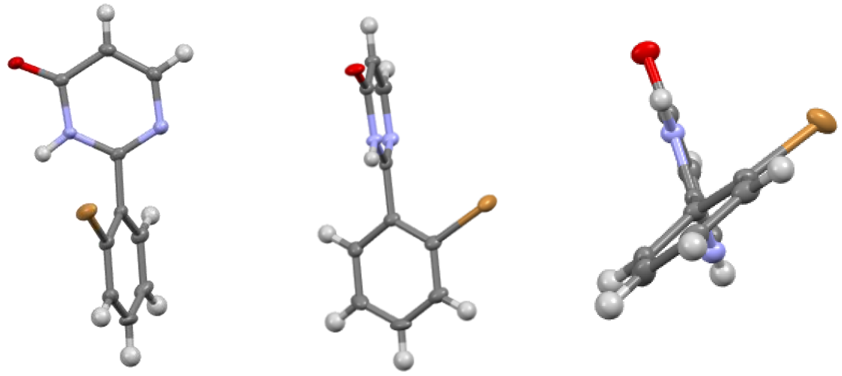

Crystal data and structure refinement for **21**

Elemental formula	C10 H7 Br N2 O
Formula weight	251.09
Crystal system, space group	Monoclinic, $P2_1/n$ (no. 14)
Unit cell dimensions	a = 7.24571(9) Å α = 90.0 °
	b = 15.9923(2) Å β = 99.5026(13) °
	c = 8.43128(12) Å γ = 90.0 °
Volume	963.57(2) $Å^3$
Z, Calculated density	4, 1.731 Mg/m^3
F(000)	496
Absorption coefficient	5.543 mm^{-1}
Temperature	100(2) K
Wavelength	1.54184 Å
Crystal colour, shape	yellow block
Crystal size	0.106 x 0.202 x 0.239 mm
Crystal mounting:	on a small loop, in oil, fixed in cold N_2 stream
On the diffractometer:	
Theta range for data collection	7.687 to 72.303 °
Limiting indices	-8<=h<=8, -18<=k<=19, -10<=l<=9
Completeness to theta = 67.684	99.5 %
Absorption correction	Semi-empirical from equivalents
Max. and min. transmission	1.00000 and 0.74244
Reflections collected (not including absences)	6131

No. of unique reflections 1854 [R(int) for equivalents = 0.031]

No. of 'observed' reflections ($I > 2\sigma_I$) 1760

Structure determined by: dual methods, in SHELXT

Refinement: Full-matrix least-squares on F^2, in SHELXL

Data / restraints / parameters 1854 / 0 / 127

Goodness-of-fit on F^2 1.049

Final R indices ('observed' data) $R_1 = 0.032$, $wR_2 = 0.082$

Final R indices (all data) $R_1 = 0.033$, $wR_2 = 0.083$

Reflections weighted:
$w = [\sigma^2(Fo^2)+(0.0428P)^2+1.3106P]^{-1}$ where $P=(Fo^2+2Fc^2)/3$

Extinction coefficient n/a

Largest diff. peak and hole 0.52 and -0.58 e.Å^{-3}

Location of largest difference peak near Br(12)

Crystal structure analysis of 21

Crystal data: $C_{10}H_7BrN_2O$, M = 251.09. Monoclinic, space group $P2_1/n$ (as no. 14), a = 7.2457(9), b = 15.9923(2), c = 8.43128(12) Å, β = 99.5026(13) °, V = 963.57(2) $Å^3$. Z = 4, Dc = 1.731 g cm^{-3}, F(000) = 496, T = 100(2) K, μ(Cu-Kα) = 5.543 cm^{-1}, λ(Cu-Kα) = 1.54184 Å.

The crystal was a yellow block. From a sample under oil, one, *ca* 0.106 x 0.202 x 0.239 mm, was mounted on a small loop and fixed in the cold nitrogen stream on a Rigaku Oxford Diffraction XtaLAB Synergy diffractometer, equipped with Cu-Kα radiation, HyPix detector and mirror monochromator. Intensity data were measured by thin-slice ω-scans. Total no. of reflections recorded, to θ_{max} = 72.3°, was 6131 of which 1854 were unique (Rint = 0.031); 1760 were 'observed' with $I > 2\sigma_I$.

Data were processed using the CrysAlisPro-CCD and -RED (Rigaku Oxford Diffraction Ltd., Abingdon, UK (2018)) programs. The structure was determined by the intrinsic phasing routines in the SHELXT program[33] and refined by full-matrix least-squares methods, on F^2's, in SHELXL.[34] The non-hydrogen atoms were refined with anisotropic thermal parameters. The hydrogen atoms were included in idealised positions and their Uiso values were set to ride on the Ueq values of the parent carbon or nitrogen atoms. At the conclusion of the refinement, wR_2 = 0.083 and R_1 = 0.033 (2B) for all 1854 reflections weighted w = $[\sigma^2(F_o^2) + (0.0428\ P)^2 + 1.311\ P]^{-1}$ with P = $(F_o^2 + 2F_c^2)/3$; for the 'observed' data only, R_1 = 0.032.

In the final difference map, the highest peak (*ca* 0.5 e$Å^{-3}$) was near Br(12).

Notes on the structure

The two six-membered rings are each essentially planar and there is rotation about the C(1)–C(11) bond so that their normals are 69.51(7) ° apart. In addition to the short hydrogen bond N(2)–H(2)…O(3) listed, there is also a short intermolecular bond involving the bromine atom, Br(12)…N(6') at 3.225 Å with the C(12)–Br(12)…N(6') angle at 173.39 °.

Compound 22

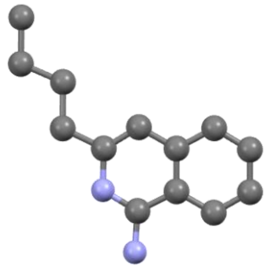

Crystal data and structure refinement for 3-*n*-butyl-isoquinolin-1-amine

Identification code	22
Elemental formula	C13 H16 N2
Formula weight	200.28
Crystal system, space group	Monoclinic, P 2_1/c (no. 14)
Unit cell dimensions	a = 10.5288(4) Å α = 90 °
	b = 5.4244(2) Å β = 103.423(4) °
	c = 19.3720(7) Å γ = 90 °
Volume	1076.16(7) Å^3
Z, Calculated density	4, 1.236 Mg/m^3
F(000)	432
Absorption coefficient	0.074 mm^{-1}
Temperature	140(1) K
Wavelength	0.71073 Å
Crystal colour, shape	yellow prism
Crystal size	0.35 x 0.18 x 0.07 mm
Crystal mounting:	on a glass fibre, in oil, fixed in cold N_2 stream
On the diffractometer:	
Theta range for data collection	3.260 to 27.496 °
Limiting indices	-13<=h<=13, -7<=k<=7, -25<=l<=25
Completeness to theta = 25.242	99.8 %
Absorption correction	Semi-empirical from equivalents
Max. and min. transmission	1.000 and 0.742
Reflections collected (not including absences)	15704
No. of unique reflections	2466 [R(int) for equivalents = 0.037]
No. of 'observed' reflections ($I > 2\sigma_I$)	1958

Structure determined by:	dual methods, in SHELXT
Refinement:	Full-matrix least-squares on F^2, in SHELXL
Data / restraints / parameters	2466 / 0 / 200
Goodness-of-fit on F^2	1.054
Final R indices ('observed' data)	R_1 = 0.043, wR_2 = 0.099
Final R indices (all data)	R_1 = 0.058, wR_2 = 0.104
Reflections weighted: $w = [\sigma^2(Fo^2)+(0.0464P)^2+0.2192P]^{-1}$ where $P=(Fo^2+2Fc^2)/3$	
Extinction coefficient	n/a
Largest diff. peak and hole	0.23 and -0.22 e.Å^{-3}
Location of largest difference peak	at mid-point of C(1)-C(9) bond

Crystal structure analysis of 3-*n*-butyl-isoquinolin-1-amine 22

Crystal data: $C_{13}H_{16}N_2$, M = 200.28. Monoclinic, space group $P2_1/c$ (no. 14), a = 10.5288(4), b = 5.4244(2), c = 19.3720(7) Å, β = 103.423(4) °, V = 1076.16(7) $Å^3$. Z = 4, Dc = 1.236 g cm^{-3}, F(000) = 432, T = 140(1) K, μ(Mo-Kα) = 0.74 cm^{-1}, λ(Mo-Kα) = 0.71073 Å.

Crystals are yellow prisms. From a sample under oil, one, *ca* 0.07 x 0.18 x 0.35 mm, was mounted on a glass fibre and fixed in the cold nitrogen stream on an Oxford Diffraction Xcalibur-3/Sapphire3-CCD diffractometer, equipped with Mo-Kα radiation and graphite monochromator. Intensity data were measured by thin-slice ω- and φ-scans. Total no. of reflections recorded, to θ_{max} = 27.5°, was 15704 of which 2466 were unique (Rint = 0.037); 1958 were 'observed' with $I > 2\sigma_I$.

Data were processed using the CrysAlisPro-CCD and -RED (Rigaku Oxford Diffraction Ltd., Abingdon, UK (2018)) programs. The structure was determined by the intrinsic phasing routines in the SHELXT program[33] and refined by full-matrix least-squares methods, on F^2's, in SHELXL.[34] The non-hydrogen atoms were refined with anisotropic thermal parameters. Hydrogen atoms were located in a difference map and were refined freely. At the conclusion of the refinement, wR_2 = 0.104 and R_1 = 0.058 (2B) for all 2466 reflections weighted $w = [\sigma^2(F_o^2) + (0.0464P)^2 + 0.2192P]^{-1}$ with $P = (F_o^2 + 2F_c^2)/3$; for the 'observed' data only, R_1 = 0.043.

In the final difference map, the highest peaks (*ca* 0.23 $eÅ^{-3}$) were close to the mid-points of the ring C-C bonds.

Notes on the structure

All the non-hydrogen atoms of the isoquinoline rings of the title molecule form a good planar group; the carbon atoms of the *n*-butyl group lie close to this plane and show an all-*trans* chain. One of the amino H atoms forms a good hydrogen bond to the pyridine N atom of a neighbouring molecule, and this bonding is repeated about a centre of symmetry, thus forming an eight-membered ring which links the pair of molecules in a dimer unit.

Compound 24

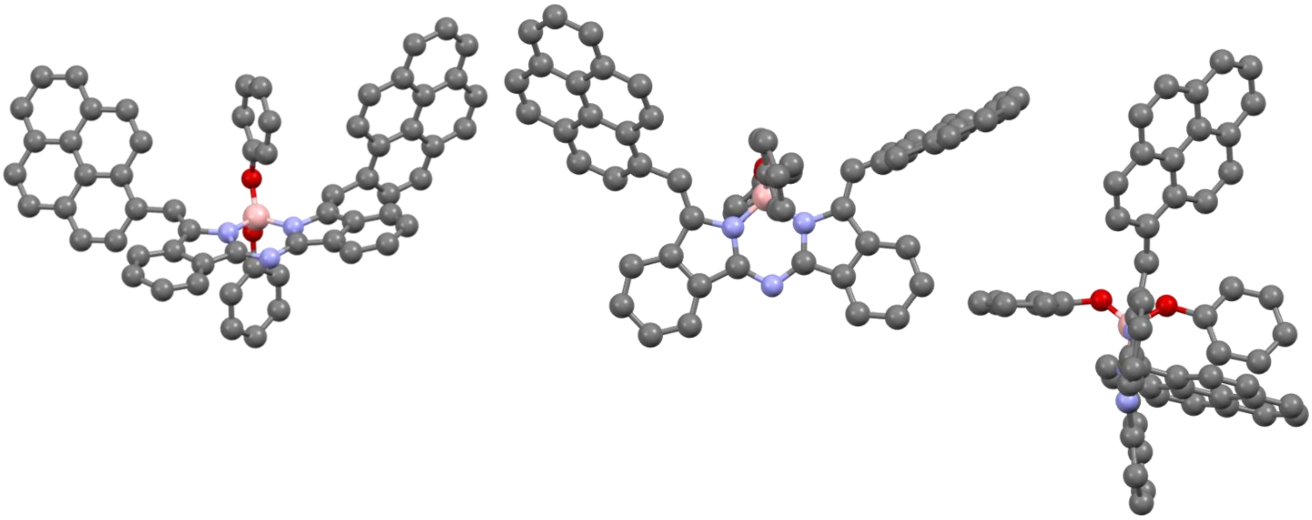

Crystal data and structure refinement details.

Identification code	**24dlsya**	
Empirical formula	$C_{62}H_{38}BN_3O_2$	
Formula weight	867.76	
Temperature	30(2) K	
Wavelength	0.6889 Å	
Crystal system	Monoclinic	
Space group	*P*121/*c*1	
Unit cell dimensions	*a* = **13.8638(12) Å**	α = **90°**
	b = **20.4510(7) Å**	β = **136.111(15)°**
	c = **21.9396(16) Å**	γ = **90°**
Volume	4312.4(9) Å^3	
Z	4	
Density (calculated)	1.337 Mg / m^3	
Absorption coefficient	0.076 mm^{-1}	
F(000)	1808	
Crystal	Plate; red	
Crystal size	0.15 × 0.12 × 0.03 mm^3	
θ range for data collection	1.730 – 26.580°	
Index ranges	$-13 \le h \le 17$, $-26 \le k \le 26$, $-28 \le l \le 26$	

Reflections collected	40586
Independent reflections	9637 [R_{int} = 0.0550]
Completeness to θ = 24.415°	98.3 %
Absorption correction	Semi–empirical from equivalents
Max. and min. transmission	1.00000 and 0.38935
Refinement method	Full-matrix least-squares on F^2
Data / restraints / parameters	9637 / 0 / 613
Goodness-of-fit on F^2	1.043
Final R indices [$F^2 > 2\sigma(F^2)$]	$R1$ = 0.0577, $wR2$ = 0.1589
R indices (all data)	$R1$ = 0.0664, $wR2$ = 0.1681
Extinction coefficient	n/a
Largest diff. peak and hole	0.456 and –0.242 e Å^{-3}

Diffractometer: Beamline I19 situated on an undulator insertion device with a combination of double crystal monochromator, vertical and horizontal focussing mirrors and a series of beam slits (primary white beam and either side of the focussing mirrors). The experimental hutch (EH1) is equipped with a Crystal Logic 4-circle kappa geometry goniometer with a Rigaku Saturn 724 CCD detector and an Oxford Cryosystems Cryostream plus cryostat (80-500K). For conventional service crystallography the beamline operates at a typical energy of 18 keV (Zr K absorption edge) and a Rigaku ACTOR robotic sample changing system is available. **Cell determination and data collection**: *CrystalClear-SM Expert 2.0 r5* (Rigaku, 2010). **Data reduction, cell refinement and absorption correction**: *CrysAlisPro 1.171.37.35* (Agilent, 2014). **Structure solution**: *SHELXT-2014.*[33] **Structure refinement**: *SHELXL-2014.*[34b]

Compound 25

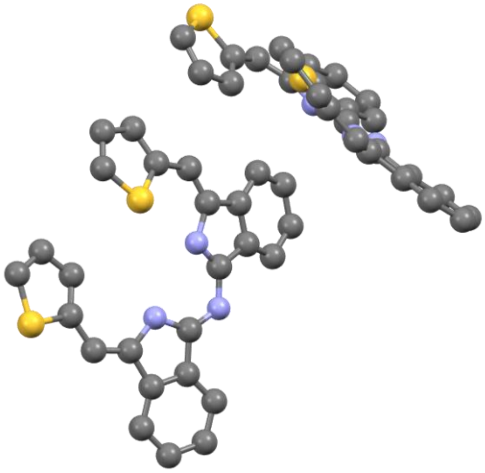

Crystal data and structure refinement for 25

Identification code	25
Elemental formula	C26 H17 N3 S2
Formula weight	435.54
Crystal system, space group	Monoclinic, P 2_1/c (no. 14)
Unit cell dimensions	a = 6.2874(4) Å α = 90 °<br>b = 14.8194(4) Å β = 92.138(4) °<br>c = 43.7886(9) Å γ = 90 °
Volume	4077.2(3) $Å^3$
Z, Calculated density	8, 1.419 Mg/m^3
F(000)	1808
Absorption coefficient	2.513 mm-1
Temperature	99.98(13) K
Wavelength	1.54184 Å
Crystal colour, shape	dark orange rod
Crystal size	0.070 x 0.018 x 0.016 mm
Crystal mounting:	in a small loop, in oil, fixed in cold N_2 stream
On the diffractometer:	
Theta range for data collection	7.659 to 69.993 °
Limiting indices	-7<=h<=7, -9<=k<=17, -53<=l<=52
Completeness to theta = 67.684	99.5 %
Absorption correction	Semi-empirical from equivalents

Max. and min. transmission	1.00000 and 0.76014
Reflections collected (not including absences)	27022
No. of unique reflections	7680 [R(int) for equivalents = 0.086]
No. of 'observed' reflections ($I > 2\sigma_I$)	6474
Structure determined by:	dual methods, in SHELXT
Refinement:	Full-matrix least-squares on F^2, in SHELXL
Data / restraints / parameters	7680 / 0 / 561
Goodness-of-fit on F^2	1.089
Final R indices ('observed' data)	$R_1 = 0.066$, $wR_2 = 0.207$
Final R indices (all data)	$R_1 = 0.085$, $wR_2 = 0.235$
Reflections weighted: $w = [\sigma^2(Fo^2)+(0.1252P)^2+12.0041P]^{-1}$ where $P=(Fo^2+2Fc^2)/3$	
Extinction coefficient	n/a
Largest diff. peak and hole	0.79 and -0.60 e.Å^{-3}
Location of largest difference peak	close to C(75)

Crystal structure analysis of 25

Crystal data: C_{26} H_{17} N_3 S_2, M = 435.54. Monoclinic, space group $P2_1/c$ (no. 14), a = 6.2874(4), b = 14.8194(4), c = 43.7886(9) Å, β = 92.138(4) °, V = 4077.2(3) $Å^3$. Z = 8, Dc = 1.419 g cm^{-3}, F(000) = 1808, T = 99.98(13) K, μ(Cu-Kα) = 25.1 cm^{-1}, λ(Cu-Kα) = 1.54184 Å.

The crystal was a dark orange rod. From a sample under oil, one, *ca* 0.016 x 0.018 x 0.070 mm, was mounted on a small loop and fixed in the cold nitrogen stream on a Rigaku Oxford Diffraction XtaLAB Synergy diffractometer, equipped with Cu-Kα radiation, HyPix detector and mirror monochromator. Intensity data were measured by thin-slice ω-scans. Total no. of reflections recorded, to θ_{max} = 70.0°, was 27022 of which 7680 were unique (Rint = 0.086); 6474 were 'observed' with $I > 2\sigma_I$.

Data were processed using the CrysAlisPro-CCD and -RED (Rigaku Oxford Diffraction Ltd., Abingdon, UK (2018)) programs. The structure was determined by the intrinsic phasing routines in the SHELXT program[33] and refined by full-matrix least-squares methods, on F^2's, in SHELXL.[34] There are two very similar molecules in this crystal. The non-hydrogen atoms were refined with anisotropic thermal parameters. The C-H hydrogen atoms were included in idealised positions and their Uiso values were set to ride on the Ueq values of the parent carbon atoms. Hydrogen atoms were also placed on all the nitrogen atoms; on refinement of their Uiso values, only one on each molecule gave an acceptable result, indicating the likely N-H groups. At the conclusion of the refinement, wR_2 = 0.235 and R_1 = 0.085 (2B) for all 7680 reflections weighted $w = [\sigma^2(F_o^2) + (0.1252\ P)^2 + 12.00\ P]^{-1}$ with $P = (F_o^2 + 2F_c^2)/3$; for the 'observed' data only, R_1 = 0.066.

In the final difference map, the highest peak (*ca* 0.8 e$Å^{-3}$) was close to C(75).

Notes on the structure

From a second crystal, the diffraction data were stronger, and produced an improved refinement with lower R-factors and more precise molecular dimensions. The preliminary coordinates, from the first crystal, were input into the SHELXL system and the refinement process was continued smoothly to convergence. The assignment of the hydrogen atoms on N(21) and N(61) was confirmed; these are the donor atoms in intramolecular hydrogen bonds to N(1) and N(41) respectively. There is no extensive π…π stacking in the crystal despite the variety of planar aromatic units.

Compound 33

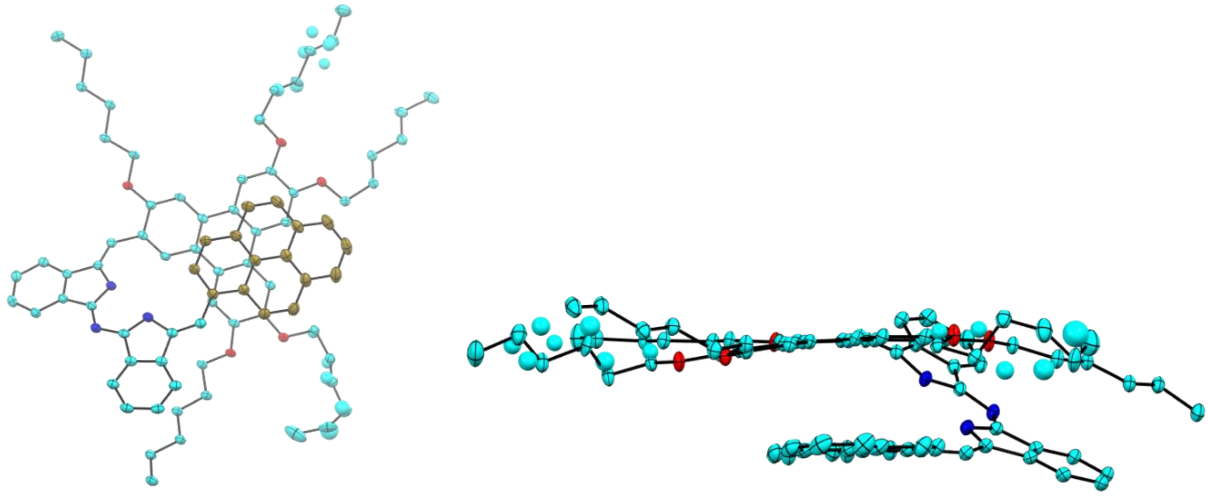

Crystal data and structure refinement for **33**

Identification code	33
Elemental formula	C82 H91 N3 O5
Formula weight	1198.57
Crystal system, space group	Triclinic, P-1 (no. 2)
Unit cell dimensions	a = 11.5732(2) Å α = 117.737(2) °<br>b = 17.8975(3) Å β = 97.567(2) °<br>c = 18.0680(3) Å γ = 91.4000(10) °
Volume	3267.86(11) $Å^3$
Z, Calculated density	2, 1.218 Mg/m^3
F(000)	1288
Absorption coefficient	0.580 mm^{-1}
Temperature	100(2) K
Wavelength	1.54184 Å
Crystal mounting:	on a glass fibre, in oil, fixed in cold N_2 stream
On the diffractometer:	
Theta range for data collection	7.761 to 69.999 °
Limiting indices	-14<=h<=14, -14<=k<=21, -22<=l<=20
Completeness to theta = 67.684	99.4 %
Absorption correction	Semi-empirical from equivalents
Max. and min. transmission	1.00000 and 0.64047
Reflections collected (not including absences)	46681

No. of unique reflections 12225 [R(int) for equivalents = 0.084]

No. of 'observed' reflections ($I > 2\sigma_I$) 10040

Structure determined by: dual methods, in SHELXT

Refinement: Full-matrix least-squares on F^2, in SHELXL

Data / restraints / parameters 12225 / 0 / 865

Goodness-of-fit on F^2 1.068

Final R indices ('observed' data) R_1 = 0.059, wR2 = 0.159

Final R indices (all data) R_1 = 0.069, wR_2 = 0.166

Reflections weighted:

$w = [\sigma^2(Fo^2)+(0.0938P)^2+0.5670P]^{-1}$ where $P=(Fo^2+2Fc^2)/3$

Extinction coefficient n/a

Largest diff. peak and hole 0.42 and -0.32 e.Å^{-3}

Location of largest difference peak near H(76f)

Crystal structure analysis of 33

Crystal data: $C_{82}H_{91}N_3O_5$, M = 1198.57. Triclinic, space group P-1 (no. 2), a = 11.5732(2), b = 17.8975(3), c = 18.0680(3) Å, α = 117.737(2), β = 97.567(2), γ = 91.4000(10) °, V = 3267.86(11) $Å^3$. Z = 2, Dc = 1.218 g cm^{-3}, F(000) = 1288, T = 100(2) K, μ(Cu-Kα) = 5.80 cm^{-1}, λ(Cu-Kα) = 1.54184 Å..

The crystal was a colourless shard. From a sample under oil, one, *ca* 0. x 0. x 0. mm, was mounted on a small loop and fixed in the cold nitrogen stream on a Rigaku Oxford Diffraction XtaLAB Synergy diffractometer, equipped with Cu-Kα radiation, HyPix detector and mirror monochromator. Intensity data were measured by thin-slice ω-scans. Total no. of reflections recorded, to $θ_{max}$ = 70.0 °, was 46,681 of which 12,225 were unique (Rint = 0.084); 10,040 were 'observed' with I > $2σ_I$.

Data were processed using the CrysAlisPro-CCD and -RED (Rigaku Oxford Diffraction Ltd., Abingdon, UK (2018)) programs. The structure was determined by the intrinsic phasing routines in the SHELXT program[33] and refined by full-matrix least-squares methods, on F^2's, in SHELXL.[34] There is disorder in two of the hexyl chains in the molecule, each resolved (mostly) into two alternative orientations. The non-hydrogen atoms with site occupancy factors greater than 0.5 were refined with anisotropic thermal parameters; the minor occupy atoms were refined isotropically. The hydrogen atom on N(1) was located in a difference map and was refined freely. The remaining hydrogen atoms were included in idealised positions and their Uiso values were set to ride on the Ueq values of the parent carbon atoms. At the conclusion of the refinement, wR_2 = 0.166 and R_1 = 0.069 (2B) for all 12,225 reflections weighted w = $[σ^2(F_o^2) + (0.0938 P)^2 + 0.5670 P]^{-1}$ with P = $(F_o^2 + 2F_c^2)/3$; for the 'observed' data only, R_1 = 0.059.

In the final difference map, the highest peak (*ca* 0.4 e$Å^{-3}$) was near H(76f).

Notes on the structure

N(2) of one of the isoindole groups has a bonded hydrogen atom, clearly identified in difference maps and the donor to an intramolecular hydrogen bond, N(2)-H(2)…N(42).

The pyrene and triphenylene ring systems are essentially planar and parallel, and lie superposed with C(30) lying 3.501 Å under C(65).

Two of the five O-hexyl chains have the all-*trans* conformation; one has a *cis*-conformation at O(271)-C(272)-C(273)-C(274) but is otherwise all-*trans*; the chains of O(261) and O(321) show disorder, both fully resolved.

We note that there are no very short intermolecular contacts; the shortest contacts are at normal van der Waals' distances.

Compound 36a

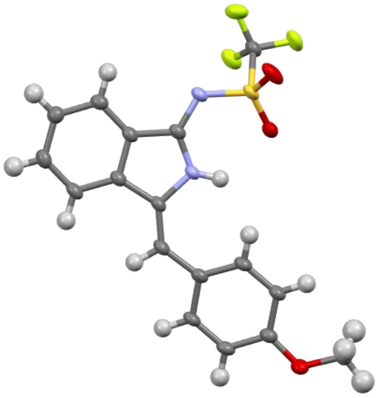

Crystal data and structure refinement for 36a

Identification code	36a
Elemental formula	C17 H13 F3 N2 O3 S
Formula weight	382.35
Crystal system, space group	Triclinic, P -1
Unit cell dimensions	a = 7.0556(3) Å α = 107.878(4) °<br>b = 10.5060(5) Å β = 103.794(4) °<br>c = 12.6271(5) Å γ = 101.674(4) °
Volume	825.77(7) $Å^3$
Z, Calculated density	2, 1.538 Mg/m^3
F(000)	392
Absorption coefficient	2.242 mm^{-1}
Temperature	100.01(10) K
Wavelength	1.54184 Å
Crystal mounting:	on a glass fibre, in oil, fixed in cold N_2 stream
On the diffractometer:	
Theta range for data collection	7.769 to 69.908 °
Limiting indices	-8<=h<=8, -12<=k<=12, -15<=l<=13
Completeness to theta = 67.684	98.2 %
Absorption correction	Semi-empirical from equivalents
Max. and min. transmission	1.00000 and 0.68226
Reflections collected (not including absences)	7870

No. of unique reflections 3042 [R(int) for equivalents = 0.048]

No. of 'observed' reflections ($I > 2\sigma_I$) 2659

Structure determined by: dual methods, in SHELXT

Refinement: Full-matrix least-squares on F^2, in SHELXL

Data / restraints / parameters 3042 / 0 / 235

Goodness-of-fit on F^2 1.074

Final R indices ('observed' data) $R_1 = 0.055$, $wR_2 = 0.153$

Final R indices (all data) $R_1 = 0.060$, $wR_2 = 0.157$

Reflections weighted:
$w = [\sigma^2(Fo^2)+(0.1025P)^2+0.2160P]^{-1}$ where $P=(Fo^2+2Fc^2)/3$

Extinction coefficient n/a

Largest diff. peak and hole 0.55 and -0.57 e.Å^{-3}

Location of largest difference peak near C(13)

Crystal structure analysis of 36a

Crystal data: $C_{17}H_{13}F_3N_2O_3S$, M = 382.35. Triclinic, space group P-1 (no. 2), a = 7.0556(3), b = 10.5060(5), c = 12.6271(5) Å, α = 107.878(4), β = 103.794(4), γ = 101.674(4) °, V = 825.77(7) Å^3. Z = 2, Dc = 1.538 g cm^{-3}, F(000) = 392, T = 100.01(10) K, μ(Cu-Kα) = 22.4 cm^{-1}, λ(Cu-Kα) = 1.54184 Å.

The crystal was a colourless shard. From a sample under oil, it was mounted on a small loop and fixed in the cold nitrogen stream on a Rigaku Oxford Diffraction XtaLAB Synergy diffractometer, equipped with Cu-Kα radiation, HyPix detector and mirror monochromator. Intensity data were measured by thin-slice ω-scans. Total no. of reflections recorded, to θ_{max} = 70.0°, was 7870 of which 3042 were unique (Rint = 0.048); 2659 were 'observed' with $I > 2\sigma_I$.

Data were processed using the CrysAlisPro-CCD and -RED (Rigaku Oxford Diffraction Ltd., Abingdon, UK (2018)) programs. The structure was determined by the intrinsic phasing routines in the SHELXT program[33] and refined by full-matrix least-squares methods, on F^2's, in SHELXL.[34] The non-hydrogen atoms were refined with anisotropic thermal parameters. The hydrogen atom on N(2) was located in a difference map. All the hydrogen atoms were included in idealised positions and their Uiso values were set to ride on the Ueq values of the parent carbon or nitrogen atoms. At the conclusion of the refinement, wR_2 = 0.157 and R_1 = 0.060 (2B) for all 3042 reflections weighted $w = [\sigma^2(F_o^2) + (0.1025\ P)^2 + 0.2160\ P]^{-1}$ with $P = (F_o^2 + 2F_c^2)/3$; for the 'observed' data only, R_1 = 0.055.

In the final difference map, the highest peak (*ca* 0.55 eÅ^{-3}) was near C(13).

Notes on the structure

The isoindole group forms the central plane of the molecule. The normal to the phenyl group is rotated 14.3 ° from that of the isoindole group and the SO_2-CF_3 group provides the only significantly displaced atoms from the major planar units.

The pyrrole hydrogen atom was recognised in difference maps and forms a good intramolecular hydrogen bond with O(121).

Molecules are stacked, principally through the π…π interactions between overlapping isoindole rings, in columns parallel to the *a* axis, with interplanar distances of 3.000 and 3.319 Å, either side of the isoindole plane. The phenyl ring partially overlaps its symmetry neighbour on one side at a distance of 3.59 Å; there are no π…π interactions on the opposing side.
